# Intertemporal Pavlovian biases and links with mental health in the general population

**DOI:** 10.3758/s13415-025-01326-9

**Published:** 2025-08-11

**Authors:** Floor Burghoorn, Anouk Scheres, Karin Roelofs, Bernd Figner

**Affiliations:** 1 Behavioural Science Institute, Radboud University, Nijmegen, the Netherlands; 2 Department of Psychiatry and Psychotherapy, Faculty of Medicine, University of Tübingen, Tübingen, Germany; 3 Donders Institute for Brain, Cognition and Behaviour, Radboud University, Nijmegen, the Netherlands

**Keywords:** Intertemporal choice, Delay discounting, Reinforcement learning, Pavlovian bias, Mental health, Computational psychiatry

## Abstract

Previous research has found the anticipation of immediate (compared with delayed) rewards to enhance instrumental approach behaviour and interfere with instrumental inhibition. This so-called intertemporal Pavlovian bias has been proposed as a mechanism contributing to impatient actions. In the present preregistered study, we aimed to replicate this intertemporal Pavlovian bias effect, and examined whether individual differences in the strength of this bias were associated with mental health symptoms characterized by increased or decreased intertemporal impatience. A sample of 389 participants completed an intertemporal go/no-go learning task, as well as questionnaires that assessed symptoms of alcohol use disorder, nicotine dependence, depression, anxiety, attention-deficit/hyperactivity disorder (ADHD), disordered eating, impulsivity, and body mass index. We replicated the intertemporal Pavlovian bias effect on the probability of go responding (a response bias), and reinforcement learning models again pointed to a Pavlovian cue-response bias as computational mechanism driving this effect. Contrary to our expectations, the response bias was not associated with mental health symptoms. We also observed an intertemporal Pavlovian bias effect on the vigour of correct go responses, with faster responses in anticipation of immediate rewards (a response time or RT bias). A stronger RT bias was associated with ADHD hyperactivity/impulsivity and nonplanning impulsivity symptoms, while a reversed RT bias (with faster incorrect go responses in anticipation of delayed rewards) was associated with eating disorder symptoms and total and motor impulsivity. Together, our results support the robustness of the intertemporal Pavlovian response bias and point towards the possible relevance of the RT bias for several mental health problems.

**Supplementary Information:**

The online version contains supplementary material available at 10.3758/s13415-025-01326-9.

Intertemporal decisions refer to choices between smaller short-term and larger long-term outcomes. For instance, we may choose between watching another episode of our favourite series versus going back to writing a difficult paper to obtain our degree, or we may be tempted to smoke a cigarette instead of abstaining to improve our long-term health. Individuals vary in the degree of *intertemporal impatience* they exhibit in these situations, i.e., their tendency to choose short-term over long-term outcomes. In research, the most common approach to quantify intertemporal impatience is to ask participants to make a series of choices between sooner-smaller and later-larger monetary rewards (also known as delay discounting tasks, referring to the subjectively discounted value of a reward as its delay until delivery increases; Lempert et al., 2018). Intertemporal impatience has been associated with a large variety of maladaptive behaviours and mental health problems, with meta-analyses showing increased impatience in individuals diagnosed with attention-deficit/hyperactivity disorder (ADHD; Jackson & Mackillop, 2016; Marx et al., 2021; Patros et al., 2016; Pauli-Pott & Becker, 2015), substance and gambling addiction (MacKillop et al., 2011), obesity (Amlung et al., 2016), major depressive disorder, schizophrenia, borderline personality disorder, bipolar disorder, bulimia nervosa, binge-eating disorder (Amlung et al., 2019), and decreased impatience in individuals diagnosed with anorexia nervosa (Amlung et al., 2019), compared with individuals without mental health diagnoses. Following these findings, intertemporal impatience has been proposed as a transdiagnostic process that may form a risk factor contributing to the development and maintenance of these disorders (supported by studies showing an etiological role of impatience), and may explain some of the comorbidity between disorders (Amlung et al., 2017, 2019; Bickel et al., 2012, 2019; Lempert et al., 2018; Scheres et al., 2024). In addition to these differences between patients and controls, studies have observed continuous associations between intertemporal impatience and the severity of mental health problems in general population samples, in line with the proposed dimensionality of intertemporal impatience and mental health constructs (Amlung et al., 2016, 2017; Burghoorn et al., 2025; Keidel et al., 2024; Levin et al., 2018; Levitt et al., 2022).

## Intertemporal Pavlovian bias

The relevance of intertemporal impatience across mental health problems raises the question about which psychological mechanisms may contribute to impatience and thus may be implicated in the behaviours and mental health symptoms that have been characterized by intertemporal impatience. In a recent study, we provided evidence for an *intertemporal Pavlovian bias* as a possible mechanism contributing to impatient intertemporal decisions (Burghoorn et al., 2024). Pavlovian biases refer to the influence of anticipated outcomes (e.g., rewards or punishments), signalled by cues in the environment, on instrumental (often goal-directed) actions. Previous research has demonstrated that the anticipation of a reward elicits a Pavlovian approach response, which is adaptive when approach responses are required to achieve a goal, but maladaptive when inhibitory or withdrawal responses are required to achieve a goal (Guitart-Masip et al., 2014). In contrast, the anticipation of a punishment has been shown to elicit a Pavlovian withdrawal response, enhancing instrumental approach but interfering with instrumental withdrawal. Pavlovian responses may either align or conflict with instrumentally required actions. A key difference between Pavlovian and instrumental action control is that whereas Pavlovian control revolves around relatively simple cue-outcome associations, instrumental control revolves around more complex cue-*action*-outcome associations. Thus, only instrumental actions (particularly when they are goal-directed) are determined by their effect on the desired outcomes or goals. Although Pavlovian actions are often shaped through their general adaptiveness (e.g., it is generally adaptive to approach rewards or reward-predicting cues; Dayan et al., 2006), they can result in actions that persist even when maladaptive to reaching one’s goal (e.g., when a reward requires inhibition). In other words, while instrumental control is computationally costly and flexible, Pavlovian control is computationally cheap and inflexible (Dorfman & Gershman, 2019; Huys et al., 2012; Millner et al., 2018).

Until recently, research on Pavlovian biases exclusively focused on the *valence* and *magnitude* of anticipated outcomes (see Guitart-Masip et al., 2014 for a review), comparing the anticipation of rewards versus punishments (and sometimes also comparing different reward and/or punishment magnitudes). In Burghoorn et al. (2024), however, we demonstrated that the *timing* of the anticipated reward also influenced instrumental actions in a Pavlovian manner. To test this, we developed an intertemporal variant of a go/no-go learning task that orthogonalizes the required instrumental action (go/no-go) and the timing of the reward that is available upon giving the correct response (immediate/delayed). This resulted in four trial types: go to win immediate reward, go to win delayed reward, no-go to win immediate reward, and no-go to win delayed reward trials. The immediate and delayed rewards were preference-matched per participant by using a choice titration procedure, allowing us to test for the effect of reward timing beyond individual differences in subjectively discounted reward value. We observed that the anticipation of immediate rewards increased go responding compared with the anticipation of delayed rewards, thus facilitating instrumental approach but interfering with instrumental inhibition. This intertemporal Pavlovian bias may contribute to the often-experienced difficulty to inhibit ourselves in the face of immediate gratification, possibly at the cost of long-term goals. For instance, the sight of your favourite unhealthy snack may elicit an approach response (i.e., eating the snack), giving you an immediate reward (i.e., the taste of the snack) but undermining your long-term health goal. Note that irrespective of the reward timing, any reward may elicit a Pavlovian approach response and may therefore contribute to intertemporally impatient actions (as also discussed by Dayan et al., 2006; Huys et al., 2012). Importantly, however, the results of Burghoorn et al. (2024) extend this by demonstrating that immediate rewards exert a *stronger* approach response compared with delayed rewards, even when the two rewards are matched based on revealed choice preferences.

## Intertemporal Pavlovian bias and mental health: The present study

In the current study, we asked how such an intertemporal Pavlovian bias may be implicated in those mental health problems that have been characterized by increased or decreased intertemporal impatience. For instance, a stronger difficulty to inhibit one’s response to cues signalling immediate gratification (e.g., a drug or snack), at the cost of long-term (health) goals, may contribute to a vulnerability to develop substance use addiction, obesity, or binge eating episodes. Similarly, a stronger behavioural activation towards distracting but immediately rewarding cues (e.g., an incoming text), at the cost of long-term occupational or educational achievements, may be associated with ADHD symptoms. In contrast, excessive control over Pavlovian responses towards immediate reinforcers (e.g., food) and/or a preoccupation with future (e.g., weight) goals may be associated with symptoms of anorexia nervosa.^1^ Providing support for the general relevance of Pavlovian biases in mental health, several studies have found valence-driven Pavlovian biases (i.e., comparing the anticipation of rewards versus punishments) to be associated with individual differences in substance use (Garbusow et al., 2022), mood and anxiety symptoms (Huys et al., 2016; Mkrtchian et al., 2017; Nord et al., 2018; Peterburs et al., 2022; Scholz et al., 2020; but also see Moutoussis et al., 2018), suicidal thoughts and behaviours (Millner et al., 2018), ADHD hyperactivity/impulsivity symptoms (Geurts, Den Ouden, et al., 2022), borderline personality disorder symptoms (but only for neural Pavlovian bias-related signals, not for behaviour; Geurts, Van Den Heuvel, et al., 2022), schizophrenia (Albrecht et al., 2016; Montagnese et al., 2020; Morris et al., 2015; but also see Metts et al., 2022), and psychopathic tendencies (Geurts, Von Borries, et al., 2022), although the direction of the reported statistically significant associations (i.e., positive or negative) has been somewhat inconsistent.

The present study had two main goals. First, using the intertemporal go/no-go task developed previously (Burghoorn et al., 2024), we aimed to replicate the intertemporal Pavlovian bias effect, thereby examining its robustness. Second, we investigated the associations between this intertemporal Pavlovian bias and symptoms of alcohol use disorder, nicotine dependence, ADHD, depression, trait anxiety, disordered eating, body mass index (BMI), and impulsivity, all of which have previously been associated with increased or decreased intertemporal impatience. We investigated these associations in a general population sample, thus treating mental health and intertemporal impatience as dimensional constructs, and tested for their continuous associations. Third and finally, while not a central goal of the present study, we tested whether the associations between the mental health symptoms and the level of intertemporal impatience (as assessed using a choice titration procedure, explained further below) were similar to those previously reported in the literature.

## Study hypotheses

We expected to replicate the intertemporal Pavlovian bias effect and thus predicted that go responding in the go/no-go task would increase in anticipation of an immediate reward (i.e., in the immediate reward trials) compared with the anticipation of a preference-matched delayed reward (i.e., in the delayed reward trials).

Next, following the literature on intertemporal impatience and mental health discussed earlier and the proposed role of the intertemporal Pavlovian bias as mechanism of impatience, we expected the strength of the intertemporal Pavlovian bias to be associated with increased symptoms of alcohol use disorder, nicotine dependence, ADHD, depression, and BMI. For several of these mental health variables, we not only assessed total symptom scores, but additionally distinguished between different symptom subscales. Specifically, for alcohol addiction, we distinguished between general alcohol use and alcohol-related problems, as research suggests them to be separate constructs (Horváth et al., 2023), and because we expected to observe more interindividual variation in general alcohol use compared with alcohol-related problems. Similarly, for nicotine addiction, we distinguished between nicotine dependence in the full sample, smoking status (i.e., whether someone smokes or not), and nicotine dependence in a subset of smokers. For ADHD, we distinguished between hyperactivity/impulsivity symptoms and inattention symptoms, as recommended by Jackson and Mackillop (2016). We hereby explored the possibility that different subsets of symptoms may be driven by different psychological mechanisms, thus accounting for within-disorder heterogeneity of symptoms and underlying mechanisms. Scheres et al. (2008) observed hyperactivity-impulsivity but not inattention symptoms to be associated with increased intertemporal impatience, raising the possibility that the former is more strongly associated with the intertemporal Pavlovian bias than the latter.

For disordered eating, we distinguished between symptoms reflecting a lack of control over eating and other eating disorder symptoms (revolving around food restriction and a preoccupation with weight loss). We expected a lack of control over eating to be associated with a stronger intertemporal Pavlovian bias, reflecting a stronger effect of cues signalling immediate gratification (e.g., food rewards) on behaviour. This aligns with research showing increased intertemporal impatience in bulimia nervosa and binge-eating disorders, both of which are characterized by uncontrolled binge-eating episodes (Amlung et al., 2019). We did not have a directional hypothesis for the remaining eating disorder symptoms. Because we expected these symptoms to be positively correlated with the symptoms reflecting a lack of control overeating, one may expect a similarly increased Pavlovian bias. At the same time, the strong focus on food restriction and weight loss that characterizes these symptoms may require strong control over Pavlovian responses towards immediate rewards, as well as a strong focus on delayed rewards, possibly resulting in a reduced or even reversed intertemporal Pavlovian bias. This is consistent with previously reported negative associations between anorexia nervosa—particularly the restricting subtype—and intertemporal impatience (see Amlung et al., 2019 and Lempert et al., 2018 for reviews).

Findings on the association between intertemporal impatience and anxiety have been mixed; studies showed increased impatience (Keidel et al., 2024; Rounds et al., 2007; Xia et al., 2017; Zhang et al., 2020), decreased impatience (Steinglass et al., 2017), or no association with impatience (Jenks & Lawyer, 2015; Takahashi, 2004; Worthy et al., 2014). Even within studies, findings have been mixed, depending on the type of analytic approach used (Levitt et al., 2022) or the type of anxiety assessed (Levin et al., 2018). Similarly, while some studies on valence-driven Pavlovian biases observed an increased Pavlovian bias in association with anxiety—albeit in the punishment domain (Mkrtchian et al., 2017; Peterburs et al., 2022)—others found a decreased bias (Scholz et al., 2020), the latter of which was theorized to result from a more ruminative and careful decision style. Because the present study only included rewards (not punishments), and following the inconsistency in previous research findings on anxiety, intertemporal impatience, and valence-driven Pavlovian biases, we did not have a directional hypothesis regarding the association between the intertemporal Pavlovian bias and anxiety.

Finally, we expected the intertemporal Pavlovian bias to be associated with increased impulsivity, reflecting an impaired ability to override the Pavlovian approach response elicited by immediate rewards. This hypothesis follows the strong conceptual link between impulsivity and intertemporal impatience, as well as several studies showing an empirical association between the two (Baumann & Odum, 2012; Jauregi et al., 2018; Keidel et al., 2024; Malesza & Kalinowski, 2021; Malesza & Ostaszewski, 2016; Zhou et al., 2021). Impulsivity was assessed by using the Barratt Impulsiveness Scale (BIS-11; Patton et al., 1995), which, in addition to a total score, results in separate scores for attentional impulsivity, motor impulsivity, and nonplanning impulsivity. Because the intertemporal Pavlovian bias involves a conditioned approach (go) response, we expected the bias to be most strongly associated with motor impulsivity.

Our hypotheses on the association between mental health and intertemporal impatience (as assessed using the choice titration procedure) followed the hypotheses on mental health and the intertemporal Pavlovian bias described above.

## Methods

The study’s research questions, hypotheses, design, sample size, and analyses were preregistered on the Open Science Framework (https://osf.io/4qczy). The OSF page also contains the study materials, data, and analysis code (https://osf.io/p5bz9/).

### Participants

Participants were recruited on Prolific (https://www.prolific.com/) if they indicated to be fluent in English, live in a country that uses euros as its currency (as study rewards were presented in euros), have normal or corrected-to-normal vision, have normal colour vision, and have an approval rate on Prolific of at least 95%. Of the 435 participants who took part in the study, 46 were excluded from data analysis, either because they did not meet our preregistered data quality criteria (*n* = 44, see Supplementary Information 1 [S1] for details), because they did not complete the entire study (*n* = 1), or did not complete the study in English (*n* = 1). A simulation-based power analysis showed that the remaining sample of 389 participants (140 females, 243 males, 5 nonbinary, 1 other; *M*_age_ = 31.37, *SD*_age_ = 9.07) would provide 90–95% power to replicate the intertemporal Pavlovian bias effect observed in Burghoorn et al., (2024; unstandardized effect of reward of *b* = 0.17 on log-odds scale), and at least 95% power to replicate an interaction between the intertemporal Pavlovian bias and total alcohol use disorder scores observed in a pilot study (*N* = 51; unstandardized interaction of *b* = 0.34 on log-odds scale; smaller interaction effect sizes of 0.25 and 0.20 would be detected with 87% and 77% power, respectively). Following our preregistered trial-level exclusion criteria (see S1), 0.32% of the go/no-go trials of the final sample were excluded.

The study fell under a research line that received ethics approval from the local institutional review board prior to data collection (number: ECSW-2024–053), and the study was performed in accordance with the ethical standards of the Declaration of Helsinki. Digital informed consent was obtained from all individual participants. Participation was compensated with £5.25 (Prolific uses Great British Pounds as its currency). In addition, participants took part in a performance-contingent lottery, in which they could win one of the rewards they earned during the go/no-go task (described below).

### General procedure

**Fig. 1:**
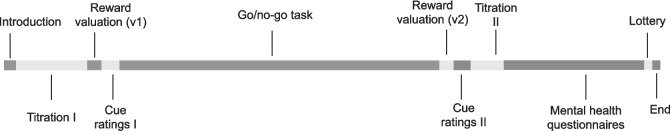
Experimental timeline. *Note.* Experimental timeline, displaying the order of tasks administered. Whether the reward valuation task was administered before (v1) or after (v2) the go/no-go task, and whether the valuation task was a rating or ranking task, was counterbalanced across participants

The experimental procedure was programmed in jsPsych (version 7.0.0; de Leeuw & Gilbert, 2023) and could be completed in a Mozilla Firefox, Safari, or Microsoft Edge browser on a desktop or laptop computer. The experimental timeline is displayed in Fig. 1. The complete experiment took approximately 35 min.

### Choice titration I

The choice titration procedure was used to derive a participant-specific immediate reward that was preference-matched to a delayed reward of €28 in 120 days. Details are reported in Burghoorn et al. (2024). Summarized, participants first completed the Monetary Choice Questionnaire (Kirby et al., 1999), which consists of 27 choices between varying immediate and delayed monetary rewards. Choices on this task were used to derive a participant-specific discount rate, which was then used to compute the starting amount of the immediate reward in the subsequent adaptive choice titrator. Each trial of the adaptive choice titrator offered a choice between an immediate reward (€X today, with X being an even integer between €0 and €28) versus a fixed delayed reward of €28 in 120 days. If a participant chose the immediate [delayed] reward, the immediate reward amount on the next trial was decreased [increased] with €2. The titrator continued until participant reached a stable window of six trials during which the immediate reward did not deviate by more than €2. Participants who failed to reach stability after 50 trials were excluded from data analyses (*n* = 5). Participants who indicated to prefer €0 today over €28 in 120 days were asked to confirm their choice in a subsequent, identical trial. If they again preferred €0, they were excluded from the analyses (*n* = 1), because this indicated that they preferred not to receive any reward, undermining a critical premise of the study. The titration procedure was incentivized through a lottery at the end of the experiment, in which participants had the chance of winning a monetary reward that was determined by their choice preferences (described below).

### Intertemporal Go/No-Go task

**Fig. 2:**
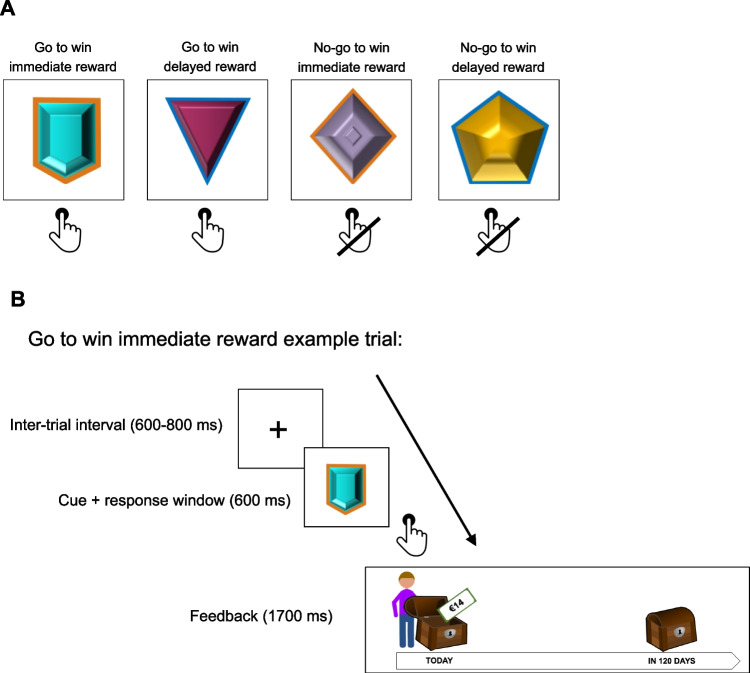
Go/No-Go task. *Note.* Design of the go/no-go task. **A:** The go/no-go task consisted of four conditions, each signalled by a unique visual cue: go to win immediate reward, go to win delayed reward, no-go to win immediate reward, no-go to win delayed reward. The cue signalled the required instrumental action (go/no-go) and the reward available upon giving a correct response (immediate/delayed). Participants had to learn the required instrumental action by trial and error; the reward was instructed through the coloured edge around the cue, and could also be learned by trial and error. Cues were randomly assigned to conditions, and which cue edge (blue/orange) indicated which reward (immediate/delayed) was counterbalanced across participants. Each condition was presented 50 times in pseudorandom order (i.e., in random order except that the same condition could not be presented more than twice in a row). **B:** Example of a go to win immediate reward trial. The trial started with a fixation cross (inter-trial interval), after which the cue was presented. Upon cue presentation, participants had to respond within 600 ms, after which feedback was provided. If participants gave a correct response for an immediate reward cue (such as that presented in B), the immediate reward was presented to go into a chest close to the participants’ agent on a timeline. If participants gave a correct response for a delayed reward cue (not presented in B), the delayed reward was presented to go into a chest far away from the participants on the timeline. If participants gave an incorrect response (regardless of the cue), both chests remained closed. Feedback was probabilistic; in only 80% of the trials, the feedback presented corresponded to the correctness of the participant’s response

Next, participants completed the intertemporal go/no-go task as detailed in Burghoorn et al., (2024; originally adapted from Scholz et al., 2022) and as displayed in Fig. 2. The task was framed as a gem game, during which participants had to learn which two gems (i.e., cues) to collect (go response) and which two gems to leave behind (no-go response). For two of the four gems, a correct response resulted in the delayed larger reward of €28 in 120 days. For the other two gems, a correct response resulted in the participant-specific immediate reward of €X today. For all four gems, an incorrect response resulted in no reward. Orthogonalizing the required action (go/no-go) and available reward (immediate/delayed) resulted in four conditions: go to win immediate reward trials; go to win delayed reward trials; no-go to win immediate reward trials; and no-go to win delayed reward trials. For each participant, the four cues were randomly assigned to conditions. The required response (go/no-go) for each of the cues had to be learned by trial and error. Although the available reward (immediate/delayed) could also be learned by trial and error, it was additionally signalled by a coloured edge (orange/blue) around the cue. The purpose of this coloured edge was to increase the salience of the available reward (immediate/delayed), and to ensure that any reward effects would be present early on in the task (hereby following Burghoorn et al., 2024; Scholz et al., 2022; Swart et al., 2017, 2018). Which edge colour signalled which reward was counterbalanced across participants and was instructed to the participants before the start of the task.

Upon cue presentation, participants had 600 ms to make a response by pressing the space bar (go response) or doing nothing (no-go response). After the response window, the outcome was presented for 1,700 ms, by presenting a reward going into a reward chest that was either close to the participant on a timeline (immediate reward) or far away on the timeline (delayed reward), or by presenting only closed chests (no reward). Outcome feedback was probabilistic, such that in 80% the trials, the outcome presented to the participant was consistent with the correctness of their response. Each of the four conditions was presented 50 times in pseudorandom order (i.e., random order with the exception that the same condition could not be presented more than twice in a row). The 200 trials were divided in four blocks of 50 trials, divided by 20-s breaks.

Participants received extensive task instructions and were required to reach 80% performance in a round of five practice trials before proceeding to the full task. Participants who required more than six practice rounds were excluded from the analyses (*n* = 2). The task included four attention checks (separated by a minimum of 25 trials), during which a crosshair target appeared on the screen for 1,000–2,000 ms (jittered), and participants had to press the space bar within 1,500 ms upon disappearance of the target. To incentivize task performance, participants were informed that they would be entered into a lottery at the end of the experiment, during which they had the chance to win the outcome they received on one randomly selected go/no-go trial (hereby incentivizing response accuracy) and that their response speed increased their chance of winning the lottery (hereby incentivizing response speed). See Burghoorn et al. (2004) for the equation used to determine the per-participant lottery chance; 13 participants won the lottery.

### Mental health measures

The instruments used to assess the mental health symptoms, and their descriptive statistics, are outlined in Table 1. A detailed description of all questionnaires can be found in S2. The order in which the questionnaires were administered was randomly determined per participant. The STAI-T-5 and BIS-11 contained one attention check, instructing participants which answer to give. After completing the mental health questionnaires, participants also completed a few questions on their menstrual cycle (if applicable); these data were not of interest (and therefore not analysed) for the present study.

**Table 1:** Mental health measures

Variable	Scale	Possible range	Observed range	*M* (*SD*)	*Mdn*	Cronbach’s α
Total alcohol use disorder	AUDIT Saunders et al. (1993)	0–40	0–29	3.61 (4.20)	2	.86
Alcohol use	AUDIT	0–12	0–12	2.43 (2.15)	2	.75
Alcohol problems	AUDIT	0–28	0–19	1.18 (2.49)	0	.81
Smoking status	“Do you smoke (e-)cigarettes?”	Yes / No	16% smokers			
Nicotine dependence in full sample	FTND Heatherton et al. (1991)	0–11	0–8	0.57 (1.58)	0	.86^a^
in smokers (*n* = 63)		0–10	0–7	2.54 (2.23)	2	
Depression	DASS-21 Lovibond and Lovibond (1995)	0–21	0–21	5.55 (5.23)	4	.93
Trait anxiety	STAI-T-5 Zsido et al. (2020)	0–15	0–15	5.98 (3.35)	6	.85
Total ADHD	ASRS-v1.1 Kessler et al. (2005)	0–72	0–62	27.42 (9.89)	27	.88
ADHD inattention symptoms	ASRS-v1.1	0–36	0–31	14.58 (5.69)	14	.85
ADHD hyperactivity / impulsivity symptoms	ASRS-v1.1	0–36	0–31	12.84 (5.58)	13	.80
Total disordered eating	EDE-QS Gideon et al. (2016)	0–36	0–29	7.26 (6.70)	5	.88
Lack of control over eating	EDE-QS	0–6	0–6	0.86 (1.28)	0	.75
Remaining eating disorder symptoms	EDE-QS	0–30	0–26	6.40 (5.82)	5	.85
Total impulsivity	BIS-11 Patton et al. (1995)	0–90	5–64	30.07 (10.42)	29	.86
Attentional impulsivity	BIS-11	0–24	0–24	8.59 (3.91)	9	.76
Motor impulsivity	BIS-11	0–33	1–21	9.65 (4.07)	9	.68
Non-planning impulsivity	BIS-11	0–33	1–26	11.84 (5.04)	12	.77
Body Mass Index^b^	$\frac{\mathrm{weight}}{{(\mathrm{height}/100)}^{2}}$		16.07 – 61.86	24.17 (4.78)	23.15	

Overview of the mental health variables assessed in the study, the instruments used to assess them, and their descriptive statistics. *M* = mean, *SD* = standard deviation, *Mdn* = median. Cronbach’s α was computed for all scales consisting of multiple items

^a^ Given the limited subsample of smokers, we only computed Cronbach’s α in the total sample

^b^One impossible BMI score was removed from the sample. Therefore, all analyses involving BMI scores were performed on 388 participants

### Secondary measures

In addition to the choice titration, go/no-go task, and mental health questionnaires, which formed the three main parts of the experiment, we administered several other short tasks, described in the following sections.

#### Reward valuations

The choice titration procedure resulted in an immediate and delayed reward that were matched based on revealed choice preferences. To examine whether the two rewards were also valued similarly outside a choice context, we administered a separate reward valuation task. We included two versions of this task, a rating and ranking version, allowing us to compare valuations across different preference elicitation methods. Which of the two versions of the valuation task a participant completed, and whether they completed this before or after the go/no-go task, was counterbalanced across participants.

##### Rating task

The rating task was identical to that used in Burghoorn et al. (2024). Participants were asked to rate how attractive they found each of the two rewards, one by one, using a slider on a visual analogue scale ranging from very unattractive (0, left endpoint) to very attractive (100, right endpoint). The left endpoint additionally included as anchor a delayed reward that was €1 lower in amount than the immediate reward of the preference-matched reward pair, and 1 day longer in delay than the delayed reward (i.e., 121 days), hereby representing a relatively very unattractive reward. The right endpoint included as anchor an immediate reward that was €1 higher in amount than the delayed reward of the preference-matched reward pair (i.e., €29), representing a relatively very attractive reward. The two reward ratings were presented in random order.

##### Ranking task

The ranking version of the valuation task was adapted from Luo et al. (2009). Participants were asked to rank the two rewards based on how attractive they found each of them. They were instructed to give the reward that they found most attractive rank 1 and the reward that they found least attractive rank 2, or if they found both rewards equally attractive to give both rewards rank 1. In line with Luo et al. (2009), participants were instructed not to think about which reward they would choose if they compared them but simply to think about how attractive they found each reward. The two rewards were simultaneously presented on the screen; which reward was presented on the top or bottom of the screen was randomly determined.

#### Cue ratings

To examine pre-existing differences in subjective valuation of the cues (i.e., gems) used in the go/no-go task, we asked participants to rate how attractive they found each of the cues. Ratings were provided using a slider on a visual analogue scale ranging from very unattractive (0, left endpoint) to very attractive (100, right endpoint). The cue rating task was again presented after the go/no-go task, allowing us to examine whether the cue ratings changed over the course of the experiment. In both cue rating tasks, the cues were presented in random order.

#### Choice titration II

To examine whether the degree of intertemporal impatience, as assessed using the first choice titrator, remained stable across the experiment, we again administered the adaptive part of the choice titration procedure after the go/no-go task. The starting value of the immediate reward was identical to the adaptive titrator administered before the go/no-go task.

### Data analyses

#### Statistical models

The majority of the data were analysed with Bayesian mixed-effects models, using the *brms* package (version 2.21.0; Bürkner, 2018) in R (version 4.4.1; R Core Team, 2024). The statistical models used for our primary analyses are outlined below; the models for all secondary analyses are specified in the respective results or Supplementary Information sections. In all models, brms’ weakly informative default priors were used, categorical predictors were sum-to-zero coded, and continuous predictors were mean-centered. A Gaussian model family was used unless mentioned otherwise. We accounted for by-participant random variation using a maximal random-effects structure. Effects were denoted as statistically significant if the 95% highest density interval (HDI) did not include 0 (except when the HDI levels were corrected for multiple testing, as detailed below). If we computed Pearson correlations, these were denoted as statistically significant when *p* < 0.05 (except when correcting for multiple testing). Visualizations of the results were created using the packages *emmeans* (version 1.10.3; Lenth, 2019) and *ggplot2* (version 3.5.1; Wickham, 2016).

**Intertemporal Pavlovian bias models**

##### *Response bias*

Our main statistical model on the intertemporal Pavlovian bias included response (go/no-go) as dependent variable, a fixed intercept, required action (go/no-go), reward (immediate/delayed), task block (1–4, modelled as centered linear predictor), and their interactions as fixed effects. Random effects included a random intercept, a random slope for all fixed effects, and all random correlations varying over participants. Responses were modelled with a Bernoulli model family to account for their binary nature.

##### *RT bias*

As preregistered, we also explored whether any intertemporal Pavlovian bias effect was observed on the response times (RTs), reflecting an effect of the available reward on response invigoration. To this end, we reran our main intertemporal Pavlovian bias model, but with response times (from both correct and incorrect go responses) as dependent variable. A shifted lognormal family was used to account for the non-normal RT distribution.

##### Mental health moderation

To examine whether the intertemporal Pavlovian bias was associated with the mental health symptoms, we tested whether the mental health variables moderated the Pavlovian bias. To this end, we reran our Pavlovian bias models (both for responses and RTs), but this time included a mental health score as additional fixed effect, allowing it to interact with all other fixed effects. To prevent convergence issues resulting from overly large models, we ran a separate model for each mental health variable, and corrected for multiple testing by applying a Benjamini–Hochberg correction to the HDIs (with a false discovery rate of 5%; Benjamini & Hochberg, 1995). Our main test involved the two-way interaction between the reward effect and the mental health score. We additionally examined whether this two-way interaction was further moderated by the required action, task block, or both.

##### Intertemporal impatience and mental health

We also tested whether the level of intertemporal impatience, as assessed using the first choice titrator, was associated with any of the mental health variables, in line with the literature on intertemporal impatience and mental health (Amlung et al., 2019; Lempert et al., 2018). Intertemporal impatience was quantified as the reward amount of the immediate reward preference-matched to the delayed reward of €28 in 120 days. Because a higher immediate reward amount indicates more *patience*, we reverse-coded these patience scores to create an *impatience* score (1–28, with higher scores indicating more impatience). We subsequently computed the Pearson correlations between the immediate reward amount derived from the choice titrator, and the mental health variables. A Benjamini–Hochberg correction was applied to correct for multiple testing. Finally, we computed the bivariate correlations between all mental health variables to gain insight into possible comorbidity between mental health symptoms.

#### Reinforcement learning models

To study the computational mechanisms that may underlie the intertemporal Pavlovian response bias, and its possible association with mental health symptoms, we fitted a series of nested reinforcement learning (RL) models to the data of the go/no-go task (following Burghoorn et al., 2024, inspired by Guitart-Masip et al., 2012b). The first and most simple model was a Rescorla-Wagner model (M0), in which action weights (*w*_*t*_) are fully determined by action values (*Q*_t_).:

1 $${w}_{t}\left({a}_{t},{s}_{t}\right)={Q}_{t}\left({a}_{t},{s}_{t}\right)={Q}_{t-1}\left({a}_{t},{s}_{t}\right)+\alpha \left({r}_{t-1}-{Q}_{t-1}\left({a}_{t},{s}_{t}\right)\right)$$

Action values are updated on a trial-by-trial basis, based on prediction errors, i.e., the difference between the expected reward (*Q*_*t-1*_) and the obtained reward (*r*_*t*−1_), scaled by the learning rate (α). A softmax function was used to transform action weights into the probability of making a go response (*p*). The inverse temperature parameter τ reflects the response stochasticity, i.e., the degree to which responses are determined by action weights:

2 $$p\left({a}_{t},{s}_{t}\right)=\left[\frac{\mathrm{exp}(\uptau {w}_{t}({a}_{t}|{s}_{t})}{{\sum }_{a{\prime}}\mathrm{exp}(\uptau {w}_{t}(a{\prime}|{s}_{t})}\right]$$

The next model, M1, includes an extended softmax function with ξ that captures irreducible noise in action selection, due to, e.g., motor or attentional lapses:

3 $$p\left({a}_{t},{s}_{t}\right)=\left[\frac{\mathrm{exp}(\uptau {w}_{t}({a}_{t}|{s}_{t})}{{\sum }_{a{\prime}}\mathrm{exp}(\uptau {w}_{t}(a{\prime}|{s}_{t})}\right] (1-\upxi )+ \frac{\upxi }{2}$$

M2 adds a go bias parameter *b* to the computation of the action weight, capturing a general tendency to give go responses:

4 $$w_t\left(a_t,s_t\right)=\left\{\begin{array}{lc}Q_{t\;}(a_{t,\;}s_t)\;+\;b&\;\;\;\;\;\;\;\;\;\;\;\;if\;a_{t\;}\;=go\\Q_{t\;}(a_{t,\;}s_t)&else\end{array}\right.$$

M3 captures the hypothesized intertemporal Pavlovian bias through a cue-response bias π, which reflects the elicitation of a Pavlovian go response by cues that signal immediate (versus delayed) rewards, implemented through an increased action weight of the go response:

5 $$w_t\left(a_t,s_t\right)=\left\{\begin{array}{lc}Q_t\left(a_t,s_t\right)+b+\pi V\left(s_t\right)&\;\;\;\;\;\;\;\;\;\;if\;a_t\;\;=go\\Q_t\left(a_t,s_t\right)&else\end{array}\right.$$

Here, *V*(${s}_{t}$) represents the reward signalled by the cue. In line with previous research, the values of *V*(${s}_{t}$) were fixed at 1 for immediate rewards and − 1 for delayed rewards, capturing the instruction of the reward identity by the coloured edge around the cue (Burghoorn et al., 2024; Scholz et al., 2022; Swart et al., 2017, 2018; van Nuland et al., 2020).

In Burghoorn et al. (2024), we fitted two additional models, M4 and M5, which reflect alternative and/or complementary computational mechanisms possibly driving the intertemporal Pavlovian bias. We observed that whereas these models provided a slight advantage compared to M3 in terms of model fit, M3 was the only model that showed successful parameter recovery, model recovery, and posterior predictive performance. In the present study, we again fitted M4 and M5 to examine whether the results were consistent with our previous study or whether these models could be successfully validated in the larger sample of the current study. M4 revolves around a learning bias: It reflects enhanced learning of (1) go responses that are followed by immediate (versus delayed) rewards and (2) no-go responses that are followed by delayed (versus immediate) rewards. The learning advantage is reflected by an increased learning rate:

6 $$\alpha=\left\{\begin{array}{cc}\alpha_0&if\left(a_t=go\;\&\;r_t=immediate\right)or\left(a_t=nogo\;\&\;r_t=delayed\right)\\\alpha_1&else\end{array}\right.$$

M5 forms a combination of M3 and M4 by including both the cue-response and the learning bias.

##### Model fitting, comparison, and validation

The models were fitted using maximum a posteriori (MAP) estimation, which aims to find the participant-specific posterior mode. As in Burghoorn et al. (2024), the learning rate and irreducible noise parameters were constrained between 0 and 1, the inverse temperature was constrained between 0 and 50, and the go bias and Pavlovian bias parameters were constrained between − 3 and 3. A *Gamma*(3,0.3) prior was used for the inverse temperature parameter, and a *Gaussian*(0,1) prior was used for the go bias and the cue-response bias parameters. Parameters were optimized with a differential evolution algorithm implemented in the *DEoptim* package (version 2.2.8; Mullen et al., 2011). Models were compared using Akaike’s Information Criterion (AIC), with smaller values indicating a better fit, and model frequency, the proportion of participants for which each model had the lowest AIC. We validated the best-fitting models using parameter recovery, model recovery, and posterior predictive checks.

##### Associations with mental health

We computed Pearson correlations between the parameters of the winning RL model and the mental health variables assessed in the study, with a specific focus on the Pavlovian bias parameter(s). A Benjamini–Hochberg correction was applied to the threshold for statistical significance (i.e., the α-level).

## Results

### Intertemporal Go/No-Go task

We replicated all go/no-go task effects reported in Burghoorn et al. (2024). Below, we report the results in more detail.

#### General task performance

**Fig. 3:**
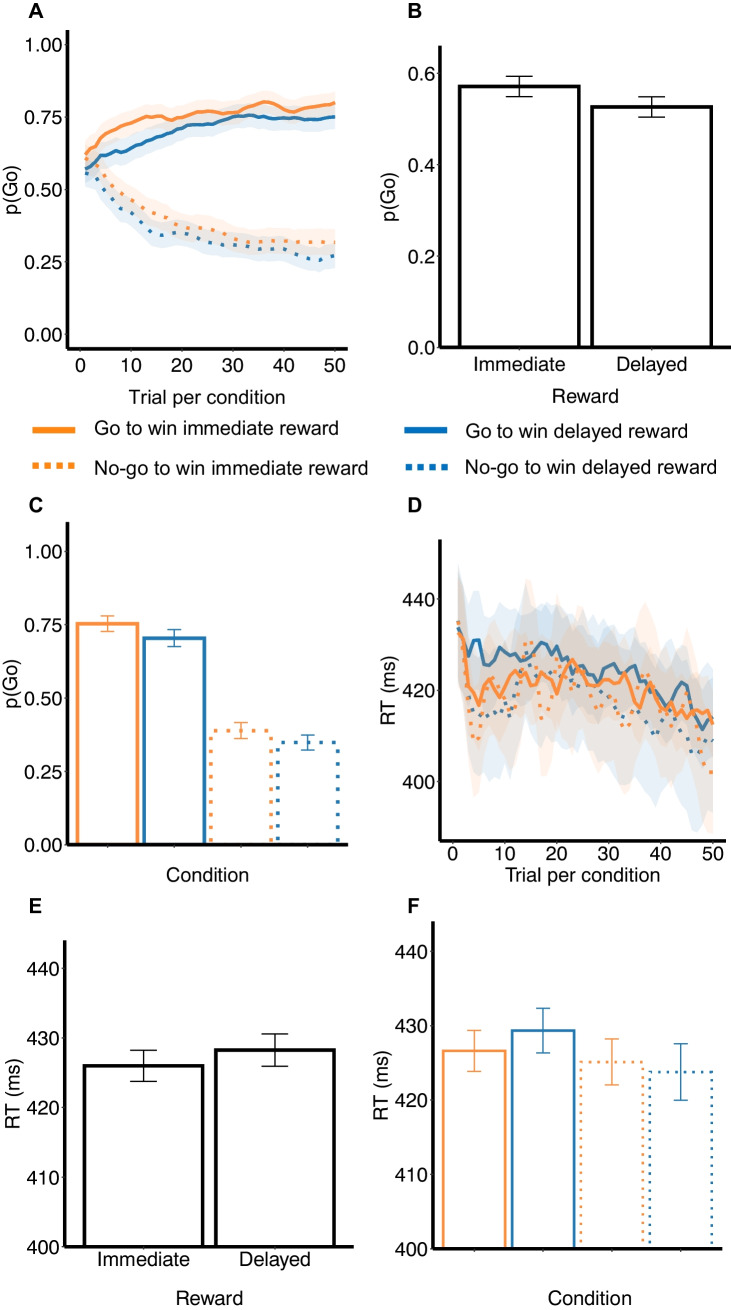
Go/No-Go task performance. *Note.* Results of the go/no-go task. **A**: Average trial-by-trial probability of making a go response (with 95% Confidence Intervals, CIs) per condition. **B-C:** Average proportion of aggregated go responses (with 95% CIs) per available reward, aggregated over go and no-go trials (B), and for go and no-go trials separately (C). **D:** Average trial-by-trial response times (RTs, in milliseconds, with 95% CIs) per condition. **E–F**: Average aggregated RTs (with 95% CIs) per available reward, aggregated over go and no-go trials (E), and for go and no-go trials separately (F)

Participants on average showed satisfactory task performance, making significantly more go responses in go trials than in no-go trials (*b*_GovsGrandMean_ = 1.3, 95% HDI [1.17, 1.43]). They did show a general go bias, because they were more likely to make go responses than no-go responses (*M*_*p*(Go)_ = 0.58, 95% HDI [0.55, 0.61]). Figure 3A suggests performance to gradually increase and then asymptote at a relatively stable level. A statistically significant interaction between the required action and the task block confirms that performance improved throughout the task (*b*_GovsGrandmean*Block_ = 0.52, 95% HDI [0.46, 0.58]), with participants making increasingly more go responses in go trials (*b*_Block_ = 0.46, 95% HDI [0.38, 0.54]) and fewer go responses in no-go trials (*b*_Block_ = − 0.58, 95% HDI [− 0.67, − 0.51]). The performance asymptotes may reflect the probabilistic feedback participants received (i.e., an 80% probability of correct feedback), a phenomenon termed probability matching (Herrnstein, 1961; Vulkan, 2000). As reported in detail in S3, individual differences in task performance did not moderate the intertemporal Pavlovian bias effect.

#### Intertemporal Pavlovian bias

##### Response bias

Replicating the intertemporal Pavlovian bias, participants made more go responses in immediate reward trials than in delayed reward trials (*b*_ImmvsGrandMean_ = 0.19, 95% HDI [0.07, 0.32]; Figs. 3A-C). This bias was not significantly different in go versus no-go trials (*b*_ImmvsGrandMean*GovsGrandMean_ = 0.01, 95% HDI [− 0.04, 0.07]; Figs. 3A and C). The availability of immediate rewards increased go responses on both go trials (enhancing performance; *b*_ImmvsGrandMean_ = 0.21, 95% HDI [0.06, 0.36]) and no-go trials (impairing performance; *b*_ImmvsGrandMean_ = 0.18, 95% HDI [0.11, 0.62]). The intertemporal Pavlovian bias did not change over the course of the task (*b*_ImmvsGrandMean*Block_ = 0.003, 95% HDI [− 0.04, 0.04]). We also did not observe a three-way interaction between reward, required action, and task block (*b*_ImmvsGrandMean*GovsGrandMean*Block_ = − 0.02, 95% HDI [− 0.05, 0.01]). In line with Burghoorn et al. (2024), we did observe substantial heterogeneity in the intertemporal Pavlovian bias effect, with the largest group of participants showing the expected effect, but also a considerable number of participants showing no effect or the opposite effect. We discuss these interindividual differences in detail in S4.

##### RT bias

There was no statistically significant main effect of the reward (immediate/delayed) on response times (*b*_ImmvsGrandMean_ = − 0.001, 95% HDI [− 0.004, 0.002]; Figs. 3D-F). However, there was a statistically significant interaction between the reward and required action (*b*_GovsGrandMean*ImmvsGrandMean_ = − 0.003, 95% HDI [− 0.01, − 0.0001]; Figs. 3D and F), such that only on go trials (i.e., for *correct* go responses), responses were faster in anticipation of immediate versus delayed rewards (go trials: *b*_ImmvsGrandMean_ = − 0.004, 95% HDI [− 0.01, − 0.0001]; no-go trials: *b*_ImmvsGrandMean_ = 0.002, 95% HDI [− 0.003, 0.01]). The observed inter-individual variability in this effect is discussed in S4. We also observed a main effect of required action (*b*_GovsGrandMean_ = 0.01, 95% HDI [0.005, 0.01]), with faster incorrect go responses (go responses made on no-go trials, i.e., false alarms) than correct go responses (go responses made on go trials). The faster false alarms may hence be viewed as impulsive errors and may point towards a speed-accuracy trade-off (also discussed in S3). Finally, a main effect of task block showed that responses generally became faster over the course of the task (*b*_Block_ = − 0.005, 95% HDI [− 0.01, − 0.001]). This effect did not interact with the effects of reward (*b*_ImmvsGrandMean*Block_ = 0.001, 95% HDI [− 0.001, 0.003]) or required action (*b*_ImmvsGrandMean*GovsGrandMean*Block_ = 0.001, 95% HDI [− 0.001, 0.003]).

##### Summary

We replicated the intertemporal Pavlovian bias on the probability of go responses (i.e., an intertemporal Pavlovian *response* bias), with increased go responding in anticipation of immediate (versus delayed) rewards. The magnitude of this effect (*b* = 0.19) was highly similar to Burghoorn et al., (2024; *b* = 0.17). In contrast to this previous study, we additionally observed an intertemporal Pavlovian bias on the speed or vigour of go responses (i.e., an intertemporal Pavlovian *RT* bias), with faster correct go responses in anticipation of immediate (versus delayed) rewards.

#### Intertemporal Pavlovian bias and mental health

##### Response bias

Contrary to our hypotheses, the intertemporal Pavlovian response bias was not associated with any of the mental health variables assessed in the present study. More specifically, we did not observe any statistically significant two-way interactions between the effect of reward (immediate/delayed) and the mental health variables on go responding, nor any higher-order interactions involving these variables (both with and without correcting for multiple comparisons). The results, including a visualization of the nonsignificant interaction patterns, are detailed in S5.

Several studies on Pavlovian biases (some of which also examined associations with mental health) have taken a complementary or alternative approach by comparing aggregated performance in the different go/no-go conditions or trial types (Albrecht et al., 2016; Cavanagh et al., 2013; Mkrtchian et al., 2017; Montagnese et al., 2020; Moutoussis et al., 2018). To avoid overlooking possibly relevant patterns, we performed additional, non-preregistered analyses using this approach (see S6 for details). Although we did observe mental health symptoms to be (mostly negatively) associated with performance in the different go/no-go conditions, these more likely reflected general performance advantages or benefits, instead of a stronger or weaker intertemporal Pavlovian bias; we return to the implications of this interpretation below.

##### RT bias

**Fig. 4:**
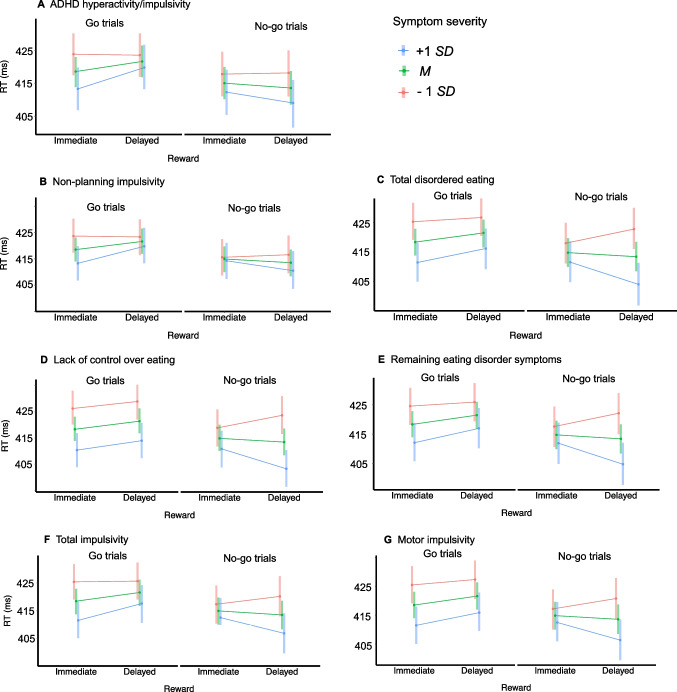
Intertemporal Pavlovian RT bias and mental health. *Note.* Statistically significant associations between the intertemporal Pavlovian RT bias and mental health variables. Panels display the three-way interactions between the available reward (immediate / delayed), the required action (go / no-go), and the mental health symptom severity (at the mean symptom level [*M*], and 1 standard deviation above [+ 1 *SD*] and below [−1 *SD*] the mean). The panels display model-based estimated marginal means and 95% HDIs, which were back-transformed from the logarithmic scale to the response scale (ms) to facilitate interpretation. As a result of the involved non-linear (back-)transformations, the means presented here may deviate from the raw RT means and 95% CIs presented in Fig. 3. **A-B:** An increased intertemporal Pavlovian RT bias on go trials (with faster correct responses in anticipation of immediate versus delayed rewards) was associated with ADHD hyperactivity/impulsivity (A) and non-planning impulsivity (B). **C-G:** A *reversed* intertemporal Pavlovian RT bias on no-go trials (with faster incorrect responses in anticipation of delayed versus immediate rewards) was associated with eating disorder symptoms (all three subscores, C-E), total impulsivity (F), and motor impulsivity (G). Estimates of all post-hoc tests are reported in S5. These also include the estimates and figures of the non-significant interactions between the intertemporal Pavlovian biases and the mental health variables

We observed statistically significant associations between the intertemporal Pavlovian RT bias and ADHD hyperactivity/impulsivity symptoms, eating disorder symptoms, and impulsivity. While the two-way interactions between the effect of reward and these mental health variables were not statistically significant, there were several statistically significant three-way interactions between reward, required action, and the mental health variables (Fig. 4A-G). Specifically, we observed that a stronger RT bias on *go* trials (i.e., faster correct go responses in anticipation of immediate versus delayed rewards) was associated with ADHD hyperactivity/impulsivity symptoms (*b* = − 0.001, 95% HDI [− 0.001, − 0.0001], Fig. 4A) and with nonplanning impulsivity (*b* = − 0.001, 95% HDI [− 0.002, − 0.0001], Fig. 4B). A stronger *reversed* RT bias on *no-go* trials (i.e., faster false alarms for delayed versus immediate rewards) was associated with eating disorder symptoms (total disordered eating: *b* = 0.001, 95% HDI [0.0004, 0.002], Fig. 4C; lack of control over eating: *b* = 0.006, 95% HDI [0.002, 0.01], Fig. 4D; remaining eating disorder symptoms: *b* = 0.001, 95% HDI [0.0004, 0.002], Fig. 4E), as well as with total impulsivity (*b* = 0.001, 95% HDI [0.00004, 0.001], Fig. 4F) and motor impulsivity (*b* = 0.001, 95% HDI [0.0003, 0.003], Fig. 4G). Detailed results, including estimates and visualizations for all significant and nonsignificant interactions, and for the post-hoc tests (if applicable), can be found in S5.

#### Intertemporal impatience and mental health

The bivariate associations between the mental health variables, and between the mental health variables and intertemporal impatience, are displayed in S7. We did not observe any statistically significant associations between intertemporal impatience and any of the mental health variables. Although the uncorrected Pearson correlations suggested impatience to be weakly associated with increased motor impulsivity (*r* = 0.13, *p* = 0.011) and total impulsivity (*r* = 0.10, *p* = 0.047), these correlations did not survive corrections for multiple testing.

#### Reinforcement learning models

**Fig. 5:**
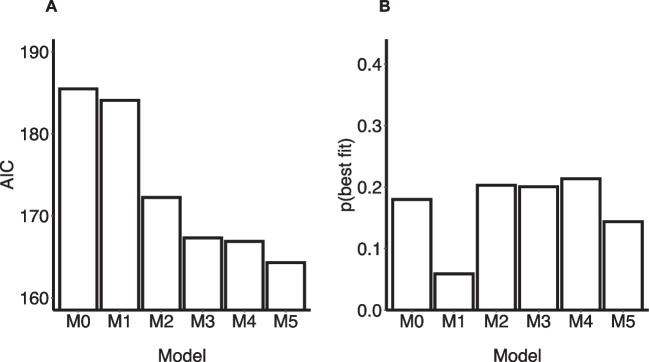
RL model fit. *Note.* Model fits of the six reinforcement learning (RL) models that were fitted to the go/no-go task data. **A:** Median Akaike’s Information Criterion (AIC) value across participants. **B:** Model frequency, displayed as the proportion of participants for which each model had the lowest AIC value

Figures 5A-B display the model fits of the six RL models fitted to the go/no-go data. Model comparison using the median AIC showed the increasingly complex models to improve model fit in a stepwise manner. The median AIC values for M3 (167.34), M4 (166.87), and M5 (164.29) were close, with a small advantage for the model incorporating both a cue-response bias and a learning bias (M5), in line with Burghoorn et al. (2024) and Swart et al., (2017, 2018). The model frequency index did not show a clear winner, with models M0 and M2-M4 all having an approximately equal proportion of participants for whom this model formed the best fit.^2^ Importantly, however, only M3 was successfully validated on all three validation criteria, showing successful parameter recovery, model recovery, and posterior predictive performance (described in detail in S9). This confirms validation results reported previously (Burghoorn et al., 2024). Given the importance of model validation, especially in light of our goal to test for the associations between the model parameters and mental health, we selected M3 as the winning model.^3^ A summary of parameter estimates of M3 can be found in Table 2. A one-sample *t*-test and a one-sample Wilcoxon signed-rank test showed that the Pavlovian cue-response bias parameter was statistically significantly different from zero (one-sample *t*-test: *t*(388) = 3.19, *p* = 0.002; one-sample Wilcoxon signed-rank test: *V* = 44,682, *p* < 0.001).

**Table 2:** Summary parameter estimates M3

Model parameter	*M*	*Mdn*	95% CI
α	0.21	0.13	[0.19, 0.23]
τ	7.33	7.25	[7.05, 7.62]
ξ	0.17	0.06	[0.15, 0.20]
*b*	0.09	0.05	[0.06, 0.12]
π	0.05	0.02	[0.02, 0.08]

Summary of the parameter estimates of the winning reinforcement learning model, M3. *M* = mean, *Mdn* = median, 95% CI = 95% Confidence Interval

##### RL models and mental health

We observed no statistically significant associations between the Pavlovian cue-response bias parameter π and any of the mental health variables assessed in this study. We did observe statistically significant positive associations between the irreducible noise parameter ξ and alcohol problems (*r* = 0.11, *p* = 0.031) and motor impulsivity (*r* = 0.13, *p* = 0.013), but these associations did not survive a correction for multiple testing. An overview of all correlations can be found in S10.

#### Secondary results

##### Choice titration

The immediate reward derived from the first choice titration, and preference-matched to the delayed reward of €28 in 120 days, varied across participants between €1 and €28 (raw *M* = 14.4, *SD* = 7.98). As reported in detail in S11, these individual difference in intertemporal impatience did not moderate the intertemporal Pavlovian bias effect. This is consistent with the idea that by using individualized preference-matched reward pairs in the go/no-go task, we were able to test for the intertemporal Pavlovian bias effect beyond individual differences in subjectively discounted reward value (as expressed by choice preferences).

The immediate rewards derived from the second choice titration again ranged between €1 and €28 (raw *M* = 15.35, *SD* = 7.84). In line with previous work (Burghoorn et al., 2024), participants became slightly more patient from the first to the second choice titration, reflected by a significantly higher immediate reward amount in the second choice titration (*b*_PostvsGrandMean_ = 0.48, 95% HDI [0.32, 0.63]). This small drift in the level of impatience could mean that the reward pairs used in the go/no-go task did not remain preference-matched throughout the task. However, the increase in *patience* would imply that the delayed reward became more valuable, which should then have resulted in increased go responding towards *delayed* rewards—contrary to what we observed. Thus, we consider it unlikely that the drift in impatience drove the observed intertemporal Pavlovian bias effect. As detailed in S12, we did observe the *per-participant* drift in impatience to moderate the intertemporal Pavlovian bias, such that the bias was stronger as a function of the increase in impatience (possibly through an increase in the subjective value of the immediate reward used in the go/no-go task). We do note that the majority of participants either did not show a drift in impatience (57% of participants), or became slightly more *patient* (33% of participants), and that those individuals *also* showed a statistically significant intertemporal Pavlovian bias effect. This, again, does not support the possibility that the intertemporal Pavlovian bias was driven by a drift in intertemporal impatience.

##### Reward valuation

Consistent with Burghoorn et al. (2024), in the *rating* version of the reward valuation task, participants on average rated the immediate reward higher than the delayed reward (see S13 for details). Thus, although the two rewards were matched based on revealed choice preferences, they were valued differently when rated individually. However, different from our previous study, the rating difference did not moderate the intertemporal Pavlovian bias, suggesting that the valuation difference between the two rewards did not strongly drive the intertemporal Pavlovian bias effect.

In the *ranking* version of the task (which we did not administer in our previous study), the probability of both rewards being ranked as equally attractive was statistically significantly lower than the probability of the rewards being ranked differently, again suggesting the two rewards were not valued as equally attractive when evaluated outside a choice context. Importantly, however, the probability of the immediate reward being ranked as more attractive than the delayed reward was not significantly different from the probability of the delayed reward being ranked as more attractive than the immediate reward. These results were independent of whether the task was administered before or after the go/no-go task. Thus, different from the rating task, the ranking task did not point towards a systematically higher valuation of either the immediate or delayed reward, the latter of which is consistent with the ranking results reported by Luo et al. (2009). Finally, consistent with the rating task, individual differences in the ranking scores did not moderate the intertemporal Pavlovian bias effect.

##### Cue ratings

We observed several statistically significant differences in subjective valuation of the cues; all pairwise differences are reported in S14. By randomly assigning the cues to go/no-go conditions, we prevented these differences from confounding the intertemporal Pavlovian bias effect. Cues were also rated slightly, but significantly higher after the go/no-go task than before the go/no-go task, but this did not interact with the effect of cue identity.

## Discussion

The goals of the present study were to replicate the intertemporal Pavlovian bias effect, and to examine in a general population sample whether the strength of this bias was associated with mental health symptoms that have previously been associated with increased or decreased intertemporal impatience, including symptoms of alcohol use disorder, nicotine dependence, depression, trait anxiety, ADHD, disordered eating, BMI, and impulsivity. As hypothesized, we replicated the intertemporal Pavlovian bias effect on go responding, with an increase in go responding in anticipation of immediate rewards compared to the anticipation of preference-matched delayed rewards, thus facilitating instrumental approach but impairing instrumental inhibition. The magnitude of this effect was highly similar to Burghoorn et al. (2024), supporting the reliability of this effect. We additionally observed an intertemporal Pavlovian bias on response times (RTs), with faster correct go responses in anticipation of immediate compared to delayed rewards. Contrary to our expectations, none of the mental health variables assessed in the present study were associated with the intertemporal Pavlovian bias on go responding (i.e., the intertemporal Pavlovian response bias). However, we did observe several associations between mental health and the intertemporal Pavlovian RT bias. While an enhanced RT bias on go trials (i.e., faster correct go responses in anticipation of immediate rewards) was associated with ADHD hyperactivity/impulsivity symptoms and nonplanning impulsivity, a *reversed* RT bias on no-go trials (i.e., faster false alarms in anticipation of delayed rewards) was associated with eating disorder symptoms, total impulsivity, and motor impulsivity. Finally, in contrast with the existing literature, none of the mental health variables were associated with individual differences in the level of intertemporal impatience (as assessed during the first choice titration procedure).

### Intertemporal Pavlovian bias effects

The replication of the intertemporal Pavlovian bias on go responding supports the robustness of the effect that we observed previously, and is consistent with the theory that the anticipation of an immediate reward elicits a stronger Pavlovian approach response than the anticipation a delayed reward, even when the two rewards are matched based on subjectively discounted reward value (as revealed through choice preferences). This intertemporal Pavlovian bias may form a psychological mechanism that underlies the temptation posed by immediate rewards, possibly contributing to intertemporally impatient behaviour. For instance, a hampered ability to show instrumental inhibition in the face of immediate temptations (e.g., the “next episode” button) may go at the cost of long-term (e.g., educational or occupational) goals.

The additional finding of an intertemporal Pavlovian RT bias suggests that the anticipation of immediate rewards not only increases the *probability* that a go response is made, but also increases the *vigour* with which these responses are made. This RT bias aligns with findings by Luo et al. (2009), who observed faster responses on a Monetary Incentive Delay (MID) task in anticipation of immediate compared to preference-matched delayed rewards. Moreover, the results are consistent with *valence*-driven Pavlovian bias effects, in which response bias effects have generally been accompanied by RT bias effects (Algermissen et al., 2022; Algermissen & Den Ouden, 2023; Guitart-Masip et al., 2011, 2012a, 2012b; Scholz et al., 2022; Swart et al., 2017, 2018). In Burghoorn et al. (2024), however, we did not observe RT effects. Possibly, the present study’s increased statistical power, resulting from the larger sample size, contributed to a statistically significant RT effect—although it should be noted that the direction of the non-significant RT patterns in our previous work did not point towards any RT effects. The specificity of the intertemporal Pavlovian RT bias to *correct* go responses illustrates that Pavlovian biases are not always maladaptive to instrumental goals but can also be adaptive (as also discussed by Dayan et al., 2006).

Fitting the reinforcement learning models to the go/no-go responses showed similar model evidence for the cue-response bias, learning bias, and the model incorporating both biases. However, in line with Burghoorn et al. (2024), only the cue-response bias was successfully validated, showing consistently satisfactory parameter recovery, model recovery, and posterior predictive performance, and was therefore selected as winning model. This cue-response bias model posits that cues signalling immediate rewards elicit a stronger general behavioural activation compared to delayed rewards, increasing the action weight of go responses, and that the per-participant strength of this effect is scaled by the cue-response bias parameter π. Our finding that this cue-response bias accurately fits and predicts the observed go/no-go data is in line with a large body of valence-driven Pavlovian bias showing support for this model (Guitart-Masip et al., 2014) and suggests that similar computational mechanisms apply to Pavlovian bias effects driven by reward timing.

The immediate and delayed reward used in the go/no-go task were preference-matched using a choice titration procedure, hereby aiming to test for an effect of reward delay beyond individual differences in subjectively discounted reward value. Using two different versions of a reward valuation task (i.e., a rating and ranking version), we examined whether the rewards would also be valued similarly when evaluated outside a choice context. While the immediate reward was, on average, *rated* higher than the delayed reward, the immediate reward was not *ranked* higher than the delayed reward (nor was the delayed reward ranked higher than the immediate reward). These results are consistent with a discrepancy in findings between our previous study, in which we observed the immediate reward to be *rated* higher than the delayed reward, versus those by Luo et al. (2009), who used a near-identical choice titration procedure to derive preference-matched rewards but did not observe a statistically significant difference in *ranking* of the rewards. This relates to a large body of literature on the role of different preference elicitation methods (e.g., choices, ratings, rankings) in determining the subjective value of rewards (Bettman et al., 1998; Johnson & Busemeyer, 2005; Kvam & Busemeyer, 2020; Lichtenstein & Slovic, 1971; Slovic, 1995; Tversky et al., 1990; Warren et al., 2011) and complicates a decision on when two rewards are truly preference-matched. We conclude that the intertemporal Pavlovian bias reported here is restricted to reward pairs that were preference-matched through choices. In contrast to Burghoorn et al. (2024) and Luo et al. (2009), the difference in rankings or ratings between the immediate and delayed reward did not moderate the intertemporal Pavlovian bias effect. This alleviates the concern that these valuation differences formed the primary mechanism underlying the intertemporal Pavlovian bias. We do acknowledge, however, that the nonsignificant moderation patterns were similar to what we observed in Burghoorn et al. (2024), suggesting that valuation differences may to some extent contribute to the Pavlovian bias effect. We refer to this previous study for an extensive discussion on the implications of this possibility.

### Response Bias and mental health

In contrast to our hypotheses, the intertemporal Pavlovian response bias was not associated with any of the mental health variables, and none of the parameters of our winning RL model were associated with the mental health variables. We did observe statistically significant associations between mental health and aggregated performance accuracy in each of the four go/no-go conditions. However, these more likely reflected general performance deficits or advantages, instead of an enhanced or reduced intertemporal Pavlovian bias. This also implies that previously reported associations between Pavlovian biases and mental health that were *solely* based on aggregated performance in each of the four conditions (e.g., the clinical group comparisons by Montagnese et al., 2020) may have been confounded by such general performance deficits or advantages. We recommend future research to disentangle general performance deficits or advantages from the hypothesized Pavlovian biases.

The absence of statistically significant associations between the intertemporal Pavlovian response bias and mental health in the present study suggests that the effect of the anticipated reward timing on instrumental approach/withdrawal decisions does not explain individual differences in the mental health problems assessed in this study, despite the fact that these mental health problems have previously been characterized by increased or decreased impatience. Future research may therefore shift the focus to alternative mechanisms that could contribute to these individual differences in behaviour and mental health, such as individual differences in general self-control (Figner et al., 2010), time perception (Kim & Zauberman, 2019), or reward valuation (Scheres et al., 2013).

Several alternative explanations for the absence of associations between the intertemporal Pavlovian response bias and mental health should be discussed. First, contrary to our expectations, we also did not observe statistically significant associations between individual differences in intertemporal impatience (as assessed using the choice titration) and the mental health symptoms. With the exception of anxiety, this contrasts with a large body of literature reporting robust associations between intertemporal impatience and the mental health variables assessed in the present study. While the majority of these studies compared individuals with a clinical diagnosis with individuals without a diagnosis (Amlung et al., 2016, 2019; Jackson & MacKillop, 2016; MacKillop et al., 2011; Marx et al., 2021; Patros et al., 2016; Pauli-Pott & Becker, 2015), continuous associations have also been observed in general population samples (Amlung et al., 2016, 2017; Burghoorn et al., 2025; Keidel et al., 2024; Levin et al., 2018; Levitt et al., 2022). These continuous associations have been small in magnitude, however, with Pearson correlations ranging between 0.09 and 0.17. The nonsignificant correlations observed in the present study were at the lower end or even below this range, suggesting that any possibly existing associations may have been too small to be statistically significant in our sample. Given our aim to study the role of the intertemporal Pavlovian bias in mental health problems characterized by increased or decreased impatience, our results warrant replication in a sample in which the severity of mental health problems is indeed associated with increased or decreased impatience. One point that should be considered is that the severity of mental health problems in general population samples is generally skewed towards the lower end, supported by comparable mental health scores in our sample and in that used by Levitt et al. (2022). This reduced interindividual variation in mental health scores may have reduced the probability of finding statistically significant associations between mental health scores and the intertemporal Pavlovian bias. Future research could try to overcome this limitation by oversampling participants with higher symptom scores. Although such samples may not be as representative of the general population, they may increase statistical power for detecting associations between the intertemporal Pavlovian bias and mental health. Serving as an initial indication, and following a reviewer’s suggestion, exploratory analyses in subsamples of individuals with symptom scores above the 75th percentile tentatively pointed towards possible associations between the intertemporal Pavlovian biases and alcohol use disorder scores, BMI, lack of control over eating, anxiety, and ADHD inattention scores, although not all of these associations reached statistical significance (see S15 for details). We encourage future studies to study these associations in larger samples of at-risk or high-symptom individuals.

A second possible explanation for the absence of associations between the intertemporal Pavlovian response bias and mental health is that we only examined Pavlovian responses elicited by the anticipation of *monetary* immediate and delayed rewards. Despite observing an intertemporal Pavlovian bias with such rewards in the present study and in Burghoorn et al. (2024), it is possible that its association with mental health problems may be stronger for *disorder-specific* outcomes, because cues signalling such outcomes may acquire more incentive salience compared with monetary cues (Godier & Park, 2014). Examples of disorder-specific outcomes include food (for obesity or disordered eating) or substances (for substance use disorders). Research with food or substance outcomes has been conducted for valence-driven Pavlovian biases (see Garbusow et al., 2022 for a review, and see Belanger et al., 2022, for a comparison between monetary and gustatory outcomes). However, to the best of our knowledge, no research has directly compared the associations between the strength of Pavlovian biases and mental health with monetary versus disorder-specific outcomes. Illustrating the possible role of outcome specificity, some studies reported mood and anxiety symptoms to be associated with individual differences in Pavlovian responses elicited by the anticipation of punishments, but not with individual differences in Pavlovian responses elicited by the anticipation of rewards (Mkrtchian et al., 2017; Nord et al., 2018; Peterburs et al., 2022). In the context of intertemporal outcomes, anxiety symptoms may be more strongly driven by a particularly strong avoidance of immediate punishments (e.g., social disapproval, or feelings of distress) instead of a particularly strong approach towards immediate rewards.^4^ The use of disorder-specific instead of general monetary outcomes does limit the range of mental health problems that can be assessed with the same task. Using different tasks and outcomes for each mental health category complicates a comparison of the associations between the intertemporal Pavlovian bias and mental health across the different mental health categories. This contrasts with recent reviews calling for *less* heterogeneity in tasks and outcomes to study the role of Pavlovian biases as possible *transdiagnostic* mechanism (Doñamayor et al., 2021; Garbusow et al., 2022). It is for this reason that we investigated an intertemporal Pavlovian bias elicited by the anticipation of universally rewarding monetary outcomes across a range of mental health problems. Nevertheless, gaining more insight into the role of outcome-specificity would be highly relevant for understanding the mental health implications of the intertemporal Pavlovian bias.

Finally, research suggests that cognitive tasks that consist of a large number of repeated trials with predictable structure, may induce a shift from the use of target decision processes (i.e., reinforcement learning) to task-specific decision processes, such as a process-of-elimination strategy (i.e., learning what response to make for a subset of stimuli and deducing from that what response to make for the remaining stimuli). The use of such task-specific strategies has been proposed to reduce external task validity by creating a mismatch between the decision processes that are used in the task versus in real-world situations (Li et al., 2022). Moreover, the use of task-specific strategies has been argued to reduce task reliability by reducing between-participant variability in performance (i.e., inducing ceiling effects), thereby limiting associations with other variables (e.g., mental health; Zorowitz et al., 2023). As detailed in S16, however, the intertemporal Pavlovian response bias, as well as the RL model parameters, showed excellent reliability. Moreover, exclusively examining the association between the intertemporal Pavlovian bias and mental health in the first go/no-go task block, during which the reliance on task-specific processes can be assumed to be minimal, resulted in the same conclusions (i.e., the intertemporal Pavlovian biases and their associations with mental health) as for the full task. Thus, we consider it unlikely that this explained the absence of associations between the intertemporal Pavlovian response bias and mental health.

### RT bias effects and mental health

In contrast to the absence of an association between the intertemporal Pavlovian *response* bias and mental health, we did observe the intertemporal Pavlovian *RT* bias to be associated with several mental health variables. We note here that we did not have preregistered hypotheses on the RT bias and its mental health associations, following the absence of an RT bias in Burghoorn et al. (2024). Therefore, we encourage future research to replicate these findings through preregistered studies. In the present study, ADHD hyperactivity/impulsivity symptoms and non-planning impulsivity were associated with a stronger RT bias in go trials, i.e., more vigorous correct go responses in anticipation of immediate rewards. The specificity of the association between the RT bias and ADHD symptoms to hyperactivity/impulsivity symptoms (instead of inattention symptoms or total ADHD symptoms) is consistent with the increased behavioural activation that is central to both the intertemporal Pavlovian bias and the hyperactivity symptoms, and with the conceptual and previously reported empirical links between impulsivity and intertemporal impatience (Baumann & Odum, 2012; Jauregi et al., 2018; Keidel et al., 2024; Malesza & Kalinowski, 2021; Malesza & Ostaszewski, 2016; Zhou et al., 2021). It also aligns with previous research showing hyperactivity/impulsivity symptoms to be more strongly characterized by impatient choices than inattention symptoms (Scheres et al., 2008). Scheres et al. attributed this to a possibly increased aversion towards delays; the present study suggests that a stronger Pavlovian approach response elicited by immediate rewards may form an alternative or complementary mechanism.

The association between non-planning impulsivity and the intertemporal Pavlovian bias also supports the above-described link between impulsivity and intertemporal impatience. Although we had hypothesized to see a more prominent association between the intertemporal Pavlovian bias and *motor* impulsivity (following the Pavlovian bias as conditioned motor response), the association between the intertemporal Pavlovian bias in RTs with nonplanning impulsivity supports the definition of non-planning impulsivity as a “lack of futuring” or a strong present orientation (Patton et al., 1995). For both ADHD hyperactivity/impulsivity and nonplanning impulsivity, it should be noted that the enhanced response invigoration in anticipation of immediate rewards only occurred for *correct* go responses and was thus adaptive. This finding aligns with a recent shift from a deficit-focused view of ADHD symptoms to a view that also incorporates possible strengths (Schippers et al., 2024), as well as with studies showing that intertemporal impatience may be adaptive in some situations (Fenneman et al., 2022).

Unexpectedly, in no-go trials, motor impulsivity and total impulsivity were associated with a *reversed* intertemporal Pavlovian RT bias. The more vigorous false alarms in anticipation of *delayed* instead of immediate rewards contrast with the conceptual and empirical links between intertemporal impatience and impulsivity discussed above, which would suggest more vigorous false alarms in anticipation of *immediate* rewards. Somewhat less unexpected was the observation of a reversed RT bias for individuals with increased eating disorder symptoms. Eating disorder symptoms, particularly those of the restrictive type, have been associated with a preference for delayed over immediate rewards (Amlung et al., 2019), and with increased instrumental approach responding towards low-calorie versus high-calorie rewards (Vogel et al., 2020). This aligns with the more vigorous go responses in anticipation of delayed versus immediate rewards observed in the present study, although we cannot readily explain why this occurred only for incorrect go responses. The results could also be interpreted as more careful or deliberate responding in anticipation of immediate rewards when active responses are instrumentally maladaptive, which would point towards more hesitative decision-making towards immediate rewards (e.g., more hesitative responses towards immediate snack consumption). A similar explanation was proposed by Scholz et al. (2020), who observed mood-anxiety symptoms to be associated with improved performance on no-go trials. However, a visual inspection of the association between the RT bias and eating disorder symptoms suggests that the difference between individuals with low, average, and high symptoms is most apparent in their response towards delayed rewards, supporting the former explanation. Future research attempting to replicate these findings could include baseline trials without rewards, allowing one to further disentangle whether any effects are driven by the anticipation of immediate rewards, delayed rewards, or both.

We did not hypothesize the subset of symptoms reflecting a lack-of-control overeating to show a reversed Pavlovian bias, because eating disorders characterized by such symptoms (i.e., bulimia nervosa and binge-eating disorder) have been associated with an increased preference for immediate rewards (Amlung et al., 2019). Although a reversed Pavlovian bias in no-go trials may be shared among all eating disorder symptoms (as suggested by our data), the assessment of the different eating disorder symptoms with the same instrument, and the limited number of items assessing a lack of control (2/12 items) may have also contributed to a correlation between the two subscales and to a difficulty in detecting differential associations.

Finally, we note that the reliability of the intertemporal Pavlovian RT bias was relatively low, particularly in the no-go trials (see S16 for details). The discrepancy in reliability between the intertemporal Pavlovian response and RT bias may result from the reduced number of trials on which the RT bias was based, and/or from a generally noisier measurement of RTs compared to responses—particularly for no-go trials, which were exclusively based on incorrect go responses. The reduced reliability warrants a careful interpretation of the results and calls for replication of the RT effects.

### Strengths, limitations, and future directions

The present study carries several strengths. First, our study goals, hypotheses, design, analyses, and sample size were preregistered, and all study materials, data, and analysis scripts are openly available. Second, we used a sample size with adequate statistical power to replicate the intertemporal Pavlovian bias effect. Moreover, our sample was considerably larger than most existing studies on the association between Pavlovian biases and mental health symptoms, with the exception of Moutoussis et al., (2018; *N* = 785; no statistically significant associations), Montagnese et al., (2020; same dataset as Moutoussis et al., 2018; no statistically significant associations), and Scholz et al., (2020, *N* = 500; reduced Pavlovian bias associated with mood-anxiety). The frequent occurrence of null or unexpected findings in these large-sample studies raises the question whether previously reported associations from small-sample studies would be replicated with larger samples (but also see Sebold et al., 2021 showing increased Pavlovian biases in alcohol use disorder patients compared to healthy controls with *N* = 452). We encourage future studies on the association between Pavlovian biases and mental health to use large samples, thereby reducing the chance of Type-I and Type-II errors. The present study shows that the magnitude of interactions between intertemporal Pavlovian biases and mental health symptoms may be small (i.e., we observed a maximum absolute effect size of 0.14), indicating that future studies aiming to detect such small interaction effects would benefit from using even larger samples than ours.

Third, by not only including total mental health scores in our analyses, but additionally examining subsets of symptoms (e.g., different ADHD symptom clusters), we were able to test for possible differential associations with the intertemporal Pavlovian bias. This is consistent with a heterogeneity in symptoms within mental health categories, and with the possibility that different symptom (clusters) may be driven by different underlying mechanisms (Cuthbert & Insel, 2013). Fourth, we assessed the reliability of the intertemporal Pavlovian bias effects, the RL model parameters, and mental health measures, and validated our RL models using parameter recovery, model recovery, and posterior predictive checks. Finally, we examined the valuation of the study rewards using three different measures (i.e., choices, ratings, and rankings), allowing us to consider the role of preference elicitation methods in our discussion.

At the same time, our study also carries limitations, which provide possible directions for future research. A first limitation was discussed before and involves the relatively restricted variation in mental health symptoms in our general population sample. Second, although we examined the computational mechanisms that may underlie the intertemporal Pavlovian response bias, we did not do this for the intertemporal Pavlovian RT bias. Response times of decision tasks have traditionally been modelled using sequential sampling models, such as drift–diffusion models (DDMs). Such models typically do not include the learning component that is essential for reinforcement learning data. More recently, however, RL and DDMs models have been integrated into a new type of model that uses a joint distribution of responses and RTs (i.e., RLDDMs; Fontanesi et al., 2019; Millner et al., 2018). While applying such models was beyond the scope of the present study, we encourage future research to examine which computational mechanisms might underlie the reported associations between the intertemporal Pavlovian bias on RTs and mental health. A relevant priority might be to first increase the reliability of the intertemporal Pavlovian RT bias. Future research could consider increasing the number of trials to increase the reliability, although, as described earlier, an overly long task may also adversely influence reliability and external validity. Adverse side-effects of increasing the number of trials might be avoided by increasing the task difficulty (which would also have the advantage of resulting in more observations for incorrect go responses) or by taking other measures to avoid practice effects (see Zorowitz et al., 2023 for an example).

Third and finally, the cross-sectional nature of the present study prevents us from drawing conclusions regarding the causal direction of the associations tested here, i.e., whether an increased or decreased intertemporal Pavlovian bias forms a psychological mechanism contributing to mental health problems, as we theorized, or whether such mental health problems result in increased or reduced intertemporal Pavlovian bias effects, or both. For instance, while an enhanced response invigoration in anticipation of delayed versus immediate rewards may contribute to disordered eating patterns, it may also be a consequence of these disordered eating patterns (in line with research suggesting that a decreased intertemporal impatience in individuals with anorexia nervosa may be a consequence of the disorder; Decker et al., 2015). We encourage future research to examine these causal directions by adopting longitudinal study designs, which allow one to study whether individual differences in intertemporal Pavlovian biases predict changes in mental health symptoms (see Garbusow et al., 2016; Geurts, Van den Heuvel et al., 2022; Huys et al., 2016 for examples on valence-based Pavlovian biases), and/or intervention studies, which allow one to study whether experimentally altering intertemporal Pavlovian biases influences mental health symptoms.

### Conclusions

In the present study, we replicated the intertemporal Pavlovian bias on instrumental actions reported in Burghoorn et al. (2024), showing that the anticipation of immediate rewards enhanced instrumental approach but impaired instrumental inhibition. Our RL models again point towards the role of a Pavlovian cue-response bias a computational mechanism underlying this effect. Moreover, we provided evidence of an additional intertemporal Pavlovian bias effect on the speed or vigour of approach responses, with faster correct go responses in anticipation of immediate than delayed rewards. The intertemporal Pavlovian response bias was not associated with mental health symptoms, suggesting that it may not be implicated in individual differences in these mental health symptoms. However, an increased intertemporal Pavlovian RT bias on go trials, reflecting more vigorous correct go responses in anticipation of immediate rewards, was associated with ADHD hyperactivity/impulsivity and non-planning impulsivity symptoms. A reversed intertemporal Pavlovian RT bias on no-go trials, reflecting more vigorous false alarms in anticipation of delayed rewards, was associated with eating disorder symptoms, total impulsivity, and motor impulsivity. These results point towards the relevance of the intertemporal Pavlovian RT bias for these mental health symptoms.

## Supplementary Information

Below is the link to the electronic supplementary material.

Supplementary file1 (DOCX 4.75 MB)

## Data Availability

All data and materials are available on the Open Science Framework: https://osf.io/p5bz9/.

## Footnotes

1 The question to what extent individuals with anorexia nervosa consider food as rewarding has been debated (Steinglass et al., 2012). Therefore, weaker or even reversed Pavlovian biases in anticipation of immediate food outcomes may also result from a lower subjective value attributed to food (and/or a higher subjective value attributed to long-term health outcomes), instead of or in addition to increased control to overcome these biases. The use of monetary outcomes in the present study allowed us to test for intertemporal Pavlovian biases while bypassing the complex reward properties of food outcomes.

2 Based on a question by a reviewer, we investigated the relatively strong model frequency evidence in support of the most basic model M0. Details are reported in S8. In sum, individuals for whom M0 fitted best showed highly similar AIC values (i.e., model fit) across all models, whereas individuals for whom M2-M5 fitted best showed a larger difference in model fit between the more complex models M2-M5 versus the more simple models M0-1. However, these quantitative differences between model fits are not captured by the more qualitative model frequency index, resulting in more model frequency support for M0. Second, individuals who showed high performance on the go/no-go task were more often fitted best by M0, possibly because M0 is the only model that does not include parameters (e.g., irreducible noise, go bias, or Pavlovian bias) that aim to capture biases in task performance.

3 Following a reviewer’s suggestion, we also computed Bayesian Information Criterion (BIC) values to examine whether the model fit and model recovery results would more closely align when using BIC instead of AIC values. Using BIC reduced M5’s slight advantage in model fit, resulting in median BIC values across M3-5 that were even closer together than the median AIC values (BIC: 183.8, 183.36, and 184.08, respectively). Consistent with the model recovery results using AIC, model recovery with BIC showed support for the successful validation of M3 but not M4 and M5. Thus, the model fit and model recovery results were indeed more closely aligned when using BIC than AIC, which likely arises from BIC’s tendency to penalise model complexity more strongly than AIC.

4 It should be noted that the immediate relief from distress or the absence of negative outcomes that often follows from avoidance behaviour may also be interpreted as immediate reward, i.e., as immediate negative reinforcer.

## Code availability

All code is available on OSF: https://osf.io/p5bz9/.

## Declarations

### Preprint

This manuscript has been deposited as preprint in the PsyArXiv Preprints repository: https://doi.org/10.31234/osf.io/h4a38_v1

### Open Practices Statement

The study was preregistered on OSF prior to conducting the research (https://osf.io/4qczy) and the preregistration adheres to the disclosure requirements of the institutional registry. All materials, analysis code, and data are also available on OSF (https://osf.io/p5bz9/).

## References

[CR1] Albrecht, M. A., Waltz, J. A., Cavanagh, J. F., Frank, M. J., & Gold, J. M. (2016). Reduction of Pavlovian bias in schizophrenia: Enhanced effects in clozapine-administered patients. *PLoS ONE,**11*(4), 1–23. 10.1371/journal.pone.0152781 10.1371/journal.pone.0152781 PMC4833478 27044008

[CR2] Algermissen, J., & Den Ouden, H. E. M. (2023). Goal-directed recruitment of Pavlovian biases through selective visual attention. *Journal of Experimental Psychology: General,**152*(10), 2941–2956. 10.1037/xge0001425 37199975 10.1037/xge0001425

[CR3] Algermissen, J., Swart, J. C., Scheeringa, R., Cools, R., & Den Ouden, H. E. M. (2022). Striatal BOLD and midfrontal theta power express motivation for action. *Cerebral Cortex,**32*(14), 2924–2942. 10.1093/cercor/bhab391 34849626 10.1093/cercor/bhab391 PMC9290551

[CR4] Amlung, M., Marsden, E., Holshausen, K., Morris, V., Patel, H., Vedelago, L., Naish, K. R., Reed, D. D., & McCabe, R. E. (2019). Delay discounting as a transdiagnostic process in psychiatric disorders: A meta-analysis. *JAMA Psychiatry,**76*(11), 1176–1186. 10.1001/jamapsychiatry.2019.2102 31461131 10.1001/jamapsychiatry.2019.2102 PMC6714026

[CR5] Amlung, M., Petker, T., Jackson, J., Balodis, I., & Mackillop, J. (2016). Steep discounting of delayed monetary and food rewards in obesity: A meta-analysis. *Psychological Medicine,**46*(11), 2423–2434. 10.1017/S0033291716000866 27299672 10.1017/S0033291716000866

[CR6] Amlung, M., Vedelago, L., Acker, J., Balodis, I., & MacKillop, J. (2017). Steep delay discounting and addictive behavior: A meta-analysis of continuous associations. *Addiction,**112*(1), 51–62. 10.1111/add.13535 27450931 10.1111/add.13535 PMC5148639

[CR7] Baumann, A. A., & Odum, A. L. (2012). Impulsivity, risk taking, and timing. *Behavioural Processes,**90*(3), 408–414. 10.1016/j.beproc.2012.04.005 22542458 10.1016/j.beproc.2012.04.005 PMC3897391

[CR8] Belanger, M. J., Chen, H., Hentschel, A., Garbusow, M., Ebrahimi, C., Knorr, F. G., Zech, H. G., Pilhatsch, M., Heinz, A., & Smolka, M. N. (2022). Development of novel tasks to assess outcome-specific and general Pavlovian-to-instrumental transfer in humans. *Neuropsychobiology,**81*(5), 370–386. 10.1159/000526774 36380640 10.1159/000526774

[CR9] Benjamini, Y., & Hochberg, Y. (1995). Controlling the false discovery rate: A practical and powerful approach to multiple testing. *Journal of the Royal Statistical Society. Series B (Methodological)*, *57*(1), 289–300. 10.2307/2346101

[CR10] Bettman, J. R., Luce, M. F., & Payne, J. W. (1998). Constructive consumer choice processes. *Journal of Consumer Research,**25*(3), 187–217. 10.1086/209535

[CR11] Bickel, W. K., Athamneh, L. N., Basso, J. C., Mellis, A. M., DeHart, W. B., Craft, W. H., & Pope, D. (2019). Excessive discounting of delayed reinforcers as a trans-disease process: Update on the state of the science. *Current Opinion in Psychology,**30*, 59–64. 10.1016/j.copsyc.2019.01.005 30852411 10.1016/j.copsyc.2019.01.005 PMC6684865

[CR12] Bickel, W. K., Jarmolowicz, D. P., Mueller, E. T., Koffarnus, M. N., & Gatchalian, K. M. (2012). Excessive discounting of delayed reinforcers as a trans-disease process contributing to addiction and other disease-related vulnerabilities: Emerging evidence. *Pharmacology and Therapeutics,**134*(3), 287–297. 10.1016/j.pharmthera.2012.02.004 22387232 10.1016/j.pharmthera.2012.02.004 PMC3329584

[CR13] Burghoorn, F., Roelofs, K., Burk, W. J., Jorgensen, T. D., Scheres, A., & Figner, B. (2025). *Intertemporal impatience across mental health in a community sample: A novel transdiagnostic approach*. PsyArXiv Preprints. 10.31234/osf.io/cuvn7

[CR14] Burghoorn, F., Scheres, A., Monterosso, J., Guo, M., Luo, S., Roelofs, K., & Figner, B. (2024). Pavlovian impatience: The anticipation of immediate rewards increases approach behaviour. *Cognitive, Affective, & Behavioral Neuroscience*10.3758/s13415-024-01236-2 10.3758/s13415-024-01236-2 PMC11906527 39467981

[CR15] Bürkner, P.-C. (2018). Advanced Bayesian multilevel modeling with the R package brms (Version 2.21.0) [Computer software]. *The R Journal*, *10*(1), 395–411. 10.32614/RJ-2018-017

[CR16] Cavanagh, J. F., Eisenberg, I., Guitart-Masip, M., Huys, Q., & Frank, M. J. (2013). Frontal theta overrides Pavlovian learning biases. *Journal of Neuroscience,**33*(19), 8541–8548. 10.1523/JNEUROSCI.5754-12.2013 23658191 10.1523/JNEUROSCI.5754-12.2013 PMC3707146

[CR17] Cuthbert, B. N., & Insel, T. R. (2013). Toward the future of psychiatric diagnosis: The seven pillars of RDoC. *BMC Medicine*, *11*(1). 10.1186/1741-7015-11-126 10.1186/1741-7015-11-126 PMC3653747 23672542

[CR18] Dayan, P., Niv, Y., Seymour, B., & D. Daw, N. (2006). The misbehavior of value and the discipline of the will. *Neural Networks*, *19*(8), 1153–1160. 10.1016/j.neunet.2006.03.002 10.1016/j.neunet.2006.03.002 16938432

[CR19] de Leeuw, J. R., & Gilbert, R. A. (2023). jsPsych: Enabling an open-source collaborative ecosystem of behavioral experiments. *Journal of Open Source Software*, *8*(2022), 10–13. 10.21105/joss.05351

[CR20] Decker, J. H., Figner, B., & Steinglass, J. E. (2015). On weight and waiting: Delay discounting in anorexia nervosa pretreatment and posttreatment. *Biological Psychiatry,**78*(9), 606–614. 10.1016/j.biopsych.2014.12.016 25641636 10.1016/j.biopsych.2014.12.016 PMC4478277

[CR21] Doñamayor, N., Ebrahimi, C., Garbusow, M., Wedemeyer, F., Schlagenhauf, F., & Heinz, A. (2021). Instrumental and Pavlovian mechanisms in alcohol use disorder. *Current Addiction Reports,**8*, 156–180. 10.1007/s40429-020-00333-9

[CR22] Dorfman, H. M., & Gershman, S. J. (2019). Controllability governs the balance between Pavlovian and instrumental action selection. *Nature Communications,**10*, 5826. 10.1038/s41467-019-13737-7 31862876 10.1038/s41467-019-13737-7 PMC6925275

[CR23] Fenneman, J., Frankenhuis, W. E., & Todd, P. M. (2022). In which environments is impulsive behavior adaptive? A cross-discipline review and integration of formal models. *Psychological Bulletin,**148*(7–8), 555–587. 10.1037/bul0000375

[CR24] Figner, B., Knoch, D., Johnson, E. J., Krosch, A. R., Lisanby, S. H., Fehr, E., & Weber, E. U. (2010). Lateral prefrontal cortex and self-control in intertemporal choice. *Nature Neuroscience,**13*(5), 538–539. 10.1038/nn.2516 20348919 10.1038/nn.2516

[CR25] Fontanesi, L., Gluth, S., Spektor, M. S., & Rieskamp, J. (2019). A reinforcement learning diffusion decision model for value-based decisions. *Psychonomic Bulletin and Review,**26*(4), 1099–1121. 10.3758/s13423-018-1554-2 30924057 10.3758/s13423-018-1554-2 PMC6820465

[CR26] Garbusow, M., Ebrahimi, C., Riemerschmid, C., Daldrup, L., Rothkirch, M., Chen, K., Chen, H., Belanger, M. J., Hentschel, A., Smolka, M. N., Heinz, A., Pilhatsch, M., & Rapp, M. A. (2022). Pavlovian-To-Instrumental transfer across mental disorders: A review. *Neuropsychobiology,**81*(5), 418–437. 10.1159/000525579 35843212 10.1159/000525579

[CR27] Garbusow, M., Schad, D. J., Sebold, M., Friedel, E., Bernhardt, N., Koch, S. P., Steinacher, B., Kathmann, N., Geurts, D. E. M., Sommer, C., Müller, D. K., Nebe, S., Paul, S., Wittchen, H. U., Zimmermann, U. S., Walter, H., Smolka, M. N., Sterzer, P., Rapp, M. A., … Heinz, A. (2016). Pavlovian-to-instrumental transfer effects in the nucleus accumbens relate to relapse in alcohol dependence. *Addiction Biology*, *21*(3), 719–731. 10.1111/adb.12243 10.1111/adb.12243 25828702

[CR28] Geurts, D. E. M., den Ouden, H. E. M., Janssen, L., Swart, J. C., Froböse, M. I., Cools, R., & Speckens, A. E. M. (2022a). Aversive Pavlovian inhibition in adult attention-deficit/hyperactivity disorder and its restoration by mindfulness-based cognitive therapy. *Frontiers in Behavioral Neuroscience,**16*(July), 1–11. 10.3389/fnbeh.2022.938082 10.3389/fnbeh.2022.938082 PMC9359138 35957921

[CR29] Geurts, D. E. M., Van Den Heuvel, T. J., Huys, Q. J. M., Verkes, R. J., & Cools, R. (2022b). Amygdala response predicts clinical symptom reduction in patients with borderline personality disorder: A pilot fMRI study. *Frontiers in Behavioral Neuroscience,**16*, 938403. 10.3389/fnbeh.2022.938403 36110290 10.3389/fnbeh.2022.938403 PMC9468714

[CR30] Geurts, D. E. M., von Borries, K., Huys, Q. J. M., Bulten, B. H., Verkes, R. J., & Cools, R. (2022c). Psychopathic tendency in violent offenders is associated with reduced aversive Pavlovian inhibition of behavior and associated striatal BOLD signal. *Frontiers in Behavioral Neuroscience,**16*(October), 1–14. 10.3389/fnbeh.2022.963776 10.3389/fnbeh.2022.963776 PMC9614330 36311869

[CR31] Gideon, N., Hawkes, N., Mond, J., Saunders, R., Tchanturia, K., & Serpell, L. (2016). Development and psychometric validation of the EDE-QS, a 12 item short form of the Eating Disorder Examination Questionnaire (EDE-Q). *PLOS ONE*, *11*(5). 10.1371/journal.pone.0152744 10.1371/journal.pone.0152744 PMC4854480 27138364

[CR32] Godier, L. R., & Park, R. J. (2014). Compulsivity in anorexia nervosa: A transdiagnostic concept. *Frontiers in Psychology*, *5*. 10.3389/fpsyg.2014.00778 10.3389/fpsyg.2014.00778 PMC4101893 25101036

[CR33] Guitart-Masip, M., Chowdhury, R., Sharot, T., Dayan, P., Duzel, E., & Dolan, R. J. (2012a). Action controls dopaminergic enhancement of reward representations. *Proceedings of the National Academy of Sciences of the United States of America,**109*(19), 7511–7516. 10.1073/pnas.1202229109 22529363 10.1073/pnas.1202229109 PMC3358848

[CR34] Guitart-Masip, M., Duzel, E., Dolan, R., & Dayan, P. (2014). Action versus valence in decision making. *Trends in Cognitive Sciences,**18*(4), 194–202. 10.1016/j.tics.2014.01.003 24581556 10.1016/j.tics.2014.01.003 PMC3989998

[CR35] Guitart-Masip, M., Fuentemilla, L., Bach, D. R., Huys, Q. J. M., Dayan, P., Dolan, R. J., & Duzel, E. (2011). Action dominates valence in anticipatory representations in the human striatum and dopaminergic midbrain. *Journal of Neuroscience,**31*(21), 7867–7875. 10.1523/JNEUROSCI.6376-10.2011 21613500 10.1523/JNEUROSCI.6376-10.2011 PMC3109549

[CR36] Guitart-Masip, M., Huys, Q. J. M., Fuentemilla, L., Dayan, P., Duzel, E., & Dolan, R. J. (2012b). Go and no-go learning in reward and punishment: Interactions between affect and effect. *NeuroImage,**62*, 154–166. 10.1016/j.neuroimage.2012.04.024 22548809 10.1016/j.neuroimage.2012.04.024 PMC3387384

[CR37] Heatherton, T. F., Kozlowski, L. T., Frecker, R. C., & Fagerström, K.-O. (1991). The Fagerström test for nicotine dependence: A revision of the Fagerstrom tolerance questionnaire. *British Journal of Addiction,**86*(9), 1119–1127. 10.1111/j.1360-0443.1991.tb01879.x 1932883 10.1111/j.1360-0443.1991.tb01879.x

[CR38] Herrnstein, R. J. (1961). Relative and absolute strength of response as a function of frequency of reinforcement. *Journal of the Experimental Analysis of Behavior,**4*(3), 267–272. 10.1901/jeab.1961.4-267 13713775 10.1901/jeab.1961.4-267 PMC1404074

[CR39] Horváth, Z., Nagy, L., Koós, M., Kraus, S. W., Demetrovics, Z., Potenza, M. N., Ballester-Arnal, R., Batthyány, D., Bergeron, S., Billieux, J., Briken, P., Burkauskas, J., Cárdenas-López, G., Carvalho, J., Castro-Calvo, J., Chen, L., Ciocca, G., Corazza, O., Csako, R., … Bőthe, B. (2023). Psychometric properties of the Alcohol Use Disorders Identification Test (AUDIT) across cross-cultural subgroups, genders, and sexual orientations: Findings from the International Sex Survey (ISS). *Comprehensive Psychiatry*, *127*, 152427. 10.1016/j.comppsych.2023.152427 10.1016/j.comppsych.2023.152427 37782987

[CR40] Huys, Q. J. M., Eshel, N., O’Nions, E., Sheridan, L., Dayan, P., & Roiser, J. P. (2012). Bonsai trees in your head: How the Pavlovian system sculpts goal-directed choices by pruning decision trees. *PLoS Computational Biology,**8*(3), e1002410. 10.1371/journal.pcbi.1002410 22412360 10.1371/journal.pcbi.1002410 PMC3297555

[CR41] Huys, Q. J. M., Gölzer, M., Friedel, E., Heinz, A., Cools, R., Dayan, P., & Dolan, R. J. (2016). The specificity of Pavlovian regulation is associated with recovery from depression. *Psychological Medicine,**46*(5), 1027–1035. 10.1017/S0033291715002597 26841896 10.1017/S0033291715002597 PMC4825095

[CR42] Jackson, J. N. S., & Mackillop, J. (2016). Attention-Deficit/Hyperactivity disorder and monetary delay discounting: A meta-analysis of case-control studies. *Biological Psychiatry: Cognitive Neuroscience and Neuroimaging,**1*(4), 316–325. 10.1016/j.bpsc.2016.01.007 27722208 10.1016/j.bpsc.2016.01.007 PMC5049699

[CR43] Jauregi, A., Kessler, K., & Hassel, S. (2018). Linking cognitive measures of response inhibition and reward sensitivity to trait impulsivity. *Frontiers in Psychology*, *9*. 10.3389/fpsyg.2018.02306 10.3389/fpsyg.2018.02306 PMC6279859 30546331

[CR44] Jenks, C. W., & Lawyer, S. R. (2015). Using delay discounting to understand impulsive choice in socially anxious individuals: Failure to replicate. *Journal of Behavior Therapy and Experimental Psychiatry,**46*, 198–201. 10.1016/j.jbtep.2014.10.010 25460267 10.1016/j.jbtep.2014.10.010

[CR45] Johnson, J. G., & Busemeyer, J. R. (2005). A dynamic, stochastic, computational model of preference reversal phenomena. *Psychological Review,**112*(4), 841–861. 10.1037/0033-295X.112.4.841 16262470 10.1037/0033-295X.112.4.841

[CR46] Keidel, K., Lu, X., Suzuki, S., Murawski, C., & Ettinger, U. (2024). Association of temporal discounting with transdiagnostic symptom dimensions. *Npj Mental Health Research,**3*(1), 13. 10.1038/s44184-024-00060-3 38627606 10.1038/s44184-024-00060-3 PMC11021403

[CR47] Kessler, R. C., Adler, L., Ames, M., Demler, O., Faraone, S., Hiripi, E., Howes, M. J., Jin, R., Secnik, K., Spencer, T., Ustun, T. B., & Walters, E. E. (2005). The World Health Organization adult ADHD self-report scale (ASRS): A short screening scale for use in the general population. *Psychological Medicine,**35*(2), 245–256. 10.1017/S0033291704002892 15841682 10.1017/s0033291704002892

[CR48] Kim, B. K., & Zauberman, G. (2019). Psychological time and intertemporal preference. *Current Opinion in Psychology,**26*, 90–93. 10.1016/j.copsyc.2018.06.005 30099243 10.1016/j.copsyc.2018.06.005

[CR49] Kirby, K. N., Petry, N. M., & Bickel, W. K. (1999). Heroin addicts have higher discount rates for delayed rewards than non-drug-using controls. *Journal of Experimental Psychology: General,**128*(1), 78–87. 10.1037/0096-3445.128.1.78 10100392 10.1037//0096-3445.128.1.78

[CR50] Kvam, P. D., & Busemeyer, J. R. (2020). A distributional and dynamic theory of pricing and preference. *Psychological Review,**127*(6), 1053–1078. 10.1037/rev0000215 32463254 10.1037/rev0000215 PMC8407094

[CR51] Lempert, K. M., Steinglass, J. E., Pinto, A., Kable, J. W., & Simpson, H. B. (2018). Can delay discounting deliver on the promise of RDoC? *Psychological Medicine,**49*(2), 190–199. 10.1017/S0033291718001770 30070191 10.1017/S0033291718001770

[CR52] Lenth, R. (2019). *emmeans: Estimated marginal means*. (Version 1.10.3). [Computer software]. https://cran.r-project.org/package=emmeans

[CR53] Levin, M. E., Haeger, J., Ong, C. W., & Twohig, M. P. (2018). An examination of the transdiagnostic role of delay discounting in psychological inflexibility and mental health problems. *Psychological Record,**68*(2), 201–210. 10.1007/s40732-018-0281-4

[CR54] Levitt, E. E., Oshri, A., Amlung, M., Ray, L. A., Sanchez-Roige, S., Palmer, A. A., & MacKillop, J. (2022). Evaluation of delay discounting as a transdiagnostic research domain criteria indicator in 1388 general community adults. *Psychological Medicine*10.1017/S0033291721005110 10.1017/S0033291721005110 PMC10009385 35080193

[CR55] Li, Y., Krefeld-Schwalb, A., Wall, D. G., Johnson, E. J., Toubia, O., & Bartels, D. M. (2022). The more you ask, the less you get: When additional questions hurt external validity. *Journal of Marketing Research,**59*(5), 963–982. 10.1177/00222437211073581

[CR56] Lichtenstein, S., & Slovic, P. (1971). Reversals of preference between bids and choices in gambling decisions. *Journal of Experimental Psychology,**89*(1), 46–55. 10.1037/h0031207

[CR57] Lovibond, S. H., & Lovibond, P. F. (1995). *Manual for the Depression Anxiety Stress Scales* (2nd ed.). Psychology Foundation.

[CR58] Luo, S., Ainslie, G., Giragosian, L., & Monterosso, J. R. (2009). Behavioral and neural evidence of incentive bias for immediate rewards relative to preference-matched delayed rewards. *Journal of Neuroscience,**29*(47), 14820–14827. 10.1523/JNEUROSCI.4261-09.2009 19940177 10.1523/JNEUROSCI.4261-09.2009 PMC2821568

[CR59] MacKillop, J., Amlung, M. T., Few, L. R., Ray, L. A., Sweet, L. H., & Munafò, M. R. (2011). Delayed reward discounting and addictive behavior: A meta-analysis. *Psychopharmacology (Berl),**216*(3), 305–321. 10.1007/s00213-011-2229-0 21373791 10.1007/s00213-011-2229-0 PMC3201846

[CR60] Malesza, M., & Kalinowski, K. (2021). Dark triad and impulsivity – an ecological momentary assessment approach. *Current Psychology,**40*(8), 3682–3690. 10.1007/s12144-019-00320-y

[CR61] Malesza, M., & Ostaszewski, P. (2016). Dark side of impulsivity—Associations between the Dark Triad, self-report and behavioral measures of impulsivity. *Personality and Individual Differences,**88*, 197–201. 10.1016/j.paid.2015.09.016

[CR62] Marx, I., Hacker, T., Yu, X., Cortese, S., & Sonuga-Barke, E. (2021). ADHD and the choice of small immediate over larger delayed rewards: A comparative meta-analysis of performance on simple choice-delay and temporal discounting paradigms. *Journal of Attention Disorders,**25*(2), 171–187. 10.1177/1087054718772138 29806533 10.1177/1087054718772138

[CR63] Metts, A., Arnaudova, I., Staples-Bradley, L., Sun, M., Zinbarg, R., Nusslock, R., Wassum, K. M., & Craske, M. G. (2022). Disruption in Pavlovian-instrumental ransfer as a function of depression and anxiety. *Journal of Psychopathology and Behavioral Assessment,**44*(2), 481–495. 10.1007/s10862-021-09941-9

[CR64] Millner, A. J., Gershman, S. J., Knock, M. K., & den Ouden, H. E. M. (2018). Pavlovian control of escape and avoidance. *Journal of Cognitive Neuroscience,**30*(10), 1379–1390. 10.1162/jocn 29244641 10.1162/jocn_a_01224

[CR65] Mkrtchian, A., Aylward, J., Dayan, P., Roiser, J. P., & Robinson, O. J. (2017). Modeling avoidance in mood and anxiety disorders using reinforcement learning. *Biological Psychiatry,**82*(7), 532–539. 10.1016/j.biopsych.2017.01.017 28343697 10.1016/j.biopsych.2017.01.017 PMC5598542

[CR66] Montagnese, M., Knolle, F., Haarsma, J., Griffin, J. D., Richards, A., Vertes, P. E., Kiddle, B., Fletcher, P. C., Jones, P. B., Owen, M. J., Fonagy, P., Bullmore, E. T., Dolan, R. J., Moutoussis, M., Goodyer, I. M., & Murray, G. K. (2020). Reinforcement learning as an intermediate phenotype in psychosis? Deficits sensitive to illness stage but not associated with polygenic risk of schizophrenia in the general population. *Schizophrenia Research,**222*, 389–396. 10.1016/j.schres.2020.04.022 32389614 10.1016/j.schres.2020.04.022 PMC7594641

[CR67] Morris, R. W., Quail, S., Griffiths, K. R., Green, M. J., & Balleine, B. W. (2015). Corticostriatal control of goal-directed action is impaired in schizophrenia. *Biological Psychiatry,**77*(2), 187–195. 10.1016/j.biopsych.2014.06.005 25062683 10.1016/j.biopsych.2014.06.005

[CR68] Moutoussis, M., Bullmore, E. T., Goodyer, I. M., Fonagy, P., Jones, P. B., Dolan, R. J., & Dayan, P. (2018). Change, stability, and instability in the Pavlovian guidance of behaviour from adolescence to young adulthood. *PLoS Computational Biology*, *14*(12). 10.1371/journal.pcbi.1006679 10.1371/journal.pcbi.1006679 PMC6329529 30596638

[CR69] Mullen, K. M., Ardia, D., Gil, D. L., Windover, D., & Cline, J. (2011). DEoptim: An R package for global optimization by differential evolution (Version 2.2.8) [Computer Software]. *Journal of Statistical Software*, *40*(6), 1–26. 10.18637/jss.v040.i06

[CR70] Nord, C. L., Lawson, R. P., Huys, Q. J. M., Pilling, S., & Roiser, J. P. (2018). Depression is associated with enhanced aversive Pavlovian control over instrumental behaviour. *Scientific Reports*, *8*(1). 10.1038/s41598-018-30828-5 10.1038/s41598-018-30828-5 PMC6105578 30135491

[CR71] Patros, C. H. G., Alderson, R. M., Kasper, L. J., Tarle, S. J., Lea, S. E., & Hudec, K. L. (2016). Choice-impulsivity in children and adolescents with attention-deficit/hyperactivity disorder (ADHD): A meta-analytic review. In *Clinical Psychology Review* (Vol. 43, pp. 162–174). Elsevier Inc. 10.1016/j.cpr.2015.11.001 10.1016/j.cpr.2015.11.001 26602954

[CR72] Patton, J. H., Stanford, M. S., & Barratt, E. S. (1995). Factor structure of the Barratt Impulsiveness Scale. *Journal of Clinical Psychology,**51*(6), 768–774. 10.1002/1097-4679(199511)51:6<768::AID-JCLP2270510607>3.0.CO;2-1 8778124 10.1002/1097-4679(199511)51:6<768::aid-jclp2270510607>3.0.co;2-1

[CR73] Pauli-Pott, U., & Becker, K. (2015). Time windows matter in ADHD-related developing neuropsychological basic deficits: A comprehensive review and meta-regression analysis. *Neuroscience & Biobehavioral Reviews,**55*, 165–172. 10.1016/j.neubiorev.2015.04.011 25956255 10.1016/j.neubiorev.2015.04.011

[CR74] Peterburs, J., Albrecht, C., & Bellebaum, C. (2022). The impact of social anxiety on feedback-based go and nogo learning. *Psychological Research Psychologische Forschung,**86*(1), 110–124. 10.1007/s00426-021-01479-5 33527222 10.1007/s00426-021-01479-5 PMC8821493

[CR75] R Core Team. (2024). *R: A language and environment for statistical computing* (Version 4.4.1) [Computer software]. https://www.r-project.org/

[CR76] Rounds, J. S., Beck, J. G., & Grant, D. M. M. (2007). Is the delay discounting paradigm useful in understanding social anxiety? *Behaviour Research and Therapy,**45*(4), 729–735. 10.1016/j.brat.2006.06.007 16890909 10.1016/j.brat.2006.06.007

[CR77] Saunders, J. B., Aasland, O. G., Babor, T. F., De la Fuente, J. R., & Grant, M. (1993). Development of the Alcohol Use Disorders Identification Test (AUDIT): WHO collaborative project on early detection of persons with harmful alcohol consumption-II. *Addiction,**88*(6), 791–804. 10.1111/j.1360-0443.1993.tb02093.x 8329970 10.1111/j.1360-0443.1993.tb02093.x

[CR78] Scheres, A., Balan, M., Paraskevopoulou, M., & Schellekens, A. (2024). Preference for immediate rewards in attention-deficit/hyperactivity disorder and substance use disorder: A shared intermediate phenotype? *Current Addiction Reports,**11*(4), 607–615. 10.1007/s40429-024-00558-y

[CR79] Scheres, A., de Water, E., & Mies, G. W. (2013). The neural correlates of temporal reward discounting. *Wiley Interdisciplinary Reviews: Cognitive Science,**4*(5), 523–545. 10.1002/wcs.1246 26304244 10.1002/wcs.1246

[CR80] Scheres, A., Lee, A., & Sumiya, M. (2008). Temporal reward discounting and ADHD: Task and symptom specific effects. *Journal of Neural Transmission,**115*(2), 221–226. 10.1007/s00702-007-0813-6 17876680 10.1007/s00702-007-0813-6

[CR81] Schippers, L. M., Greven, C. U., & Hoogman, M. (2024). Associations between ADHD traits and self-reported strengths in the general population. *Comprehensive Psychiatry,**130*, 152461. 10.1016/j.comppsych.2024.152461 38335571 10.1016/j.comppsych.2024.152461

[CR82] Scholz, V., Hook, R. W., Kandroodi, M. R., Algermissen, J., Ioannidis, K., Christmas, D., Valle, S., Robbins, T. W., Grant, J. E., Chamberlain, S. R., & Den Ouden, H. E. M. (2022). Cortical dopamine reduces the impact of motivational biases governing automated behaviour. *Neuropsychopharmacology,**47*, 1503–1512. 10.1038/s41386-022-01291-8 35260787 10.1038/s41386-022-01291-8 PMC9206002

[CR83] Scholz, V., Kandroodi, M. R., Algermissen, J., & Den Ouden, H. (2020). Dissociable effects of mood-anxiety and compulsive symptom dimensions on motivational biases in decision-making.pdf. *Biological Psychiatry,**87*(9), S382–S383.

[CR84] Sebold, M., Garbusow, M., Cerci, D., Chen, K., Sommer, C., Huys, Q. J. M., Nebe, S., Rapp, M., Veer, I. M., Zimmermann, U. S., Smolka, M. N., Walter, H., Heinz, A., & Friedel, E. (2021). Association of the OPRM1 A118G polymorphism and Pavlovian-to-instrumental transfer: Clinical relevance for alcohol dependence. *Journal of Psychopharmacology*. 10.1177/0269881121991992 33726538 10.1177/0269881121991992 PMC8155738

[CR85] Slovic, P. (1995). The construction of preference. *American Psychologist,**50*(5), 364–371. 10.1017/CBO9780511803475.028

[CR86] Steinglass, J. E., Figner, B., Berkowitz, S., Simpson, H. B., Weber, E. U., & Walsh, B. T. (2012). Increased capacity to delay reward in anorexia nervosa. *Journal of the International Neuropsychological Society,**18*(4), 773–780. 10.1017/S1355617712000446 22591835 10.1017/S1355617712000446 PMC3638253

[CR87] Steinglass, J. E., Lempert, K. M., Choo, T. H., Kimeldorf, M. B., Wall, M., Walsh, B. T., Fyer, A. J., Schneier, F. R., & Simpson, H. B. (2017). Temporal discounting across three psychiatric disorders: Anorexia nervosa, obsessive compulsive disorder, and social anxiety disorder. *Depression and Anxiety,**34*(5), 463–470. 10.1002/da.22586 28009473 10.1002/da.22586 PMC5869031

[CR88] Steward, T., Mestre-Bach, G., Vintró-Alcaraz, C., Agüera, Z., Jiménez-Murcia, S., Granero, R., & Fernández-Aranda, F. (2017). Delay discounting of reward and impulsivity in eating disorders: From anorexia nervosa to binge eating disorder. *European Eating Disorders Review,**25*(6), 601–606. 10.1002/erv.2543 29057603 10.1002/erv.2543

[CR89] Swart, J. C., Cook, J. L., Geurts, D. E., Frank, M. J., Cools, R., & Den Ouden, H. E. M. (2017). Catecholaminergic challenge uncovers distinct Pavlovian and instrumental mechanisms of motivated (in)action. *eLife,**6*, e22169. 10.7554/eLife.22169.001 28504638 10.7554/eLife.22169 PMC5432212

[CR90] Swart, J. C., Frank, M. J., Määttä, J. I., Jensen, O., Cools, R., & Den Ouden, H. E. M. (2018). Frontal network dynamics reflect neurocomputational mechanisms for reducing maladaptive biases in motivated action. *PLoS Biology,**16*(10), 1–25. 10.1371/journal.pbio.2005979 10.1371/journal.pbio.2005979 PMC6207318 30335745

[CR91] Takahashi, T. (2004). Cortisol levels and time-discounting of monetary gain in humans. *NeuroReport,**15*(13), 2145–2147. 10.1097/00001756-200409150-00029 15486498 10.1097/00001756-200409150-00029

[CR92] Tversky, B. A., Slovic, P., & Kahneman, D. (1990). The causes of preference reversal. *The American Economic Review,**80*(1), 204–217.

[CR93] Van Nuland, A. J., Helmich, R. C., Dirkx, M. F., Zach, H., Toni, I., Cools, R., & Den Ouden, H. E. M. (2020). Effects of dopamine on reinforcement learning in Parkinson’s disease depend on motor phenotype. *Brain*. 10.1093/brain/awaa335 33147621 10.1093/brain/awaa335 PMC7719026

[CR94] Vogel, V., Dittrich, M., Horndasch, S., Kratz, O., Moll, G. H., Erim, Y., Paslakis, G., Rauh, E., & Steins-Loeber, S. (2020). Pavlovian-to-instrumental transfer in Anorexia Nervosa: A pilot study on conditioned learning and instrumental responding to low- and high-calorie food stimuli. *European Journal of Neuroscience,**51*(8), 1794–1805. 10.1111/ejn.14592 31606905 10.1111/ejn.14592

[CR95] Vulkan, N. (2000). An economist’s perspective on probability matching. *Journal of Economic Surveys,**14*(7), 101–118. 10.1111/1467-6419.00106

[CR96] Warren, C., Mcgraw, A. P., & Van Boven, L. (2011). Values and preferences: Defining preference construction. *Wiley Interdisciplinary Reviews: Cognitive Science,**2*(2), 193–205. 10.1002/wcs.98 26302010 10.1002/wcs.98

[CR97] Wickham, H. (2016). *ggplot2: Elegant graphics for data analysis*. (Version 3.5.1) [Computer software]. Spinger-Verlag.

[CR98] Worthy, D. A., Byrne, K. A., & Fields, S. (2014). Effects of emotion on prospection during decision-making. *Frontiers in Psychology,**5*, 1–12. 10.3389/fpsyg.2014.00591 25002854 10.3389/fpsyg.2014.00591 PMC4066203

[CR99] Xia, L., Gu, R., Zhang, D., & Luo, Y. (2017). Anxious individuals are impulsive decision-makers in the delay discounting task: An ERP study. *Frontiers in Behavioral Neuroscience*, *11*. 10.3389/fnbeh.2017.00005 10.3389/fnbeh.2017.00005 PMC5258725 28174528

[CR100] Zhang, R., Chen, Z., Liu, P., & Feng, T. (2020). The neural substrates responsible for how trait anxiety affects delay discounting: Right hippocampal and cerebellar connectivity with bistable right inferior parietal lobule. *Psychophysiology,**57*(3), 1–12. 10.1111/psyp.13495 10.1111/psyp.13495 31642530

[CR101] Zhou, Y. B., Li, Q., & Liu, H. Z. (2021). Visual attention and time preference reversals. *Judgment and Decision Making,**16*(4), 1010–1038.

[CR102] Zorowitz, S., Karni, G., Paredes, N., Daw, N., & Niv, Y. (2023). *Improving the reliability of the Pavlovian go/no-go task*. 10.31234/osf.io/eb697 10.5334/cpsy.127 PMC12716264 41424918

[CR103] Zsido, A. N., Teleki, S. A., Csokasi, K., Rozsa, S., & Bandi, S. A. (2020). Development of the short version of the spielberger state—Trait anxiety inventory. *Psychiatry Research,**291*, 113223. 10.1016/j.psychres.2020.113223 32563747 10.1016/j.psychres.2020.113223

