# Supplementary material for: Intertemporal Pavlovian biases and links with mental health in the general population
Source: Cogn Affect Behav Neurosci. 2025 Aug 11;25(5):1306–29. doi: 10.3758/s13415-025-01326-9 (PMC12464115; doi:10.3758/s13415-025-01326-9)
Supplement: Supplementary file 1 — Supplementary file1 (DOCX 4.75 MB) [file 13415_2025_1326_MOESM1_ESM.docx]

**Supplementary Information**

**Intertemporal Pavlovian biases and links with mental health in the general population**

Floor Burghoorn, Anouk Scheres, Karin Roelofs, Bernd Figner

**Contents Page**

S1. Preregistered exclusion criteria 2

S2. Mental health measures 3

S3. Moderation of the intertemporal Pavlovian bias by accuracy 6

S4. Inter-individual variability in intertemporal Pavlovian biases 8

S5. Intertemporal Pavlovian bias and mental health 11

S6. Aggregated performance and mental health 31

S7. Intertemporal impatience, Pavlovian biases and mental health 35

S8. Model frequency M0 37

S9. RL model validation 42

S10. Associations RL model, mental health, and impatience 50

S11. Moderation of the intertemporal Pavlovian bias by intertemporal impatience 51

S12. Intertemporal impatience drift 52

S13. Reward valuation tasks 55

S14. Cue ratings 60

S15. High-symptom subsample analyses 62

S16. Reliability go/no-go task 81

References 85

**S1. Preregistered exclusion criteria**

Table S1 lists the preregistered exclusion criteria, and the number of participants and trials that were excluded based on each criterion. In addition to these preregistered exclusions, we excluded one participant because they did not complete the experiment, and one participant because they did not complete the experiment in English. Moreover, we excluded one participant from the analyses involving the reward ranking task, as they provided an impossible ranking score, and one participant from the analyses involving the BMI scores, as they provided an impossible BMI score. Note that the percentage of trials excluded based on the preregistered trial-level criteria are based on data from the sample of 389 participants.

**Table S1**

*Preregistered Exclusion Criteria*

| **Participant-level exclusion criteria** | ***n* excluded** |
| --- | --- |
| Failed more than 1 attention check^a^ | 21 |
| Preferred €0 today over €28 in 120 days twice in a row in the adaptive titrator | 1 |
| Did not reach stability during the adaptive titrator administered before the go/no-go task | 5 |
| Missing data on more than 5% of go/no-go trials^b^ | 4 |
| Other technical problems due to which the experiment could not be completed properly | 7 |
| More than 6 practice rounds required to complete the practice trials of the go/no-go task | 2 |
| Gave only go responses or only no-go response in the go/no-go task | 4 |
| Gave the same response on all mental health questionnaires (except for the attention checks) | 0 |
| **Trial-level exclusion criteria** | **% trials excluded** |
| Go responses with response times < 150 ms | 0.3 |
| Incomplete trials as a result of technical problems | 0.02 |

^a^These participants were rejected on Prolific (i.e., not paid) and resampled.

^b^Some participants experienced a technical problem in which some trials were skipped, or presented incorrectly (i.e., no presentation of the cue, or repeated presentation of the cue without presenting feedback). Incorrectly presented trials were removed from the data (as described in the trial-level exclusion criteria). Participants for whom more than 5% of the trials were skipped or presented incorrectly were removed from the dataset.

**S2. Mental Health Measures**

Below, we list the instruments that were used to assess mental health symptoms. The order of questionnaires was randomly determined per participant. In addition to these questionnaires, participants were asked a few questions about their menstrual cycle (if applicable). Data from these questions were collected for an unrelated project to investigate associations between the menstrual cycle and performance on various cognitive tasks, and were not of interest (and therefore not analysed) for the present study.

**Alcohol Use Disorders Identification Test (AUDIT; Saunders et al., 1993).** The AUDIT was used to assess (problematic) alcohol use. The instrument consists of 10 items. The first three items assess the frequency and quantity of general alcohol consumption, scored on a 5-point scale (0-4). If a participant indicated on the first item that they never drink alcohol, or if their scores on the second and third item were 0, all subsequent items were skipped and the scores on these items were set to 0. Items 4 to 10 assess the frequency of problematic alcohol use and adverse consequences of alcohol use. Items 4 to 8 are scored using a 5-point Likert scale, ranging from “Never” (0) to “Daily or almost daily” (4). Items 9 and 10 contain answer options “No” (0), “Yes, but not in the last year” (2), and “Yes, during the last year” (4). As described in the main text, we not only used total AUDIT sum scores (varying between 0-40), but we additionally computed separate scores for general alcohol consumption (items 1-3, scores varying between 0-12) and alcohol problems (items 4-10, scores varying between 0-28).

**Fagerström Test for Nicotine Dependence (FTND; Heatherton et al., 1991).** The FTND was used to assess nicotine dependence. The instrument consists of 7 items. The first item asks participants whether they currently smoke cigarettes, with answer options “No” (0) and “Yes” (1). We adapted this item to also include e-cigarettes (vaping). If participants indicated not to smoke, all subsequent items were skipped and the scores on these items were set to 0. Items 2-7 are items about participants’ nicotine dependence, with items 2 and 5 having 4 answer options (scored between 0-3) and the remaining items having 2 answer options (scored 0 and 1). FTND sum scores can range from 0-11. As described in the main text, we additionally used answers on the first item (reflecting whether or not a participant smokes) as a separate variable. Moreover, since the low proportion of smokers in the sample (16%) resulted in strongly zero-inflated total FTND scores, we also ran our statistical analyses with FTND scores in the subset of smokers. The first FTND item was removed from these scores (as all smokers scored 1 on this item), resulting in total scores that can range between 0-10.

**Depression Subscale of the Depression, Anxiety, and Stress Scale – 21 items (DASS-21; Lovibond & Lovibond, 1995).** We used the depression subscale of the DASS-21 to assess depression symptoms over the past week. The subscale contains 7 items, each scored on a 4-point Likert scale ranging from “Did not apply to me at all” (0) to “Applied to me very much or most of the time” (3). Sum scores can range from 0-21.

**Trait Anxiety Subscale of the Spielberger State-Trait Anxiety Inventory – Short Version (STAI-T-5; Zsido et al., 2020).** The STAI-T-5 was used to assess trait anxiety. The instrument contains 5 items, scored on a 4-point Likert scale ranging from “Almost never” (0) to “Almost always” (3). Sum scores can range between 0-15. This questionnaire contained one attention check, stating “This is an attention check. Please select ‘Sometimes’.”

**ADHD Self-Report Scale (ASRS v1.1; Kessler et al., 2005).** The ASRS was used to assess ADHD symptoms. The ASRS contains 18 items that are all scored on a 5-point Likert scale ranging from “Never” (0) to “Very Often” (4). Total sum scores can range between 0-72. As described in the main text, we additionally computed separate inattention and hyperactivity/impulsivity subscores (both consisting of 9 items, with scores varying between 0-36).

**Eating Disorder Examination Questionnaire Short (EDE-QS; Gideon et al., 2016).** We used the EDE-QS to assess eating disorder symptoms over the past 7 days. The instrument contains 12 items, the first 10 of which assess the frequency of eating disorder symptoms over the past 7 days. Answer options include “0 days” (0), “1-2 days” (1), “3-5 days” (2), “6-7 days” (3). Items 11 and 12 assess weight concerns, and are answered on a 4-point Likert scale ranging from “Not at all” (0) to “Markedly” (3). Total sum scores can range between 0-36. As described in the main text, we additionally computed a separate variable reflecting a lack of control over eating (items 9 and 10, scores varying between 0-6), and we computed a separate score that was composed of all remaining items (scores varying between 0-30).

**Barratt Impulsiveness Scale (BIS-11; Patton et al., 1995).** The BIS-11 was used to assess impulsivity. The instrument contains 30 items, 8 of which assess attentional impulsivity, 11 motor impulsivity, and 11 non-planning impulsivity. All items are scored on a 4-point Likert scale, ranging from “Rarely/Never” (0) to “Almost always/Always” (3). Items 1, 7, 8, 9, 10, 12, 13, 15, 29, and 30 were reverse coded. We computed sum scores for each of the three types of impulsivity (ranging between 0-24 for attentional impulsivity, and between 0-33 for motor and attentional impulsivity), and for the total impulsivity scores (ranging between 0-90). The questionnaire contained one attention check, stating “This is an attention check. Please select ‘Often’.”

**Body Mass Index (BMI).** Participants were asked to provide their body height (in cm) and body weight (in kg), allowing us to compute their BMI using the following equation:

BMI = $\frac{\mathrm{weight}}{{(height/100)}^{2}}$ (1)

**S3. Moderation of the Intertemporal Pavlovian Bias by Accuracy**

We examined whether individual differences in accuracy on the go/no-go task moderated the intertemporal Pavlovian bias. Individuals who are more uncertain about the appropriate instrumental response may rely more strongly on Pavlovian cues when selecting an action, possibly because these cues may increase their reward expectancy (Mahlberg et al., 2021). Therefore, they may show a stronger intertemporal Pavlovian bias effect compared to individuals with higher instrumental accuracy. It should be noted, however, that low accuracy may also be the *result* of the intertemporal Pavlovian bias interfering with instrumental actions, instead of a predictor driving this bias, and we can therefore not draw causal conclusions about any possible association between accuracy and the intertemporal Pavlovian bias effect.

To examine whether any association existed, we reran our main intertemporal Pavlovian bias model on go responding, but included the per-participant average accuracy as an additional, centered, linear fixed effect, allowing it to interact with all other fixed effects. We performed the same analysis in our previous intertemporal Pavlovian bias study (Burghoorn et al., 2024), and observed there that the effect of reward was not moderated by the average accuracy. We did observe a statistically significant three-way interaction between reward, accuracy, and required action in Burghoorn et al. (2024), but the interaction between reward and accuracy was not significant in either go or no-go trials, thus providing no support that accuracy moderated the effect of reward.

In the present study, we again observed no statistically significant two-way interaction between the reward (immediate/delayed) and accuracy (*b* = -0.78, 95% HDI [-1.71, 0.21]). We also did not observe this two-way interaction to be further moderated by the required action (*b* = -0.31, 95% HDI [-0.72, 0.06]), the task block (*b* = -0.05, 95% HDI [-0.36, 0.26]), or both (*b* = -0.14, 95% HDI [-0.40, 0.09]). Thus, again, the intertemporal Pavlovian response bias was not significantly moderated by instrumental accuracy.

We additionally ran a non-preregistered analysis to test whether accuracy moderated the intertemporal Pavlovian bias on response times (RTs). We hereby aimed to examine whether there was a general speed-accuracy trade-off in go/no-go task performance, and whether such a trade-off may specifically be present in the intertemporal Pavlovian bias effect. To this end, we reran our main intertemporal Pavlovian bias model on RTs, and again included the per-participant average accuracy as an additional, centered, linear fixed effect, allowing it to interact with all other fixed effects. There was a statistically significant main effect of accuracy on RTs (*b* = 0.19, 95% HDI [0.12, 0.26]), such that increased accuracy was associated with slower responses, reflecting a general speed-accuracy trade-off. However, accuracy did not moderate the intertemporal Pavlovian bias effect, as we did not observe a two-way interaction between accuracy and reward (*b* = -0.02, 95% HDI [-0.05, 0.002]), nor any higher-order interactions involving these variables (reward*accuracy*required action: *b* = -0.004, 95% HDI [-0.03, 0.02]; reward*accuracy*task block: *b* = -0.004, 95% HDI [-0.01, 0.02]; reward*accuracy*required action*task block: *b*= -0.004, 95% HDI [-0.02, 0.01]).

**S4. Inter-individual variability in intertemporal Pavlovian biases**

Averaged across participants, we observed the expected intertemporal Pavlovian bias effect on go responses, with increased go responding in anticipation of immediate (versus delayed) rewards. At the same time, we observed considerable inter-individual differences in this effect (see Figure S4.1), with 52% of individuals showing the effect in the expected direction (with a regression coefficient of 0.1 or higher on log-odds scale, *M*_coefficient_ = 0.89, *SD* = 1.07), while 13% of individuals showed no or a negligible effect (with a regression coefficient between -0.1 and 0.1; *M*_coefficient_ = 0.001, *SD* = 0.06), and 35% of individuals showed the opposite effect (with a regression coefficient of -0.1 or lower, *M*_coefficient_ = -0.78, *SD* = 0.89). This heterogeneity is highly similar to what we observed in Burghoorn et al. (2024). The high reliability of the response bias effect (reported in S16) suggests that this inter-individual variation is not merely driven by measurement noise, but may reflect true individual differences. We discussed possible explanations for this heterogeneity in Burghoorn et al. (2024), focusing on moderators of the intertemporal Pavlovian bias effect. Specifically, we observed weaker intertemporal Pavlovian bias effects in individuals who rated the delayed reward as higher than the immediate reward, as well as, for the no-go trials, in individuals who exhibited strong intertemporal *patience* during the choice titration. It is likely that some of these individuals showed the opposite Pavlovian bias effect, and raw data indeed pointed in this direction (for detailed results and discussion, see Burghoorn et al., 2024, Supplementary Information). In the present study, the intertemporal Pavlovian bias effect was not statistically significantly moderated by the reward valuations, nor by intertemporal impatience. Nevertheless, for the reward valuations, we observed a moderation pattern that was highly similar to that observed in Burghoorn et al. (2024), suggesting that this may have contributed—at least to some extent—to the heterogeneity in effects. This points towards the possibility that the difference in valuation of the immediate versus delayed reward contributes to the intertemporal Pavlovian bias effect. As discussed in detail in Burghoorn et al. (2024), capturing an intertemporal Pavlovian bias effect of delay that is orthogonal to an effect of valuation may prove to be conceptually and empirically complicated, as the valuation of a reward likely incorporates a delay attribute. Nevertheless, we wish to remain careful by acknowledging the role of reward valuation as a possible boundary condition of the intertemporal Pavlovian bias effect.

The substantial differences in the size and direction of the intertemporal Pavlovian response bias also raises questions about its implications for daily-life behaviours and mental health symptoms that are characterized by increased or decreased impatience. In the present study, we empirically addressed this question by studying whether individual differences in the intertemporal Pavlovian biases (in both directions) were associated with various mental health variables. For the intertemporal Pavlovian *response* bias, no such statistically significant associations were observed. A detailed discussion on the absence of these associations is provided in the main text.

We did observe associations between individual differences in the intertemporal Pavlovian *RT* bias and mental health. Figure S4.1B displays the observed inter-individual variation in the RT bias effect. Here, 52% of individuals showed faster responses in anticipation of immediate rewards (with a regression coefficient of -0.001 or lower on log scale, *M*_coefficient_ = -0.01, *SD* = 0.01), 4% of individuals showed no or a negligible effect (coefficients between -0.001 and 0.001; *M*_coefficient_ = -0.0002, *SD* = 0.001), and 44% of individuals showed the opposite effect (coefficient of 0.001 or higher; *M*_coefficient_ = 0.01, *SD* = 0.01). Since we only observed a statistically significant average RT bias on go trials, we reran our mixed-effects model on go trials separately to examine the distribution of effects in this trial type. This showed that on go trials, 56% of individuals showed faster responses in anticipation of immediate rewards (*M*_coefficient_ = -0.08, *SD* = 0.02), 4% of individuals showed no or a negligible effect (*M*_coefficient_ = -0.0001, *SD* = 0.001), and 40% of individuals showed the opposite effect (*M*_coefficient_ = 0.02, *SD* = 0.01), thus showing a pattern of inter-individual variability similar to the full task. It should be noted that the reliability analyses reported in S16 suggest that this variability may partly reflect measurement noise, in addition to true individual differences. Nevertheless, the reported associations between the individual differences in response times and mental health point towards possibly meaningful differences, suggesting that the intertemporal Pavlovian RT bias in both directions may have implications for behaviours and mental health symptoms characterized by increased impatience (e.g., impulsivity) or decreased impatience (e.g., extreme food restriction).

**Figure S4.1**

*Inter-Individual Variation in Intertemporal Pavlovian Response Bias and RT Bias*

*Note.* Distributions of the estimated effect of reward (immediate / delayed) across participants. The black vertical lines indicate the zero-point, i.e., a mean effect of 0. The blue vertical lines indicate the mean estimated effect across participants. **Panel A:** Effect of reward on go responding (i.e., the intertemporal Pavlovian response bias). Positive estimates indicate more go responding in anticipation of immediate rewards versus delayed rewards. The average response bias effect was statistically significantly different from 0. **Panel B-C:** Effect of reward on RTs (i.e., the intertemporal Pavlovian RT bias) aggregated across go and no-go trials (panel B) and separately for go trials (panel C). Negative estimates indicate faster responses in anticipation of immediate rewards versus delayed rewards. Only on go trials, the average RT bias effect was statistically significantly different from 0.

**S5. Intertemporal Pavlovian Bias and Mental Health**

To examine whether the intertemporal Pavlovian bias reported in the main text was associated with any of the mental health variables, we reran our intertemporal Pavlovian bias models on go responding (the intertemporal Pavlovian *response* bias) and on RTs (the intertemporal Pavlovian *RT* bias) while including the mental health variables as additional fixed effects, allowing them to interact with all other fixed effects. To prevent convergence issues resulting from overly large models, we ran a separate model for each mental health variable, and corrected for multiple testing by applying a Benjamini-Hochberg correction to the HDIs (with a false discovery rate of 5%; Benjamini & Hochberg, 1995). Unless specified otherwise, all effects denoted as statistically significant survived the correction for multiple testing. For the purpose of comparison of HDIs across the different tests, we report the uncorrected HDIs below (but the decision about whether an effect was statistically significant or not was based on the corrected HDIs). Our main test involved the two-way interaction between the effect of reward (immediate / delayed) and the mental health score. We additionally examined whether this two-way interaction was further moderated by the required action, task block, or both. Below, we discuss the results for each of the mental health variables, for both the response bias and the RT bias. We also present figures displaying the significant and non-significant two-way interactions between reward and the mental health scores. These figures reflect simple slopes, i.e., the effect of reward at three levels of the centered mental health scores: the mean score (*M*), 1 standard deviation below the mean (*-*1 *SD*), and 1 standard deviation above the mean (+1 *SD*). In case of statistically significant higher-order interactions involving at least the reward and a mental health variable, we also present these in figures. All figures are displaying model-based estimated marginal means and 95% HDIs. Values were back-transformed from log-odds scale to probability scale to facilitate interpretation. As a result of the involved non-linear transformations, however, the means may deviate somewhat from the raw means and 95% CIs presented in Figure 3 in the main text.

**Alcohol use disorder**

**Response bias.** We did not observe a statistically significant two-way interaction between the reward effect and the total AUDIT scores (*b* = 0.01, 95% HDI [-0.03, 0.04]), between reward and the general alcohol use (*b* = 0.001, 95% HDI [-0.06, 0.06]), nor between reward and alcohol problems (*b* = 0.02, 95% HDI [-0.03, 0.07]). The non-significant patterns are displayed in Figure S5.1. We also did not observe any statistically significant higher-order interactions involving reward and any alcohol use disorder scores.

**Figure S5.1**

*Reward by Alcohol use Disorder – Response Bias*

*Note.* Two-way interactions between the effect of reward (immediate / delayed) on go responding and the total AUDIT score (**panel A**), general alcohol use score (**panel B**), and alcohol problems score (**panel C**).

**RT bias.** In line with results for go responding, we did not observe a two-way interaction between reward and total AUDIT scores (*b* = -0.0002, 95% HDI [-0.001, 0.001], reward and general alcohol use (*b* = -0.001, 95% HDI [-0.002, 0.001]), nor reward and alcohol problems (*b* = -0.0001, 95% HDI [-0.001, 0.001]). We also did not observe any higher-order interactions involving reward and any of the alcohol use disorder variables. The results are presented in Figure S5.2.

**Figure S5.2**

*Reward by Alcohol use Disorder – RT Bias*

*Note.* Two-way interactions between the effect of reward (immediate / delayed) on response times (RTs) and the total AUDIT score (**panel A**), general alcohol use score (**panel B**) and alcohol problems score (**panel C**).

**Nicotine dependence**

**Response bias.** Although Figure S5.3 suggests increased nicotine dependence to be associated with a stronger intertemporal Pavlovian bias, and smokers (*n* = 63) to show a stronger intertemporal Pavlovian bias than non-smokers, the two-way interactions with reward were not statistically significant (reward by nicotine dependence in full sample: *b* = 0.07, 95% HDI [-0.02, 0.15]; reward by smoking status: *b* = 0.14, 95% HDI [-0.04, 0.30]; reward by nicotine dependence among smokers; *b* = 0.04, 95% HDI [-0.11. 0.21]). We also did not observe any statistically significant higher-order interactions involving reward and any of the nicotine variables.

**Figure S5.3**

*Reward by Nicotine Dependence — Response Bias*

*Note.* Two-way interactions between the effect of reward (immediate / delayed) on go responding and nicotine dependence in the full sample (**panel A**), smoking status (**panel B**), and nicotine dependence in the subsample of smokers *(n* = 63; **panel C**).

**RT bias.** We did not observe a statistically significant two-way interaction between the reward and nicotine dependence in the full sample (*b* = 0.002, 95% HDI [-0.002, 0.002]), smoking status (*b* = -0.001, 95% HDI [-0.01, 0.003]), and nicotine dependence in smokers (*b* = 0.001, 95% HDI [-0.003, 0.005]), nor did we observe any higher-order interactions involving these variables. Results are presented in Figure S5.4.

**Figure S5.4**

*Reward by Nicotine Dependence – RT Bias*

*Note.* Two-way interactions between the effect of reward (immediate / delayed) on response times (RTs) and nicotine dependence in the full sample (**panel A**), smoking status (**panel B**) and nicotine dependence in the subsample of smokers *(n* = 63; **panel C**).

**Depression**

**Response bias.** There was no statistically significant two-way interaction between reward and the depression score (*b* = -0.01, 95% HDI [-0.03, 0.02]; see Figure S5.5A). We also did not observe any statistically significant higher-order interactions involving reward and depression.

**RT bias.** We did not observe a statistically significant two-way interaction between reward and the depression score on RTs (*b* = 0.0003, 95% HDI [-0.0003, 0.001]; see Figure 2.5B), and we did not observe any statistically significant higher-order interactions involving reward and depression.

**Figure S5.5**

*Reward by Depression – Response Bias and RT Bias*

**

*Note.* Two-way interaction between the effect of reward (immediate / delayed) on go responding (**panel A**) and on response times (RTs, **panel B**) and the depression score.

**Trait anxiety**

**Response bias.** The interaction between the effects of reward and trait anxiety was not statistically significant (*b* = -0.01, 95% HDI [-0.04, 0.03]; see Figure S5.6A). There were also no statistically significant higher-order interactions involving reward and trait anxiety scores.

**RT bias.** We also did not observe a statistically significant two-way interaction between reward and trait anxiety on RTs (*b* = 0.001, 95% HDI [-0.0005, 0.002]; see Figure 2.6B), nor did we observe any statistically significant higher-order interactions involving reward and trait anxiety.

**Figure S5.6**

*Reward by Trait Anxiety – Response Bias and RT Bias*

*Note.* Two-way interaction between the effect of reward (immediate / delayed) on go responding (**panel A**) and on response times (RTs, **panel B**) and the trait anxiety score.

**ADHD**

**Response bias.** There were no statistically significant interactions between the reward and the total ADHD scores (*b* = -0.002, 95% HDI [-0.01, 0.01]), reward and inattention symptoms (*b* = -0.01, 95% HDI [-0.03, 0.01), nor between reward and hyperactivity/impulsivity symptoms (*b* = 0.01, 95% HDI [-0.02, 0.03]). We also did not observe any statistically significant higher-order interactions involving reward and any of the three ADHD scores. Results are presented in Figure S5.7.

**Figure S5.7**

*Reward by ADHD scores – Response Bias*

*Note.* Two-way interactions between the effect of reward (immediate / delayed) on go responding and the total ADHD scores (**panel A**), inattention symptoms (**panel B**) and hyperactivity/impulsivity symptoms (**panel C**).

**RT bias.** There was no statistically significant two-way interaction between reward and total ADHD scores (*b* = -0.0001, 95% HDI [-0.0004, 0.0003]), reward and inattention symptoms (*b* = -0.00002, 95% HDI [-0.001, 0.001]), nor any higher-order interactions involving these variables. The non-significant two-way interaction patterns are presented in Figure S5.8.

We also did not observe a statistically significant two-way interaction between reward and the hyperactivity/impulsivity symptoms (*b* = -0.0002, 95% HDI [-0.001, 0.0004]), but we did observe a statistically significant three-way interaction between reward, hyperactivity/impulsivity symptoms, and the required action (*b* = -0.001, 95% HDI [-0.001,

-0.0001]). Probing this three-way interaction showed there to be a statistically significant two-way interaction between reward and hyperactivity/impulsivity symptoms in go trials (i.e., on RTs of correct go responses; *b* = -0.001, 95% HDI [-0.001, -0.0001]), but not in no-go trials (i.e., on RTs of incorrect go responses; *b* = 0.0004, 95% HDI [-0.0005, 0.001]). For the go-trials, increased hyperactivity/impulsivity symptoms were associated with a stronger reward effect, with post-hoc simple slopes tests showing that individuals with average and high, but not low hyperactivity/impulsivity symptoms showed a statistically significant reward effect (low: *b* = 0.0004, 95% HDI [-0.005, 0.01]; average: *b* = -0.004, 95% HDI [-0.01, -0.00002]; high: *b* = -0.008, 95% HDI [-0.01, -0.003]; see Figure S5.9). Thus, increased hyperactivity/impulsivity symptoms were associated with faster (i.e., more vigorous) correct go responses in anticipation of immediate (versus delayed) rewards. We interpret and discuss these results in the discussion of the main text.

**Figure S5.8**

*Reward by ADHD scores – RT Bias*

*Note.* Two-way interactions between the effect of reward (immediate / delayed) on RTs, and the total ADHD (ASRS) score (**panel A**), inattention symptoms (**panel B**) and hyperactivity/impulsivity symptoms (**panel C**).

**Figure S5.9**

*Three-way Interaction Hyperactivity / Impulsivity – RT Bias*

*Note.* Statistically significant three-way interaction between the effect of reward (immediate / delayed) on response times (RTs), the required action (i.e., go or no-go trials), and the hyperactivity/impulsivity symptoms.

**Disordered eating**

**Response bias.** While Figures S5.10A and S5.10C seem to suggest somewhat stronger reward effects with more eating disorder symptoms, there were no statistically significant two-way interactions between reward and the total disordered eating score (*b* = 0.01, 95% HDI [-0.01, 0.03]), reward and a lack of control over eating (*b* = 0.01, 95% HDI [-0.09, 0.11]), nor reward and the remaining eating disorder symptoms (*b* = 0.01, 95% HDI [-0.01, 0.03]). We also did not observe any statistically significant higher-order interactions involving reward and any of the disordered eating scores.

**Figure S5.10**

*Reward by Disordered Eating – Response Bias*

*Note.* Two-way interactions between the effect of reward (immediate / delayed) on go responding, and the total disordered eating scores (**panel A**), symptoms representing a lack of control over eating (**panel B**) and remaining eating disorder symptoms (**panel C**).

**RT bias.** For the RTs, there was no statistically significant two-way interaction between reward and the total disordered eating score (*b* = 0.0004, 95% HDI [-0.0001, 0.001]; see Figure S5.11A). However, we did observe a statistically significant 3-way interaction between reward, total disordered eating, and required action (*b* = -0.001, 95% HDI [-0.001, -0.0003]), such that only for no-go trials (i.e., for incorrect go responses), there was a statistically significant interaction between the effect of reward and disordered eating scores (no-go trials: *b* = 0.001, 95% HDI [0.0004, 0.002]; go trials: -0.0003, 95% HDI [-0.001, 0.0003]). As displayed in Figure S5.12, simple slopes analyses showed that in these no-go trials, individuals with high disordered eating scores showed a *reversed* Pavlovian bias effect, with faster responses for delayed versus immediate rewards (*b* = 0.01, 95% HDI [0.003, 0.02]). Individuals with average or low disordered eating scores did not show a statistically significant intertemporal Pavlovian bias (average: *b* = 0.002, 95% HDI [-0.003, 0.01]; low: *b* = -0.01, 95% HDI [-0.01, 0.001]).

We did observe a two-way interaction between the reward and symptoms reflecting a lack of control over eating, but this interaction effect did not survive correction for multiple testing (*b* = 0.003, uncorrected 95% HDI [0.0001, 0.01], corrected 98.16% HDI [-0.003, 0.002]; see Figure S5.11B). We also observed a statistically significant three-way interaction between reward, lack of control, and required action that did survive a multiple-testing correction (*b* = -0.003, 95% HDI [-0.005, -0.001]). While there was a statistically significant interaction between lack of control over eating and the reward effect in no-go trials (*b* = 0.006, 95% HDI [0.002, 0.01]), this interaction did not reach statistical significance in go trials: *b* = -0.001, 95% HDI [-0.004, 0.002]). Post-hoc simple slopes analyses showed that in no-go trials, individuals with high lack of control over eating showed a reversed effect of reward on RTs, with faster responses for delayed compared to immediate rewards (*b* = 0.01, 95% HDI [0.003, 0.02]). Although Figure S5.13 suggests the direction of the reward effect to be the opposite for individuals with low lack of control, the reward effect was not statistically significant at average (*b* = 0.002, 95% HDI [-0.003, 0.01]) or low levels of lack of control (*b* = -0.01, 95% HDI [-0.01, 0.001]).

Finally, for the remaining eating disorder symptoms, there was no statistically significant two-way interaction with reward (*b* = 0.0004, 95% HDI [-0.0001, 0.001]; see Figure S5.11C), but we did observe a statistically significant three-way interaction between reward, remaining eating disorder symptoms, and the required action (*b* = -0.001, 95% HDI [-0.001, -0.0003]). Again, only the no-go trials showed a statistically significant two-way interaction between reward and eating disorder symptoms (no-go trials: *b* = 0.001, 95% HDI [0.0004, 0.002]; go trials: *b* = -0.0004, 95% HDI [-0.001, 0.0003]). In the no-go trials, individuals with high symptoms showed faster responses for delayed versus immediate rewards (*b* = 0.01, 95% HDI [0.002, 0.02]; see Figure S5.14). Individuals with average (*b* = 0.002, 95% HDI [-0.003, 0.01]) or low symptoms (*b* = -0.01, 95% HDI [-0.01, 0.001]) did not show a statistically significant effect of reward.

Thus, consistent across total eating disorder symptoms, symptoms reflecting a lack of control over eating, and the remaining eating disorder symptoms, high symptom levels were associated with a *reversed* intertemporal Pavlovian bias effect on RTs in no-go trials, i.e., for RTs of *incorrect* go responses. We interpret and discuss these results in the discussion of the main text.

**Figure S5.11**

*Reward by Disordered Eating – RT Bias*

*Note.* Two-way interactions between the effect of reward (immediate / delayed) on response times (RTs) and the total disordered eating (EDE) score (**panel A**), symptoms representing a lack of control over eating (**panel B**) and remaining eating disorder symptoms (**panel C**).

**Figure S5.12**

*Three-way Interaction Total Disordered Eating – RT Bias*

*Note.* Statistically significant three-way interaction between the reward, total disordered eating score, and required action.

**Figure S5.13**

*Three-way Interaction Lack of Control over Eating – RT Bias*

*Note.* Statistically significant three-way interaction between the reward, lack of control over eating, and required action.

**Figure S5.14**

*Three-way Interaction Remaining Eating Disorder Symptoms – RT Bias*

*Note.* Statistically significant three-way interaction between the reward, remaining eating disorder symptoms, and required action.

**Impulsivity**

**Response bias.** In contrast to the possible interaction patterns displayed in Figures S5.15B-C, we did not observe any statistically significant two-way interactions between reward and the total impulsivity scores (*b* = -0.0003, 95% HDI [-0.01, 0.01]), reward and attentional impulsivity (*b* = -0.03, 95% HDI [-0.06, 0.005], reward and motor impulsivity (*b* = 0.02, 95% HDI [-0.01, 0.05]), and reward and non-planning impulsivity (*b* = 0.003, 95% HDI [-0.02, 0.03]). We did observe a four-way interaction between the reward, motor impulsivity, required action, and task block, but this interaction effect did not survive a correction for multiple testing (*b* = 0.01, uncorrected 95% HDI [0.0001, 0.01], corrected 99.74% HDI [-0.004, 0.02], and we therefore refrained from probing this interaction further.

**Figure S5.15**

*Reward by Impulsivity – Response Bias*

*Note.* Two-way interactions between the effect of reward (immediate / delayed) on go responding and the total impulsivity scores (**panel A**), attentional impulsivity scores (panel B), motor impulsivity scores (**panel C**), and non-planning impulsivity scores (**panel D**).

**RT bias.** We did not observe a statistically significant two-way interaction between reward and total impulsivity scores (*b* = 0.0001, 95% HDI [-0.0003, 0.0004]; Figure S5.16A). We did, however, observe a statistically significant three-way interaction between reward, total impulsivity scores, and required action (*b* = -0.0004, 95% HDI [-0.001, -0.0002]), with a statistically significant two-way interaction between reward and total impulsivity in no-go trials (*b* = 0.001, 95% HDI [0.00004, 0.001]) but not in go trials (*b* = -0.0004, 95% HDI [-0.001, 0.00001]). In the no-go trials, individuals with high impulsivity showed a *reversed* intertemporal Pavlovian bias effect, with faster responses for delayed than immediate rewards (*b* = 0.01, 95% HDI [0.0003, 0.01]; see Figure S5.17). This pattern seemed to reverse as impulsivity scores decreased, although the Pavlovian bias effect was not statistically significant for individuals with average or low impulsivity scores (average: *b* = 0.002, 95% HDI [-0.003, 0.01]; low: *b* = -0.004, 95% HDI [-0.01, 0.003]).

Similarly, for attentional impulsivity, there was no statistically significant two-way interaction with reward (*b* = 0.001, 95% HDI [-0.001, 0.001]; see Figure S5.16B), but there was a statistically significant three-way interaction between reward, attentional impulsivity, and the required action (*b* = -0.001, 95% HDI [-0.001, -0.0001]; see Figure S5.18). However, post-hoc tests showed that although the direction of the two-way interaction between reward and attentional impulsivity was different in go and no-go trials, neither of the two-way interactions was statistically significant (go trials: *b* = -0.001, 95% HDI [-0.002, 0.0003]; no-go trials: *b* = 0.001, 95% HDI [-0.0003, 0.002]). Therefore, we did not probe these two-way interactions further.

There was no statistically significant two-way interaction between reward and motor impulsivity (*b* = 0.001, 95% HDI [-0.0003, 0.001]; Figure S5.16C), but there was a statistically significant three-way interaction between reward, motor impulsivity, and the required action (*b* = -0.001, 95% HDI [-0.002, -0.0003]; Figure S5.19). The two-way interaction between reward and motor impulsivity was statistically significant in no-go trials, but not in go trials (no-go trials: *b* = 0.001, 95% HDI [0.0003, 0.003]; go trials: *b* = -0.0004, 95% HDI [-0.001, 0.001]). In the no-go trials, individuals with high motor impulsivity showed a reversed Pavlovian bias effect, with faster responses for delayed than immediate rewards (*b* = 0.01, 95% HDI [0.001, 0.01]). Individuals with average or low motor impulsivity did not show a statistically significant Pavlovian bias effect in no-go trials (average: *b* = 0.002, 95% HDI [-0.003, 0.01]; low: *b* = -0.004, 95% HDI [-0.01, 0.003]).

Finally, again, there was no statistically significant two-way interaction between reward and non-planning impulsivity (*b* = -0.0001, 95% HDI [-0.001, 0.001]; Figure S5.16D), but there was a statistically significant three-way interaction between reward, non-planning impulsivity, and required action (*b* = -0.001, 95% HDI [-0.001, -0.0002]; Figure S5.20). Interestingly, in contrast to total impulsivity and motor impulsivity scores, the two-way interaction between reward and non-planning impulsivity was statistically significant for go trials, but not for no-go trials (go trials: *b* = -0.001, 95% HDI [-0.002, -0.0001]; no-go trials: *b* = 0.001, 95% HDI [-0.0004, 0.002]). In go trials, higher non-planning impulsivity scores were associated with a stronger intertemporal Pavlovian bias effect, as only individuals with average and high non-planning impulsivity scores showed faster responses for immediate versus delayed rewards (low: *b* = 0.0004, 95% HDI [-0.005, 0.006]; average: *b* = -0.004, 95% HDI [-0.01, -0.0002]; high: *b* = -0.01, 95% HDI [-0.01, -0.003]).

In conclusion, for the response times, all impulsivity scores showed a statistically significant three-way interaction with reward and the required action, but the interaction patterns are not consistent. For total impulsivity and motor impulsivity scores, we observed a significant two-way interaction between reward and impulsivity in no-go trials only. Here, individuals with high impulsivity scores showed a *reversed* intertemporal Pavlovian bias effect, with faster responding for delayed than immediate rewards. For non-planning impulsivity, in contrast, we observed a significant two-way interaction between reward and impulsivity in go trials only. Here, individuals with high impulsivity scores showed faster responding for immediate than delayed rewards. For attentional impulsivity, neither go nor no-go trials showed a statistically significant two-way interaction between the reward and impulsivity scores. We return to a discussion and interpretation of these results in the discussion of the main text.

**Figure S5.16**

*Reward by Impulsivity – RT Bias*

*Note.* Two-way interactions between the effect of reward (immediate / delayed) on response times (RTs), and the total impulsivity (BIS-11) score (**panel A**), attentional impulsivity scores (**panel B**), motor impulsivity scores (**panel C**), and non-planning impulsivity scores (**panel D**).

**Figure S5.17**

*Three-way Interaction Total Impulsivity – RT Bias*

*Note.* Statistically significant three-way interaction between the reward, total impulsivity, and required action.

**Figure S5.18**

*Three-way Interaction Attentional Impulsivity – RT Bias*

**

*Note.* Statistically significant three-way interaction between the reward, attentional impulsivity, and required action.

**Figure S5.19**

*Three-way Interaction Motor Impulsivity – RT Bias*

**

*Note.* Statistically significant three-way interaction between the reward, motor impulsivity, and required action.

**Figure S5.20**

*Three-way Interaction Non-Planning Impulsivity – RT Bias*

**

*Note.* Statistically significant three-way interaction between the reward, non-planning impulsivity, and required action.

**Body Mass Index**

**Response bias.** The two-way interaction between reward and BMI was not statistically significant (*b* = 0.001, 95% HDI [-0.03, 0.03]; see Figure S5.21A). We also did not observe any statistically significant higher-order interactions involving reward and BMI.

**RT bias.** In line with the response bias results, we did not observe a statistically significant two-way interaction between reward and BMI (*b* = 0.0005, 95 HDI [-0.0003, 0.001]; see Figure S5.21B), nor any higher-order interactions involving these variables.

**Figure S5.21**

*Reward by BMI – Response Bias and RT Bias*

*Note.* Two-way interaction between the effect of reward (immediate / delayed) on go responding (**panel A**) and response times (RTs, **panel B**) and Body Mass Index (BMI).

**S6. Aggregated Performance and Mental Health**

As reported in the main text and above, we tested for the association between the intertemporal Pavlovian bias and mental health by running our main trial-level mixed-effects model while additionally including the mental health variables as fixed effects, allowing them to interact with all other fixed effects. We ran a separate model for each mental health variable, and corrected for multiple testing using the Benjamini-Hochberg correction. We examined whether the effect of reward was moderated by the mental health variables (reflected by statistically significant two-way interactions between the reward and mental health variables), and whether these two-way interactions were further moderated by the required action, task block, or both. We believe that this statistical approach forms an appropriate and sufficiently conservative test of the associations between the intertemporal Pavlovian bias and mental health.

Several previous studies on valence-based Pavlovian biases, some of which also examined the association with mental health, adopted a complementary or alternative approach, which involves examining aggregated performance on the different go/no-go trials, for instance by comparing performance on trials that are congruent with the Pavlovian bias with those that are incongruent with the Pavlovian bias (e.g., go to win reward versus go to avoid punishment trials, and no-go to avoid punishment versus no-go to win reward trials; (Albrecht et al., 2016; Cavanagh et al., 2013; Mkrtchian et al., 2017; Montagnese et al., 2020; Moutoussis et al., 2018). To facilitate comparison of the present study with previous work, we ran additional, non-preregistered analyses using this approach. More specifically, we ran binomial generalized linear models with aggregated performance (i.e., proportion correct) on each of the go/no-go trial types as the dependent variable, and the mental health variables and intertemporal impatience as predictors (with a separate model for each trial type and predictor). Similar to our main analyses, we applied a Benjamini-Hochberg correction for multiple testing, and only report the statistically significant results that survived this correction below. We acknowledge that running additional analyses comes with an inflated Type-I error probability, and analysing aggregated performance measures is suboptimal compared to running a mixed-effects model on trial-level binary response variables. At the same time, we wish to avoid overlooking possible relevant patterns in the data, hereby aiming to reduce the Type-II error probability. Therefore, we report the results of these additional analyses below, but point out that these should be interpreted with extra caution.

Performance on **go to win immediate reward trials** was *negatively* associated with nicotine dependence in smokers (*b* = -0.10, *SE* = 0.02, *p* < .001), lack of control over eating (*b* = -0.06, *SE* = 0.01, *p* < .001), total impulsivity (*b* = -0.01, *SE* = 0.002, *p* < .001), motor impulsivity (*b* = -0.03, *SE* = 0.004, *p* < .001), non-planning impulsivity (*b* = -0.01, *SE* = 0.003, *p* < .001), and BMI (*b* = -0.04, *SE* = 0.003, *p* < .001), but *positively* associated with total ADHD symptoms (*b* = 0.01, *SE* = 0.002, *p* < .001), ADHD inattention symptoms (*b* = 0.01, *SE* = 0.003, *p* < .001), ADHD hyperactivity/impulsivity symptoms (*b* = 0.01, *SE* = 0.003, *p* < .001).

Performance on **go to win delayed reward trials** was *negatively* associated with intertemporal impatience (*b* = -0.01, *SE* = 0.002, *p* < .001), alcohol problems (*b* = -0.02, *SE* = 0.01, *p* < .001), being a smoker (*b* = -0.12, *SE* = 0.02, p < .001), nicotine dependence in the full sample (*b* = -0.07, *SE* = 0.01, *p* < .001), nicotine dependence in smokers (*b* = -0.07, *SE* = 0.02, *p* < .001), total eating disorder symptoms (*b* = -0.01, *SE* =0.002, *p* < .001), lack of control over eating (*b* = -0.06, *SE* = 0.01, *p* < .001), remaining eating disorder symptoms (*b* = -0.01, *SE* = 0.003, *p* < .001), total impulsivity (*b* = -0.01, *SE* = 0.002, *p* < .001), motor impulsivity (*b* = -0.05, *SE* = 0.004, *p* < .001), non-planning impulsivity (*b* = -0.03, *SE* = 0.003, *p* < .001), and BMI (*b* = -0.02, *SE* = 0.003, *p* < .001), but *positively* associated with depression (*b* = 0.02, *SE* = 0.003, *p* < .001), anxiety (*b* = 0.02, *SE* = 0.005, *p* < .001), total ADHD symptoms (*b* = 0.01, *SE* = 0.002, *p* < .001), ADHD inattention symptoms (*b* = 0.02, *SE* = 0.003, *p* < .001), and attentional impulsivity (*b* = 0.04, *SE* = 0.004, *p* < .001).

Performance on **no-go to win immediate reward trials** was *negatively* associated with intertemporal impatience (*b* = -0.01, *SE* = 0.001, *p* < .001), total alcohol use disorder symptoms (*b* = -0.01, *SE* = 0.003, *p* = .012), alcohol problems (*b* = -0.03, *SE* = 0.01, *p* < .001), being a smoker (*b* = -0.13, *SE* = 0.02, *p* < .001), nicotine dependence in the full sample (*b* = -0.11, *SE* = 0.01, *p* < .001), nicotine dependence in smokers (*b* = -0.18, *SE* = 0.02, *p* < .001), depression (*b* = -0.01, *SE* = 0.003, *p* = .002), ADHD hyperactivity/impulsivity symptoms (*b* = -0.01, *SE* = 0.003, *p* < .001), total eating disorder symptoms (*b* = -0.03, *SE* = 0.002, *p* < .001), lack of control over eating (*b* = -0.10, *SE* = 0.01, *p* < .001), remaining eating disorder symptoms (*b* = -0.03, *SE* = 0.003, *p* < .001), total impulsivity (*b* = -0.004, *SE* = 0.001, *p* = .006), motor impulsivity (*b* = -0.04, *SE* = 0.004, *p* < .001), and BMI (*b* = -0.02, *SE* = 0.003, *p* < .001). Performance on these trials was *positively* associated with attentional impulsivity (*b* = 0.02, *SE* = 0.004, *p* < .001).

Finally, performance in **no-go to win delayed reward trials** was *negatively* associated with total alcohol use disorder symptoms (*b* = -0.01, *SE* = 0.003, *p* = < .001), general alcohol use (*b* = -0.02, *SE* = 0.01, *p* = .021), alcohol problems (*b* = -0.03, *SE* = 0.01, *p* < .001), being a smoker (*b* = -0.05, *SE* = 0.02, *p* = .016), nicotine dependence in the full sample (*b* = -0.05, *SE* = 0.01, *p* < .001), nicotine dependence in smokers (*b* = -0.10, *SE* = 0.02, *p* < .001), depression (*b* = -0.01, *SE* = 0.003, *p* = .003), total ADHD symptoms (*b* = -0.005, *SE* = 0.002, *p* = .002), ADHD hyperactivity/impulsivity symptoms (*b* = -0.01, *SE* = 0.003, *p* < .001), total eating disorder symptoms (*b* = -0.02, *SE* = 0.002, *p* < .001), lack of control over eating (*b* = -0.08, *SE* = 0.01, *p* < .001), remaining eating disorder symptoms (*b* = -0.02, *SE* = 0.003, *p* < .001), total impulsivity (*b* = -0.005, *SE* = 0.001, *p* < .001), motor impulsivity (*b* = -0.02, *SE* = 0.004, *p* < .001), and BMI (*b* = -0.01, *SE* = 0.003, *p* < .001). Performance on these trials was not *positively* associated with any of the mental health variables.

Thus, in sum, in contrast to the results of our mixed-effects models on the intertemporal Pavlovian bias and mental health, we did find several statistically significant associations between aggregated performance on the different go/no-go trials and mental health symptoms. While these results may reflect associations between mental health symptoms and an increased or decreased intertemporal Pavlovian bias, they may also reflect associations between mental health and *general* performance deficits or advantages. Therefore, we next examined whether the associations between aggregated performance and mental health were stronger for the Pavlovian **bias-incongruent trials** (go to win delayed reward and no-go to win immediate reward) than for the **bias-congruent trials** (go to win immediate reward and no-go to win delayed reward trials), or vice versa, which would provide stronger evidence for an association between an altered intertemporal Pavlovian bias and mental health. We computed the difference in performance (i.e., proportion correct) between go to win immediate reward (congruent) minus go to win delayed reward (incongruent) trials, as well as the difference in performance between no-go to win delayed reward (congruent) minus no-go to win immediate reward (incongruent). Next, we computed the Pearson correlations between these performance difference scores and the mental health and intertemporal impatience variables. Although the direction of all correlations was positive, suggesting an association between mental health and a congruency effect, none of the correlations reached statistical significance. Thus, we conclude that the associations between mental health, intertemporal impatience and performance on the four different go/no-go trials reported above likely reflected general performance deficits or advantages, instead of an altered intertemporal Pavlovian bias. One alternative possibility is that some of these associations reflected an association with the go bias, i.e., the general tendency to make go responses, and an altered ability to overcome this bias. An increased go bias in individuals with elevated ADHD symptoms may, for instance, explain the positive association with performance on go trials but the negative association with performance on no-go trials. It should also be noted, however, that we did not observe any statistically significant associations between the computational go bias parameter and mental health symptoms (see S10 for details).

In sum, when considering the congruency analyses, the results of our trial-level mixed-effects models were consistent with the results of the aggregated performance models, providing no evidence of associations between the intertemporal Pavlovian bias and mental health. As also discussed in the main text, the results reported here also imply that previously reported associations between Pavlovian biases and mental health that were *solely* based on aggregated performance on the different go/no-go trials (*without* congruency analyses) may have been confounded by general performance advantages or deficits. We recommend that such association are interpreted with caution, and that future studies conduct mixed-effects models on trial-level response data, or run congruency analyses on aggregated performance.

**S7. Intertemporal Impatience, Pavlovian Biases and Mental Health**

**Figure S7.1**

*Correlations Intertemporal Impatience, Pavlovian Biases and Mental Health*

**

Note. Heatmap of the bivariate Pearson correlations between the mental health variables, intertemporal impatience, the intertemporal Pavlovian response bias, the intertemporal Pavlovian RT bias, the difference in reward ratings between the immediate versus delayed reward (with higher scores indicating that the immediate reward is valued more), and the difference in reward rankings between the delayed versus immediate reward (with higher scores indicating that the immediate^[[1]](#footnote-1)^ reward is valued more). Correlations were computed using pairwise deletion, accounting for the variables with a lower sample size (i.e., BMI, n = 388; rating difference, n = 194; ranking difference, n = 194; nicotine dependence in smokers, n = 63). Statistically significant correlations are displayed in bold. Please note, however, that the correlations involving the intertemporal Pavlovian bias effects and the reward valuation scores have been included to provide a quick descriptive overview of associations, but do not form the statistical tests of our hypotheses.

**S8. Model Frequency M0**

In response to a question by a reviewer, we investigated the relatively strong model frequency evidence in favour of M0. At first sight, it may seem counterintuitive that this criterion showed relatively strong support for such a simple model (though Scholz et al., 2022 also found relatively similar model frequencies between a basic Rescorla-Wagner model, a go-bias model, and a Pavlovian bias model). A possible technical reason for this lies in the nature of the model frequency criterion. This criterion simply tracks which AIC value was lowest for each individual, while neglecting the quantitative differences in AIC values between models. Thus, while there may be a surprisingly large number of individuals for whom M0 fitted best, the margin with which M0 was the winning model may have been small. In other words, for these individuals, there might actually not be much difference in model fit between the models—yet this information is ignored by the model frequency criterion. Figure S8.1 below supports this idea, showing that for individuals for whom M0 fitted best, the median AIC values between the five models are all quite similar. A similar pattern is observed for individuals for whom M1 fitted best (panel B). In contrast, panels C-F show that for individuals for whom M2-5 fitted best, a much clearer distinction is visible between M0-1 (simple models) and M2-5 (more complex models).

While forming a technical reason for the discrepancy between the different model evidence criteria (i.e., the more quantitative median AIC versus the more qualitative model frequency), the question remains why M0 won for a relatively large number of participants, even if with a very small margin. Following the reviewer’s suggestion, we examined the association between the best-fitting model and task performance (i.e., accuracy), displayed in Figure S8.2 below. This shows that participants for whom M0 formed the best-fitting model seemed to show higher task accuracy. It is of relevance here that M2-M5 all include parameters that aim to capture a bias in responding that can result in lower accuracy, either through a go bias, cue-response bias, learning bias, or a combination of these. Moreover, M1 can capture lower accuracy through its irreducible noise parameter. Therefore, it may not be surprising that the individuals for whom these “bias-models” fitted best showed lower accuracy. M0 does not include such parameters and may therefore fit best for individuals with high performance. Since 40% of participants showed relatively high accuracy (≥ 75% accuracy), this may explain why the behaviour of a relatively high number of participants was fitted best by these simpler models. Categorizing participants as high-learners (≥ 75% accuracy, *N* = 157) versus others (*N* = 232) confirms that for high-learners, M0 was the best-fitting model relatively often (but note, again, that the median AIC per model was relatively similar across models), while for others, M2-M5 fitted better (see Figure R5 below). A chi-square test of independence showed support for the non-independence between the performance category (high-learners versus others) and the best-fitting model (χ^2^(5) = 29.13, *p* < .001). It should be noted that, as detailed in S3, task accuracy did *not* statistically significantly moderate the intertemporal Pavlovian bias on behaviour, thus showing a discrepancy between behavioural and model results. Nevertheless, these results provide a possible reason for the somewhat surprising model frequency results for M0. To the best of our knowledge, we are not aware of other studies in the field on Pavlovian biases that have examined the possible association between task accuracy and model frequency, but this might be of interest for future work.

**Figure S8.1**

*Model Fits Per Subgroup*

*Note.* Model fits of the six RL models, separately for the individuals for whom M0 (**panel A**), M1 (**panel B**), M2 (**panel C**), M3 (**panel D**), M4 (**panel E**), and M5 (**panel F**) fitted best. The ranges of the y-axes were kept constant across panels to aid comparison across panels. Median AIC values were used as a measure of model fit.

**Figure S8.2**

*Association Task Accuracy and Best-Fitting Model*

*Note.* Median go/no-go task accuracy (quantified as the proportion of correct responses), separately for individuals for whom M0 (*N* = 70), M1 (*N* = 23), M2 (*N* = 79), M3 (*N* = 78), M4 (*N* = 83), and M5 (*N* = 56) formed the best-fitting model.

**Figure S8.3**

*Model Fit for High-Learners versus Others*

*Note.* Model fits of the six RL models, separately for high-learners (≥ 75% accuracy on the go/no-go task) and others (< 75% accuracy). **Panels A,C**: Model frequency, indicating the proportion of individuals for whom each fitted best (i.e., had the lowest AIC value). **Panels B,D**: Median AIC value across participants for each of the six models. The ranges of the y-axes were kept constant across panels to aid comparison across panels.

**S9. RL Model Validation**

We conducted model validation for M3, M4, and M5, consisting of parameter recovery, model recovery, and posterior predictive checks.

**Parameter recovery**

For each model (M3-M5), we simulated a go/no-go dataset with 389 participants and 200 trials (analogous to the observed dataset), using the observed per-participant best-fitting parameter values as data-generating parameter values^[[2]](#footnote-2)^. Next, each model was fitted to its respective dataset, allowing us to check whether we could recover the parameter values that were used to generate the data. Parameter recovery was quantified as the Pearson correlation between the data-generating parameter values and the parameters estimated from the fitted models (i.e., the recovered parameters). A higher correlation indicates better parameter recovery. Figures S9.1-S9.3 show scatterplots of the data-generating and recovered parameters for M3-M5, visualizing their parameter recovery.

Recovery of the irreducible noise, go bias, and cue-response bias parameters was good to excellent across all three models. While M3 showed good recovery of the learning rate parameter, M4 and M5 showed less optimal recovery of their two learning rate parameters, although correlations were still statistically significant and moderately positive (ranging between .35 and .60) Finally, across all three models, recovery of the inverse temperature was consistently somewhat suboptimal, yet still in an acceptable range of .50-.60. These results are highly similar to the parameter recovery results reported in Burghoorn et al. (2024), except for a somewhat improved recovery of the inverse temperature, cue-response bias, and bias-incongruent learning rate parameters in M5 in the present study, and somewhat worse recovery of both learning rates in M4 and of the bias-congruent learning rate in M5.

**Figure S9.1**

*Parameter Recovery M3*

*Note****.*** Parameter recovery of M3 (cue-response bias model). Scatterplots represent the relation between the parameters used to generate the data (i.e., the data-generating parameters) and the parameters estimated when fitting M3 to the simulated data (i.e., the recovered parameters). Parameter recovery of the learning rate α (*r* = .71, *p* < .001), irreducible noise ξ (*r* = .90, *p* < .001), go bias *b* (*r* = .86, *p* < .001), and cue-response bias π (*r* = .87, *p* < .001) parameters was good to excellent. Parameter recovery of the inverse temperature τ (*r* = .57, *p* < .001) was somewhat less optimal, although the correlation was still moderately positive and statistically significant. The recovered ξ was significantly correlated with the recovered τ, α, and *b* parameters, and the recovered *b* was correlated with the recovered α and π parameters. However, since the data-generating version of these parameters were also correlated (except for *b* and α, with *p* = .071), the correlations between the recovered parameters were likely not introduced by the recovery process (Wilson & Collins, 2019).

**Figure S9.2**

*Parameter Recovery M4*

*Note.* Parameter recovery of M4 (learning bias model). Scatterplots represent the relation between the parameters used to generate the data (i.e., the data-generating parameters) and the parameters estimated when fitting M4 to the simulated data (i.e., the recovered parameters. Parameter recovery of the irreducible noise ξ (*r* = .87, *p* < .001) and go bias *b* (*r* = .85, *p* < .001) parameters was good to excellent. Parameter recovery of the learning rates α_0_ (*r* = .36, *p* < .001) and α_1_ (*r* = .54, *p* < .001), and of the inverse temperature τ (*r* = .54, *p* < .001) was less optimal, particularly for α_0_, but the correlations were still moderately positive and statistically significant. The recovered ξ was significantly correlated with all other recovered parameters, and the recovered α_0_ was correlated with the recovered α_1_ and *b* parameters. Most of the data-generating versions of these parameters were also correlated, except for the data-generating α_0_ and b, suggesting that the correlation between the recovered α_0_ and b (but not that between the other recovered parameters) may have been introduced by the recovery process (Wilson & Collins, 2019).

**Figure S9.3**

*Parameter Recovery M5*

*Note****.*** Parameter recovery of M5 (combined cue-response bias and learning bias model). Scatterplots represent the relation between the parameters used to generate the data (i.e., the data-generating parameters) and the parameters estimated when fitting M5 to the simulated data (i.e., the recovered parameters). Parameter recovery of the irreducible noise ξ (*r* = .93, *p* < .001), go bias *b* (*r* = .86, *p* < .001), and cue-response bias π (*r* = .79, *p* < .001) was good to excellent. Parameter recovery of the learning rates α_0_ (*r* = .37, *p* < .001) and α_1_ (*r* = .58, *p* < .001), and of the inverse temperature τ (*r* = .48, *p* < .001) was less optimal, particularly for α_0_, but the correlations were still moderately positive and statistically significant. The recovered ξ was significantly correlated with the τ, α_0,_ and α_1_ parameters, and the recovered π was correlated with the recovered τ and α_1._ Most of the data-generating versions of these parameters were also correlated, except for the data-generating π and τ, suggesting that the correlation between the recovered π and τ (but not that between the other recovered parameters) may have been introduced by the recovery process (Wilson & Collins, 2019).

**Model Recovery**

Next, we examined whether M3, M4, and M5 were distinguishable using model comparison. We used the same simulated datasets as those generated for parameter recovery, and fitted each of the three models to each of the three datasets (resulting in nine model fits). Perfect model recovery would imply that the model used to generate the data best fits the simulated data. As shown in Figure S9.4A, M3 formed the best-fitting model for data generated by M3 (evidenced by the median AIC), indicating successful model recovery. Moreover, the confusion matrix displayed in Table S9.1 shows that for a majority of participants (60%), M3 forms the best-fitting model to data generated by M3. M4 and M5, however, show less successful model recovery. Although the confusion matrix shows modest support for the recovery of M4, Figure S9.3B shows that the AIC value for M4 is highly similar to those of M3 and M5, indicating suboptimal model recovery. Moreover, Figure S9.3C shows that M5 was not well distinguishable from M3. The confusion matrix also suggests poor model recovery for M5, with only 11% of participants for whom this model forms the best-fitting model for data generated by M5.

Thus, in line with results from Burghoorn et al. (2024) where M3 showed successful model recovery, M4 and M5 did not. The observation that the three models are not perfectly distinguishable may not be too surprising given their nested nature and (partially) overlapping parameters. This may be particularly the case for M5, which forms a combination of M3 and M4. In general, it seems that the most parsimonious model, M3, shows the best model recovery. In contrast, while the complexity of M5 may have been beneficial when the model was fitted on the observed data (as evidenced by its AIC advantage, presented in the main text), it seems to go at the cost of model recovery.

**Figure S9.4**

*Model Recovery Results*

*Note.* Model recovery of M3, M4, and M5. We used the same three simulated datasets as for the parameter recovery. Each model was subsequently fitted to each of the three datasets (resulting in 9 model fits). If the models were fully distinguishable using model comparison, one would expect the model that generated the data to fit the simulated data best. Model fit is displayed using median AIC values across participants.

**Table S9.1**

*Confusion Matrix Model Recovery*

|  |  | Best-fitting model | | |
| --- | --- | --- | --- | --- |
|  |  | M3 | M4 | M5 |
| Data-generating model | M3 | .60 | .28 | .12 |
|  | M4 | .40 | .52 | .08 |
|  | M5 | .55 | .31 | .13 |

*Note.* Confusion matrix indicating, for each data-generating model, the proportion of simulated participants for whom each of the three models fitted best. For instance, for 60% of the participants simulated by M3, M3 was the best-fitting model. In the case of perfect model recovery, the matrix would be 1 on the diagonal, and 0 everywhere else. Model fit was assessed using AIC.

**Posterior Predictive Checks**

Posterior predictive checks were performed to examine whether the three models could generate data that reflected the observed behavioural pattern. To this end, we simulated 1000 datasets for each model, using the per-participant best-fitted parameters. As displayed in Figure S9.5, whereas all three models generated accurate data for the go trials, only M3 generated the intertemporal Pavlovian bias observed in the no-go trials.

**Figure S9.5**

*Posterior Predictive Checks*

*Note.* Observed behaviour on the go/no-go task (**panel A**) and the simulated behavioural patterns generated by M3 (**panel B**), M4 (**panel C**), and M5 (**panel D**).

**Conclusions**

In line with Burghoorn et al. (2024), only M3 showed a successful parameter recovery, model recovery, and posterior predictive check. Thus, again, it seems that M5’s advantage in terms of model fit, possibly driven by its complexity, went at the cost of reduced model validity.

**S10. Associations RL model, Mental Health, and Impatience**

Table S10.1 presents the Pearson correlations between the parameters of the winning RL model (M3), the mental health variables, and intertemporal impatience. The model parameters did not show any statistically significant correlations with mental health or intertemporal impatience. These results are consistent with the model-free associations between the intertemporal Pavlovian response bias and mental health reported in S5, and suggest that the RL model parameters do not provide new information in identifying such associations.

**Table S10.1**

*Correlations M3 parameters and Mental Health*

| Mental Health Variable | α | τ | ξ | *b* | π |
| --- | --- | --- | --- | --- | --- |
| Alcohol use disorder | .03 | -.01 | .05 | .04 | -.02 |
| Alcohol use | .01 | -.06 | -.04 | .02 | -.02 |
| Alcohol problems | .04 | .03 | .11* | .05 | -.02 |
| Smoking status | .01 | -.09 | .02 | .04 | .06 |
| Nicotine dependence  in full sample | .07 | -.09 | .05 | .06 | .05 |
| in smokers (*n* = 63) | .23 | -.11 | .16 | .12 | .03 |
| Depression | .03 | .04 | .06 | .03 | -.04 |
| Trait anxiety | .003 | -.03 | -.03 | .01 | .001 |
| ADHD | -.01 | .03 | .03 | .05 | .02 |
| ADHD inattention symptoms | .02 | .02 | .03 | .05 | -.04 |
| ADHD hyperactivity / impulsivity symptoms | -.03 | .03 | .02 | .04 | .08 |
| Disordered eating | .01 | .002 | .07 | .06 | .07 |
| Lack of control over eating | -.08 | -.06 | .03 | .04 | .05 |
| Remaining eating disorder symptoms | .02 | .02 | .07 | .06 | .07 |
| Impulsivity | .05 | -.02 | .07 | -.03 | .02 |
| Attentional impulsivity | -.01 | -.03 | .0004 | .02 | -.07 |
| Motor impulsivity | .04 | .02 | .13* | .003 | .07 |
| Non-planning impulsivity | .07 | -.03 | .05 | -.07 | .05 |
| Body Mass Index (*n* = 388) | -.01 | .02 | .04 | .04 | .01 |
| Intertemporal impatience | -.02 | -.08 | .02 | -.02 | .07 |

*Note.* Pearson correlations between the parameter of the winning RL model (M3) and the mental health variables. Correlations are computed using pairwise deletion. The two statistically significant correlations did not survive corrections for multiple testing. **p* < .05

**S11. Moderation of the Intertemporal Pavlovian Bias by Intertemporal Impatience**

The purpose of the first choice titration was to derive a per-participant immediate reward that was preference-matched to the delayed reward of €28 in 120 days. Using the preference-matched reward pair in the go/no-go task, we aimed to test for the effects of reward delay (immediate/delayed) on go responding beyond individual differences in intertemporal impatience. Therefore, we would expect to observe the intertemporal Pavlovian bias independent of the per-participant level of intertemporal impatience. In Burghoorn et al. (2024), however, we observed a three-way interaction between reward, impatience, and required action, such that on no-go trials, more impatience was associated with a stronger Pavlovian bias effect (i.e., more failed no-go responses in anticipation of immediate reward). This could imply that the titration did not work as intended, or that a shared underlying cognitive mechanism (e.g., impaired response inhibition) contributes to both increased impatience and a stronger intertemporal Pavlovian bias.

To examine whether, in the present study, intertemporal impatience moderated the intertemporal Pavlovian bias, we reran our main intertemporal Pavlovian bias model on go responding while adding the immediate reward amount (reflecting the level of intertemporal impatience, with lower values indicating more impatience) as linear, centered fixed effect, allowing it to interact with all other fixed effects. There was no statistically significant two-way interaction between reward and the immediate reward amount (b = -0.01, 95% HDI [-0.02, 0.01]). In contrast to Burghoorn et al. (2024), we also did not observe a three-way interaction between reward, the immediate reward amount, and required action (*b* = -0.001, 95% HDI [-0.01, 0.01]), nor any other three- or four-way interactions involving these variables (reward*immediate reward amount*task block: *b* = 0.0001, 95% HDI[-0.005, 0.005]; reward*immediate reward amount*required action*task block: *b* = 0.001, 95% HDI [-0.002, 0.004]). Thus, the intertemporal Pavlovian bias effect was not moderated by individual differences in intertemporal impatience. This is consistent with the idea that by using individualized preference-matched reward pairs in the go/no-go task, we were able to test for the intertemporal Pavlovian bias effect beyond individual differences in subjectively discounted reward value (as expressed by revealed choice preferences).

**S12. Intertemporal Impatience Drift**

As reported in the main text, on average, participants showed slightly less intertemporal impatience, and thus more patience, in the second choice titration than in the first choice titration. Although a drift in impatience could imply that the reward pairs used in the go/no-go task did not remain preference-matched throughout the experiment, the observed increase in patience should result in more go responding towards *delayed* rewards, which is the opposite of what we observed. Therefore, we consider it unlikely that this increase in patience drove the observed intertemporal Pavlovian bias effect.

Nevertheless, it remains possible that individuals who became more impatient over the course of the task showed a stronger Pavlovian bias effect (because the value of the immediate reward increased over time) compared to individuals whose impatience did not change or individuals who became more patient over the course of the task. To test for this possibility, we reran our main Pavlovian bias model while including the per-participant drift in impatience as additional, linear, centered fixed effect, allowing it to interact with all other fixed effects. The drift in impatience was computed as the difference in the immediate reward amount between the second and the first choice titration, with a larger positive [negative] difference score indicating a stronger increase in patience [impatience]. Figure S12.1 displays the distribution of the drift in the sample, showing that many participants remained stable in intertemporal impatience (i.e., showed a drift around 0). Among those who did show a drift, an increase in patience was more common than an increase in impatience.

We observed a statistically significant two-way interaction between the effect of reward (immediate / delayed) on go responding and the drift score (*b* = -0.07, 95% HDI [-0.11, -0.03]). To probe this interaction further, we ran post-hoc simple slopes analyses by testing for the effect of reward at four different levels of the drift score: the mean observed drift (i.e., a small increase in patience), 1 *SD* above the mean drift (reflecting participants who became substantially more patient), 1 *SD* below the mean drift (reflecting participants who became substantially more impatient), and at a drift of 0 (reflecting participants who became neither more patient nor impatient). These simple slopes test showed the strongest effect of reward (in the hypothesized direction) at 1 *SD* below the mean drift (i.e., for a substantial increase in impatience; *b* = 0.41, 95% HDI [0.23, 0.59]), followed by the reward effects when there was no drift (*b* = 0.26, 95% HDI [0.12, 0.38]), and at the mean drift (i.e., when becoming slightly more patient; *b* = 0.19, 95% HDI [0.06, 0.32]). There was no statistically significant effect of reward at 1 *SD* above the mean drift (i.e., for a substantial increase in patience; *b* = -0.03, 95% HDI [-0.21, 0.16]).

We also observed a statistically significant three-way interaction between reward, the drift in impatience, and the required action (*b* = -0.02, 95% HDI [-0.04, -0.01]). Post-hoc tests showed that although for both go and no-go trials, the interaction between reward and the drift was statistically significant, it was slightly stronger for go trials than for no-go trials (go trials: *b* = -0.09, 95% HDI [-0.14, -0.05]; no-go trials: *b* = -0.05, 95% HDI [-0.09, -0.006]). The simple slopes pattern was the same for go and no-go trials, with statistically significant reward effects when there was no drift (go trials: *b* = 0.29, 95% HDI [0.13, 0.45]; no-go trials: *b* = 0.22, 95% HDI [0.09, 0.35]), at the mean observed drift (go trials: *b* = 0.20, 95% HDI [0.05, 0.35]; no-go trials: *b* = 0.18, 95% HDI [0.05, 0.30]), 1 *SD* below the mean drift (go trials: *b* = 0.49, 95% HDI [0.28, 0.70]; no-go trials: *b* = 0.32, 95% HDI [0.15, 0.50]) but not at 1 *SD* above the mean drift (go trials: *b* = -0.09, 95% HDI [-0.30, 0.12]; no-go trials: *b* = 0.03, 95% HDI [-0.15, 0.22]). The results are displayed in Figure S12.2.

Together, these results suggest that individual differences in the drift in impatience moderated the intertemporal Pavlovian bias effect, such that a stronger increase in impatience was associated with a stronger Pavlovian bias effect. One possible explanation for this pattern is that for participants who became more impatient, the subjective value of the immediate reward in the go/no-go task may have increased, resulting in a stronger Pavlovian bias effect. For participants who became more patient, the opposite may have happened. We do wish to emphasize that the majority of participants either remained stable or became slightly more patient (as displayed by Figure S12.1), and these individuals did show a statistically significant Pavlovian bias in the expected direction (as displayed in Figure S12.2 and as reported above). Therefore, we consider it unlikely that the individual differences in the drift in impatience primarily drove the average intertemporal Pavlovian bias effect.

**Figure S12.1**

*Patience Drift*

*Note.* Drift in intertemporal patience, computed as the immediate reward amount derived from the second choice titration minus the immediate reward amount derived from the first choice titration. A larger positive drift indicates an increase in patience, while a larger negative drift indicates an increase in impatience. The dotted vertical, blue line represents the mean drift in our sample.

**Figure S12.2**

*Reward by Patience Drift by Required Action*

*Note.* Statistically significant three-way interaction between the effect of reward (immediate / delayed) on go responding, the drift in patience (reflected by the difference in immediate reward amount between the second and first choice titration) and the required action (go/no-go). We present the results here at four drift levels: no drift, the observed mean drift (i.e., an uncentered drift score of 0.95), 1 *SD* below the mean drift (i.e., an uncentered drift score of -2.13), and 1 *SD* above the mean drift (i.e., an uncentered drift of 4.04). The figure presents model-based estimated marginal means and 95% Highest Density Intervals (HDIs), back-transformed from the log-odds scale to probability scale to facilitate interpretation.

**S13. Reward Valuation Tasks**

In Burghoorn et al. (2024), we observed that when the rewards where evaluated outside a choice context (i.e., in a rating task), the immediate member of the preference-matched reward pair was, on average, rated as more attractive than the delayed reward. Furthermore, the difference in reward ratings moderated the intertemporal Pavlovian bias effect, such that a larger rating difference (in favour of the immediate reward) was associated with a stronger intertemporal Pavlovian bias effect. This raises the question whether the intertemporal Pavlovian bias is mostly driven by a conditioned immediacy response, or by a discrepancy in valuation of the immediate versus delayed reward. In the present study, we again examined whether the immediate and delayed were valued similarly or differently when evaluated outside a choice context. Furthermore, we again tested whether the intertemporal Pavlovian bias was moderated by the difference in reward valuation between the two rewards. As preregistered, we only examined this for the intertemporal Pavlovian response bias (not for the RT bias).

To gain more insight into the role of the type of preference elicitation method when evaluating rewards, we administered two different valuation task versions, i.e., a rating and a ranking version. This choice was informed by a study by Luo et al. (2009), who used a choice titration procedure that was nearly identical to that used in the present study, but did not observe a statistically significant difference in *rankings* between the two rewards (they did not administer a rating task). Which task version participants completed in our study was varied between-participants, and hence, each analysis reported below was run on a half of the total sample (i.e., each subsample had 194 participants). The ranking data from one participant were excluded, as they provided an impossible ranking score (i.e., a ranking of 3).

**Rating task**

A model with reward ratings (0-100) as dependent variable, reward (immediate/delayed), administration time (before / after the go/no-go task), and their interaction as fixed effects, and a random intercept for participants, showed that participants on average rated the immediate reward higher than the delayed reward (*M*_immediate_  = 73.50, 95% HDI [69.20, 77.50], *M*_delayed_ = 57.70, 95% HDI [53.70, 61.90], *b*_ImmvsGrandMean_ = 7.87, 95% HDI [5.73, 9.95]). There was no statistically significant main effect of administration time on reward ratings (*b*_PostvsGrandMean_ = 2.22, 95% HDI [-1.31, 5.70]), although a statistically significant two-way interaction between administration time and reward (*b*_PostvsGrandMean*ImmvsGrandMean_ = -2.60, 95% HDI [-4.66, -0.47]) showed that the delayed rewards (but not the immediate rewards) were rated significantly higher after the go/no-go task than before the go/no-go task (delayed rewards: *b*_PostvsGrandMean_ = 4.82, 95% HDI [0.65, 8.96]; immediate rewards: *b*_PostvsGrandMean_ = -0.38, 95% HDI [-4.67, 3.56]). The increased valuation of delayed rewards after the go/no-go task aligns with the average increased patience as observed in the choice titration procedures, and supports the consistency of preferences across the two elicitation methods (i.e., choices and ratings).

**Moderation Test.** To test whether the intertemporal Pavlovian bias was moderated by the difference in ratings, we reran our main intertemporal Pavlovian bias model on go responding while including the difference in rating between the immediate and delayed reward pair as additional linear, centered fixed effect, allowing it to interact with all other fixed effects. The difference in ratings did not interact with the effect of reward (*b* = 0.01, 95% HDI [-0.0002, 0.01]), nor did we observe any higher-order interactions involving these variables. For the purpose of comparison with Burghoorn et al. (2024), the non-significant interaction pattern is presented in Figure S13.1. This shows that the interaction pattern does suggest a stronger reward effect for larger rating differences, similar to Burghoorn et al. (2024). However, in the present study, this interaction did not reach statistical significance.

**Figure S13.1**

*Reward by Rating Difference*

*Note.* Non-significant two-way interaction between the effect of reward (immediate / delayed) on go responding and the difference in rating of the immediate versus delayed reward. A larger (positive) rating difference corresponds to a higher rating of the immediate (versus delayed) reward. We present the results here at four difference levels: no difference, the mean observed difference (i.e., an uncentered difference score of 15.45), 1 *SD* below the mean difference (i.e., an uncentered difference of -29.47), and 1 *SD* above the mean difference (i.e., an uncentered difference of 29.47). The figure presents model-based estimated marginal means and 95% Highest Density Intervals (HDIs), back-transformed from the log-odds scale to probability scale to facilitate interpretation.

**Ranking Task**

Of the participants who completed the ranking task, 20.62% ranked the immediate and delayed reward as equally attractive, 43.81% ranked the immediate reward as more attractive, and 35.57% ranked the delayed reward as more attractive. A logistic regression with a rank tie (0/1) as dependent variable, administration time (before / after the go/no-go task) as fixed effect, and a random intercept varying across participants, showed that the probability of a rank tie was lower than the probability of the two rewards being ranked differently (*b* = -3.18, 95% HDI [-6.91, -1.07]). This did not depend on the time at which the ranking task was administered (*b*_PostvsGrandMean_ = 0.63, 95% HDI [0.25, 1.89]). Further probing this effect showed that the probability of the immediate reward being ranked as most attractive was higher than the probability of a tie (*b* = -2.39, 95% HDI [-5.48, -0.34]), which, again, did not depend on administration time (*b*_PostvsGrandMean_ = 0.91, 95% HDI [0.55, 2.91]). The probability of the delayed reward being ranked as most attractive was also higher than the probability of a tie (*b* = -1.90, 95% HDI [-4.63, -0.09]), independent of administration time (*b*_PostvsGrandMean_ = 1.18, 95% HDI [0.55, 3.84]). However, the probability of the immediate reward being ranked as most attractive was not significantly higher than the probability of the delayed reward being ranked as most attractive (*b* = -0.82, 95% HDI [-3.35, 0.83]), independent of administration time (*b*_ostvsGrandMean_ = -0.20, 95% HDI [-3.02, 2.27]). Thus, whereas the two rewards were not evaluated similarly in a ranking context, neither of the two rewards was ranked as significantly more attractive than the other reward.

**Moderation Test.** The rank difference between the immediate and delayed reward could take the values -1 (delayed reward ranked higher than immediate reward), 0 (both rewards ranked equally), and 1 (immediate reward ranked higher then delayed reward). We reran our main intertemporal Pavlovian bias model on go responding while including the rank difference between the immediate and delayed reward pair as additional linear, centered fixed effect, allowing it to interact with all other fixed effects. In line with the rating results, the difference in ratings did not interact with the effect of reward (*b* = 0.01, 95% HDI [-0.01, 0.36], nor did we observe any higher-order interactions involving these variables. The non-significant interaction pattern is presented in Figure S13.2. Again, although the pattern seems to suggest that a larger rank difference is associated with a stronger Pavlovian bias effect, this interaction did not reach statistical significance.

**Summary**

In sum, despite being preference-matched based on choices, the immediate and delayed rewards were valued differently in the ranking *and* rating tasks. However, while the immediate reward was *rated* higher than the delayed reward, the immediate reward was not *ranked* higher than the delayed reward, nor was the delayed reward ranked higher than the immediate reward. The intertemporal Pavlovian bias did not significantly vary as a function of the difference in ratings or rankings between the two rewards, although Figures S13.1 and S13.2 show a pattern suggesting that the bias may have been somewhat stronger when the immediate reward was valued higher than the delayed reward. We return to a discussion of the valuation results in the Discussion of the main text.

**Figure S13.2**

*Reward by Rank Difference*

*Note.* Non-significant two-way interaction between the effect of reward (immediate / delayed) on go responding and the difference in ranking of the immediate versus delayed reward. A larger (positive) rank difference corresponds to a higher ranking of the immediate (versus delayed) reward. We present the results here at four difference levels: no difference, the mean observed difference (i.e., an uncentered difference score of 0.09), 1 *SD* below the mean difference (i.e., an uncentered difference of -0.80), and 1 *SD* above the mean difference (i.e., an uncentered difference of 0.97). The figure presents model-based estimated marginal means and 95% Highest Density Intervals (HDIs), back-transformed from the log-odds scale to probability scale to facilitate interpretation.

**S14. Cue Ratings**

We examined whether there were any systematic, pre-existing differences in valuation of the cues (i.e., gem stimuli), and whether this varied as a function of when the task was administered (i.e., before or after the go/no-go task). To this end, we ran a model with cue rating (0-100) as dependent variable, a fixed intercept, cue identity, administration time, and their interaction as fixed slopes. As random effects, we included a random intercept, cue identity and administration as random slopes, and the random correlations. Testing all possible pairwise comparisons showed that the cyan pentagon was rated as significantly more attractive than all other stimuli (i.e., more attractive than the purple diamond, *b* = 13.36, 95% HDI [10.67, 16.01]; the red triangle, *b* = 11.91, 95% HDI [9.26, 14.42]; and the gold pentagon, *b* = 4.05, 95% HDI [1.63, 6.74]). The gold pentagon, in turn, was rated as significantly more attractive than the purple diamond (*b* = 9.30, 95% HDI [5.97, 12.01]) and the red triangle (*b* = 7.86, 95% HDI [4.91, 10.82]). There was no statistically significant difference in ratings between the purple diamond and red triangle (*b* = -1.45, 95% HDI [-4.27, 1.22]). The results are displayed in Figure S14.1. By randomly assigning cues to conditions in the go/no-go task, we prevented these pre-existing valuation differences from confounding the intertemporal Pavlovian bias effect.

We also observed a small, but statistically significant effect of administration time, such that, averaged across cues, the cues were rated as slightly more attractive after versus before the go/no-go task (*b*_PostvsGrandMean_ = 1.12, 95% HDI [0.52, 1.66]).

We observed no statistically significant interactions between any stimulus comparisons and administration time, indicating that the differences in valuations between cues were not significantly different before versus after the go/no-go task.

**Figure S14.1**

*Cue Ratings*

*Note.* Raw cue ratings before (Pre) and after (Post) the go/no-go task.

**S15. High-Symptom Subsample Analyses**

As discussed in the main text, compared to clinical or at-risk samples, the severity of mental health problems in samples drawn from the general population is generally limited. Possibly, any associations between intertemporal Pavlovian biases and mental health scores may be more pronounced in samples with higher symptom scores. Therefore, in line with a reviewer’s suggestion, for each variable, we created a subsample of high-symptom individuals scoring above the 75^th^ percentile. Table S15.1 lists the cut-off scores and the number of individuals in the high-symptom group for each variable. This approach is similar to that adopted by Gillan et al. (2016), who distinguished between the top 25% and bottom 75% of symptom scores. Subsequently, for each variable, we reran our mental health moderation analyses in the high-symptom subsample to examine interaction patterns pointing towards possible associations between symptom severity and the intertemporal Pavlovian biases in this subsample. We did not rerun this analysis for nicotine dependence, however, because the high-symptom subsample consisted of the same individuals as the subgroup of smokers for whom we already reported the moderation results in S5.

Before discussing the results, it should be noted that our full sample (*N* = 389) was considerably smaller than the sample by Gillan et al. (2016; *N* = 1413), thus also resulting in smaller high-symptom subgroups, and a higher probability of Type-I (i.e., false positives) and Type-II (i.e., false negatives) errors. We were therefore mostly interested in a visual inspection of the moderation patterns, providing an initial, tentative indication of possible associations. Moreover, since these analyses were fully exploratory, we did not apply a Benjamini-Hochberg correction to Highest Density Intervals, but interpreted any statistically significant results with caution. Finally, to prevent our statistical models from becoming overly complex, we did not conduct a direct statistical test of the difference in moderation patterns between the high-symptom group and the full sample.

Below, for each variable, we displayed the two-way interaction patterns of intertemporal Pavlovian biases (i.e., the effect of reward on go responding or on RTs) and the mental health variables for the high-symptom group. To facilitate comparison, we also provided the two-way interaction patterns as observed in the *full sample* (and as also displayed in S5). Any statistically significant three-way interactions between the effect of reward, the required action (i.e., go or no-go trials) and the mental health variables in the subsample are also displayed. To somewhat restrict the number of figures, we did *not* again display the three-way interaction patterns from the full sample; these are presented in S5.

**Table S15.1**

*Cut-Offs High-Symptom Subsample*

| Variable | Possible range | 75^th^ percentile cut-off | N (%) in high group |
| --- | --- | --- | --- |
| Total alcohol use disorder  (AUDIT) | 0-40 | ≥ 6 | 82 (21%) |
| Alcohol use  (AUDIT) | 0-12 | ≥ 5 | 63 (16%) |
| Alcohol problems  (AUDIT) | 0-19 | ≥ 2 | 93 (24%) |
| Nicotine dependence  (FTND) | 0-11 | ≥ 1 | 63 (16%) |
| Depression  (DASS-21) | 0-21 | ≥ 9 | 95 (24%) |
| Trait anxiety  (STAI-T-5) | 0-15 | ≥ 9 | 87 (22%) |
| Total ADHD  (ASRS-v1.1) | 0-72 | ≥ 35 | 91 (23%) |
| ADHD inattention  (ASRS-v1.1) | 0-36 | ≥ 20 | 76 (20%) |
| ADHD hyperactivity/impulsivity  (ASRS-v1.1) | 0-36 | ≥ 17 | 95 (24%) |
| Total disordered eating  (EDE-QS) | 0-36 | ≥ 13 | 75 (19%) |
| Lack of control over eating  (EDE-QS) | 0-6 | ≥ 3 | 50 (13%) |
| Remaining eating disorder symptoms  (EDE-QS) | 0-30 | ≥ 11 | 96 (25%) |
| Total impulsivity  (BIS-11) | 0-90 | ≥ 38 | 89 (23%) |
| Attentional impulsivity  (BIS-11) | 0-24 | ≥ 12 | 87 (22%) |
| Motor impulsivity  (BIS-11) | 0-33 | ≥ 13 | 86 (22%) |
| Non-planning impulsivity  (BIS-11) | 0-33 | ≥ 16 | 93 (24%) |
| Body Mass Index |  | ≥ 25.83 | 97 (25%) |

*Note.* Scores used as 75^th^ percentile cut-off to assign participants to the high-symptom group. Since all variables, except BMI, consisted of only integer scores, the 75^th^ percentile point could lie between identical integer scores. To ensure that two participants with the same symptom score could not be placed in different groups, we only assigned participants scoring *above* the 75^th^ percentile score to the high symptom group. As a result, this group generally included less than 25% of the observations.

**Results**

For **alcohol use disorder**, Figure S15.1 points towards possible two-way interactions in the high-symptom group between the effect of reward on go responses (i.e., the intertemporal Pavlovian response bias) and AUDIT scores, with a stronger response bias as AUDIT scores increased. However, these interactions did not reach statistical significance. No interaction patterns appeared for the RT bias, in line with the results from the full sample (Figure S15.2).

Similarly, no interaction patterns appeared between the Pavlovian biases and the **depression** scores, consistent with the full sample results (Figure S15.3).

While the two-way interaction between the effect of reward (immediate / delayed) on RTs (i.e., the intertemporal Pavlovian RT bias) and **trait anxiety** scores seemed highly comparable to the full sample (Figure S15.4), this interaction was statistically significant in the high-symptom group (*b* = 0.004, 95% HDI [0.0003, 0.007]). Post-hoc tests showed, however, that the RT bias was not statistically significant at either low (-1 *SD*), average, or high (-1 *SD*) anxiety levels (note that these levels were determined relative to the *high-symptom subsample,* and, hence, all scores were relatively high compared to the *full sample*). The three-way interaction between the effect of reward on RTs, required action (go / no-go) and trait anxiety (Figure S15.5) was also statistically significant in the high-symptom subgroup, and post-hoc tests showed the effect of reward to be statistically significant in no-go trials (i.e., for incorrect go responses; *b* = 0.01, 95% HDI [0.003, 0.01]), but not in go trials (i.e., for correct go responses; *b* = -0.002, 95% HDI [-0.01, 0.003]). For no-go trials, the effect of reward on RTs was statistically significant only at high levels of trait anxiety (note, again, that this is high relative to the high-symptom subgroup), with faster responses in anticipation of *delayed* rewards (*b =* 0.02, 95% HDI [0.01, 0.04]). This moderation pattern would align with a preference towards delayed rewards in anxiety (consistent with Steinglass et al., 2017), though it remains unclear why this would only influence performance on no-go trials (i.e., for incorrect no-go responses).

For **ADHD**, a pattern appeared suggesting possible two-way interactions between the response bias and the total ADHD and inattention scores (Figure S15.6). This interaction reached statistical significance for the inattention scores (*b* = -0.10, 95% HDI [-0.17, -0.02]), and post-hoc tests showed that at low (*b* = 0.53, 95% HDI [0.23, 0.84]) and average (*b* = 0.25, 95% HDI [0.03, 0.48]), but not high (*b* = -0.03, 95% HDI [-0.32, 0.28]) inattention levels, there was a statistically significantly *reversed* response bias, with more go responses in anticipation of *delayed* rewards. The observation that no response bias was observed at very high levels of inattention may not be surprising, given that the hypothesized Pavlovian response bias requires the individual to pay attention to the Pavlovian cues that are assumed to elicit bias. The direction of the response bias at low or average levels of inattention is more surprising, however, and we cannot readily explain why inattention symptoms would be associated with increased go responding towards *delayed* rewards.

The association between the RT bias and ADHD scores appeared relatively similar to the full sample (Figure S15.7). However, in contrast to the full sample results, the three-way interaction between the RT bias and inattention scores (Figure S15.8) was statistically significant in the high-symptom group (*b* = -0.003, 95% HDI [-0.005, -0.0002]). Post-hoc tests showed a statistically significant two-way interaction in go trials (*b* = -0.004, 95% HDI [-0.01, -0.001]), but not in no-go trials (*b* = 0.002, 95% HDI [-0.003, 0.01]). In go trials, there was a statistically significant RT bias at high inattention levels only (*b* = -0.01, 95% HDI [-0.03, -0.003]), showing faster correct go responses in anticipation of immediate (versus delayed) rewards. The RT bias was not statistically significant at low (*b* = 0.005, 95% HDI [-0.01, 0.02]) or average (*b* = -0.005, 95% HDI -0.01, 0.003]) inattention levels. The specificity of the association between the RT bias and ADHD scores to inattention scores contrasts with the full sample results, which only showed an association for the hyperactivity/impulsivity scores. Moreover, the direction of the RT bias-inattention association in the high-symptom group contrasts with the above-discussed idea that high inattention might *weaken* any cue-driven Pavlovian biases. We wish to emphasize, however, that a visual inspection of the interaction patterns does not show substantial differences between the full sample and high-symptom group, and we did not conduct a direct comparison of the full sample and subsample. Therefore, we interpret any differences with caution.

The interaction patterns between the intertemporal Pavlovian biases and **eating disorder symptoms** overall appeared to be similar between the full sample and high-symptom subsample (Figure S15.9-10). In the high-symptom group, however, two-way interaction patterns seemed to appear for the symptoms reflecting a lack of control over eating, suggesting these symptoms to be associated with reversed Pavlovian biases, in line with the full sample RT bias results (but in contrast to our hypotheses). However, none of the interactions between eating disorder scores and the Pavlovian biases reached statistical significance.

Similarly, the interaction patterns between the intertemporal Pavlovian biases and **impulsivity** scores in the high-symptom group appeared to be comparable to the full sample. Similar to the full sample, we did not detect any statistically significant interactions between the response bias and impulsivity scores (Figure S15.11). Moreover, while the two-way interactions between the RT bias and impulsivity scores (Figure S15.12) were not statistically significant, we did observe some statistically significant three-way interactions, again in line with the full sample. Specifically, there was a statistically significant three-way interaction between the effect of reward on RTs, the required action, and attentional impulsivity (*b* = -0.003, 95% HDI [-0.01, -0.0001]; Figure S15.13), but, similar to the full sample results, the two-way interactions between reward and attentional impulsivity were not statistically significant in go trials (*b* = -0.004, 95% HDI [-0.01, 0.0003]) nor no-go trials (*b* = 0.003, 95% HDI [-0.002, 0.008]). We also observed a statistically significant three-way interaction between reward, required action, and non-planning impulsivity (*b* = -0.003, 95% HDI [-0.01, -0.001]; Figure S15.14). The two-way interaction between reward and non-planning impulsivity was statistically significant in go trials (*b* = -0.004, 95% HDI [-0.01, -0.001]), but not in no-go trials (*b* = 0.002, 95% HDI [-0.003, 0.01]). Consistent with the full sample results, in go trials, we observed an RT bias at high levels of non-planning impulsivity (*b* = -0.02, 95% HDI [-0.03 -0.01]), with faster correct go responses in anticipation of immediate versus delayed rewards. This effect was not statistically significant at low (*b* = 0.004, 95% HDI [-0.01, 0.02]) or average (*b* = -0.01, 95% HDI [-0.02, 0.0001]) levels of non-planning impulsivity.

Finally, the interaction pattern between the response bias and **BMI** scores (Figure S15.15) appeared to be considerably more pronounced in the high-symptom group than in the full sample, suggesting a stronger response bias (with increased go responding in anticipation of immediate rewards) as BMI scores increased. However, this interaction did not reach statistical significance in the high-symptom group, consistent with the full sample results.

**In sum**, the interaction patterns in the high-symptom group did not seem to show strong deviations from the full sample results. However, a few differences appeared. That is, (i) the association between the Pavlovian biases and AUDIT, BMI, and lack of control over eating scores seemed more pronounced in the high-symptom group, but did not reach statistical significance, and (ii) a reversed RT bias in no-go trials was statistically significantly associated with anxiety, and (iii) the Pavlovian biases were statistically significantly associated with ADHD inattention scores, but the direction of these associations was inconsistent between the response bias and RT bias.

**Figure S15.1**

*Reward by Alcohol use Disorder – Response Bias*

Full sample

Above 75^th^ percentile

*Note.* Two-way interactions between the effect of reward (immediate / delayed) on go responding and the total AUDIT score (**panel A**), general alcohol use score (**panel B**), and alcohol problems score (**panel C**), separately for the full sample (**top panel**) and for the participants scoring above the 75^th^ percentile (**bottom panel**).

**Figure S15.2**

*Reward by Alcohol use Disorder – RT Bias*

Full sample

Above 75^th^ percentile

*Note.* Two-way interactions between the effect of reward (immediate / delayed) on response times (RTs) and the total AUDIT score (**panel A**), general alcohol use score (**panel B**) and alcohol problems score (**panel C**), separately for the full sample (**top panel**) and for the participants scoring above the 75^th^ percentile (**bottom panel**).

**Figure S15.3**

*Reward by Depression – Response Bias and RT Bias*

Full sample

**

Above 75^th^ percentile

*Note.* Two-way interaction between the effect of reward (immediate / delayed) on go responding (**panel A**) and on response times (RTs, **panel B**) and the depression score, separately for the full sample (**top panel**) and for the participants scoring above the 75^th^ percentile (**bottom panel**).

**Figure S15.4**

*Reward by Trait Anxiety – Response Bias and RT Bias*

Full sample

Above 75^th^ percentile

*Note.* Two-way interaction between the effect of reward (immediate / delayed) on go responding (**panel A**) and on response times (RTs, **panel B**) and the trait anxiety score, separately for the full sample (**top panel**) and for the participants scoring above the 75^th^ percentile (**bottom panel**).

**Figure S15.5**

*Three-way interaction Trait Anxiety – RT Bias, Above 75^th^ percentile*

*Note.* Statistically significant three-way interaction between the effect of reward (immediate / delayed) on response times (RTs), the required action (i.e., go or no-go trials), and the trait anxiety scores, for the subset of individuals that scored above the 75^th^ percentile. This three-way interaction was not statistically significant in the full sample.

**Figure S15.6**

*Reward by ADHD scores – Response Bias*

Full sample

Above 75^th^ percentile

*Note.* Two-way interactions between the effect of reward (immediate / delayed) on go responding and the total ADHD scores (**panel A**), inattention symptoms (**panel B**) and hyperactivity/impulsivity symptoms (**panel C**), separately for the full sample (**top panel**) and for the participants scoring above the 75^th^ percentile (**bottom panel**).

**Figure S15.7**

*Reward by ADHD scores – RT Bias*

Full sample

Above 75^th^ percentile

*Note.* Two-way interactions between the effect of reward (immediate / delayed) on RTs, and the total ADHD (ASRS) score (**panel A**), inattention symptoms (**panel B**) and hyperactivity/impulsivity symptoms (**panel C**), separately for the full sample (**top panel**) and for the participants scoring above the 75^th^ percentile (**bottom panel**).

**Figure S15.8**

*Three-way Interaction Inattention symptoms – RT Bias, Above 75^th^ percentile.*

*Note.* Statistically significant three-way interaction between the effect of reward (immediate / delayed) on response times (RTs), the required action (i.e., go or no-go trials), and the ADHD inattention symptoms, for the subset of individuals that scored above the 75^th^ percentile. This three-way interaction was not statistically significant in the full sample.

**Figure S15.9**

*Reward by Disordered Eating – Response Bias*

Full sample

Above 75^th^ percentile

*Note.* Two-way interactions between the effect of reward (immediate / delayed) on go responding, and the total disordered eating scores (**panel A**), symptoms representing a lack of control over eating (**panel B**) and remaining eating disorder symptoms (**panel C**), separately for the full sample (**top panel**) and for the participants scoring above the 75^th^ percentile (**bottom panel**).

**Figure S15.10**

*Reward by Disordered Eating – RT Bias*

Full sample

Above 75^th^ percentile

*Note.* Two-way interactions between the effect of reward (immediate / delayed) on response times (RTs) and the total disordered eating (EDE) score (**panel A**), symptoms representing a lack of control over eating (**panel B**) and remaining eating disorder symptoms (**panel C**), separately for the full sample (**top panel**) and for the participants scoring above the 75^th^ percentile (**bottom panel**).

**Figure S15.11**

*Reward by Impulsivity – Response Bias*

Full sample

Above 75^th^ percentile

**

*Note.* Two-way interactions between the effect of reward (immediate / delayed) on go responding and the total impulsivity scores (**panel A**), attentional impulsivity scores (**panel B**), motor impulsivity scores (**panel C**), and non-planning impulsivity scores (**panel D**), separately for the full sample (**top panel**) and for the participants scoring above the 75^th^ percentile (**bottom panel**).

**Figure S15.12**

*Reward by Impulsivity – RT Bias*

Full sample

**

**

Above 75^th^ percentile

**

*Note.* Two-way interactions between the effect of reward (immediate / delayed) on response times (RTs), and the total impulsivity (BIS-11) score (**panel A**), attentional impulsivity scores (**panel B**), motor impulsivity scores (**panel C**), and non-planning impulsivity scores (**panel D**), separately for the full sample (**top panel**) and for the participants scoring above the 75^th^ percentile (**bottom panel**).

**Figure S15.13**

*Three-way Interaction Attentional Impulsivity – RT Bias, Above 75^th^ percentile*

**

*Note.* Statistically significant three-way interaction between the reward, attentional impulsivity, and required action, for the subset of individuals that scored above the 75^th^ percentile. This three-way interaction was also statistically significant in the full sample, and is displayed in Figure S5.18.

**Figure S15.14**

*Three-way Interaction Non-Planning Impulsivity – RT Bias*

**

*Note.* Statistically significant three-way interaction between the reward, non-planning impulsivity, and required action, for the subset of individuals that scored above the 75^th^ percentile. This three-way interaction was also statistically significant in the full sample, and is displayed in Figure S5.19.

**Figure S15.15**

*Reward by BMI – Response Bias and RT Bias*

Full sample

Above 75^th^ percentile

**

*Note.* Two-way interaction between the effect of reward (immediate / delayed) on go responding (**panel A**) and response times (RTs, **panel B**) and Body Mass Index (BMI), separately for the full sample (top panel) and for the participants scoring above the 75^th^ percentile (bottom panel).

**S16. Reliability go/no-go task**

The field of computational psychiatry has recently brought increased attention to the importance of the reliability of cognitive tasks and of the computational model parameters fitted to the data from these tasks (e.g., Mkrtchian et al., 2023; Zorowitz et al., 2023; Zorowitz & Niv, 2023). The reliability of a cognitive task reflects how much of individual variability is due to true individual differences relative to measurement noise. This is particularly relevant if one wishes to test for the associations between individual differences in task effects and external measures (such as self-report mental health scores) because the reliability of both measures determines the upper bound on the maximally observable correlation between the two measures. The reliability of the self-report mental health measures used in the present study (Cronbach’s α) are reported in Table 1 in the main text.

To determine the reliability of the go/no-go task, we computed the split-half reliability of the effect of reward (immediate / delayed) on go responding (i.e., the response bias) and on RTs (i.e., the RT bias). Moreover, we computed the split-half reliability of the model parameters of our winning RL model, M3.

**Response Bias.** We adopted two different approaches to computing the split-half reliability. First, as reported in the main text, the intertemporal Pavlovian response bias was quantified as the effect of reward (immediate / delayed) on trial-level responses (go/no-go), tested using a mixed-effects model. We assessed the odd-even split-half reliability of this effect by splitting the dataset in two halves, assigning trials with odd versus even trial numbers to different halves. We subsequently ran the mixed-effects model on both halves, extracted the per-participant effects (i.e., random effects) of reward, and correlated these. A Spearman-Brown correction was applied to the Pearson correlation to correct for the reduced number of trials in each of the two halves. The effects of rewards in both halves were highly correlated (*r* = .967, 95% CI [.960, 973]), indicating excellent reliability.

In the abovementioned approach, we split the data based on odd-even trial numbers. Since the decision on how to split the data is relatively arbitrary, we additionally computed the split-half reliability using a permutation-based approach. Specifically, we created 5400 random splits of the data (in line with Kahveci et al., 2024 showing that reliabilities were accurate under any examined condition with at least 5400 splits), which were stratified to ensure a (near) equal number of trials per level of reward (immediate / delayed) and required action (go / no-go). Since running our mixed-effects model on over 10,000 datasets would be practically infeasible given time and computational constraints, we quantified the intertemporal Pavlovian bias effect in a slightly different manner. That is, for each half of the data, we computed the difference in the mean proportion of go responses between the immediate and delayed reward. We then computed the Pearson correlation between the differences scores of the two halves. A Fisher *z* transformation was applied to the raw correlations before computing the average correlation to prevent the average correlation from being biased to zero. A Spearman-Brown correction was applied to the average correlation to correct for the reduced number of trials in each half compared to the full dataset. Consistent with the single-split odd-even reliability reported above, this second approach resulted in a reliability estimate of .972 (95% CI [.967, .972]). Thus, together, these results suggest that individual differences in the reward effect on go responding is likely to reflect true individual differences instead of measurement noise.

**RTs.** A near-identical approach was used to assess the reliability of the effect of reward on RTs. A difference is that for the permutation-based approach, the splits were not stratified based on required action (go / no-go), as several participants did not have sufficient observations per condition for the no-go trials (since these only included incorrect go responses, i.e., false alarms). Moreover, for the permutation-based approach, we had to remove data from 21 participants for whom we had less than two RT observations per reward level and for whom we resultingly could not split the data into two halves.

The single-split odd-even reliability on the random effect of reward on RTs was .515 (95% CI [.407, .603]), substantially lower than that for the effect of reward on go responding reported above. The permutation-based approach showed a similar, yet slightly higher reliability of .574 (95% [.463, .659]). These results suggest that the intertemporal Pavlovian *RT* bias was measured with more measurement error than the intertemporal Pavlovian *response* bias.

One possible reason for this lower reliability is that the number of observations on which the RT bias is based is lower than for the response bias, as it only includes trials on which go response were made, and measurement noise decreases as a function of the number of trials (Zorowitz & Niv, 2023). Moreover, it is tempting to speculate that compared to responses, RTs may be more sensitive to natural fluctuations in attention while completing the go/no-go task, thereby inducing measurement noise. An alternative reason for the lower reliability is a ceiling effect in response speed. By using a response window of only 600 ms, we forced participants to respond as fast as they could, possibly resulting in a ceiling effect. A range restriction has been shown to reduce the ability to differentiate between participants, decreasing the task reliability (Zorowitz & Niv, 2023).

Since the reliability of a task places an upper bound to the associations that can be detected with other variables, it is somewhat surprising that the only statistically significant associations between the intertemporal Pavlovian bias effect and the mental health variables were found for the RT bias, not for the response bias. We do wish to note that a reliability of .50-.60 reduces, but does not eliminate the possibility of finding associations. Moreover, the reported associations between the RT bias and mental health varied by the required action, and the follow-up models showed these associations to be statistically significant in either go or no-go trials only. Therefore, we additionally computed the split-half reliability of the intertemporal Pavlovian bias on RTs in go and no-go trials separately. The reliability of the effect in go trials was .739 (95% CI [.681, .786]) for the single-split odd-even approach, and .552 (95% CI [.454, .633]) for the permutation-based approach. The reliability of the effect in no-go trials was .683 (95% CI [.612, .741]) for the single-split odd-even approach, but only .206 (95% CI [.071, .324]) for the permutation-based approach. The strong divergence in results between the two reliability approaches confirms the potential influence of the type of approach selected; Kahveci et al. (2024) showed permutation-based approaches to result in more accurate reliability estimates than a single-split odd-even approach. It should also be noted, however, that to compute a difference score for the permutation-based approach, we had to exclude all participants who had less than two observations per reward level. This resulted in the exclusion of 34 participants for the go trials, and 45 participants for the no-go trials. These excluded participants showed highly consistent behaviour, with either very high or very low performance. Excluding these highly consistent participants may thus have reduced the split-half reliability. Nevertheless, both approaches show the reliability to be higher for go trials than for no-go trials.

The low reliability in no-go trials was likely—at least in part—driven by the low number of observations, as they were based on incorrect go responses only, which were substantially less common than correct responses. Moreover, although speculative, response times of incorrect responses (i.e., false alarms) may inherently be noisier than the response times of correct responses. In line with this possible explanation, the fixed effect of reward on RTs on no-go trials was not statistically significant, as reported in the main text.

**RL parameters.** Since running the RL model on over 10,000 permutations was infeasible given time and computational constraints, we exclusively computed the single-split odd-even reliability estimate for the RL parameters. We ran the RL model on both halves, and computed the Pearson correlation between the parameters of the two halves. Again, a Spearman-Brown correction was used to correct for the reduced number of trials in each half of the full dataset. This demonstrated excellent reliability of all parameters, with estimates of .974 (95% CI [.968, .978]) for the learning rate parameter α, .983 (95% CI [.979, .986]) for the inverse temperature parameter τ, .984 (95% CI [.980, .987]) for the irreducible noise parameter ξ, .985 (95% CI [.983, .988]) for the go bias parameter *b*, and .993 (95% CI [.992, .995]) for the Pavlovian cue-response bias parameter π. This indicates that individual differences in the RL parameters are likely to reflect true individual differences in these RL processes, instead of measurement noise.

**Conclusions.** In sum, we conclude that the reliability of intertemporal Pavlovian *response* bias was excellent, as demonstrated through both singe-split and permutation-based reliability analyses. Moreover, the parameters of the RL model fitted to these response data showed excellent reliability. The reliability of the intertemporal Pavlovian *RT* bias was lower, in particular for the no-go trials. Therefore, we interpret the results on the associations between these RT effects and the mental health associations with caution, and recommend future research to attempt to replicate these associations.

**References**

Albrecht, M. A., Waltz, J. A., Cavanagh, J. F., Frank, M. J., & Gold, J. M. (2016). Reduction of Pavlovian bias in schizophrenia: Enhanced effects in clozapine-administered patients. *PLoS ONE*, *11*(4), 1–23. https://doi.org/10.1371/journal.pone.0152781

Benjamini, Y., & Hochberg, Y. (1995). Controlling the False Discovery Rate: A practical and powerful approach to multiple testing. *Journal of the Royal Statistical Society. Series B (Methodological)*, *57*(1), 289–300. https://doi.org/10.2307/2346101

Burghoorn, F., Scheres, A., Monterosso, J., Guo, M., Luo, S., Roelofs, K., & Figner, B. (2024). Pavlovian impatience: The anticipation of immediate rewards increases approach behaviour. *Cognitive, Affective, & Behavioral Neuroscience*. https://doi.org/10.3758/s13415-024-01236-2

Cavanagh, J. F., Eisenberg, I., Guitart-Masip, M., Huys, Q., & Frank, M. J. (2013). Frontal theta overrides Pavlovian learning biases. *Journal of Neuroscience*, *33*(19), 8541–8548. https://doi.org/10.1523/JNEUROSCI.5754-12.2013

Gideon, N., Hawkes, N., Mond, J., Saunders, R., Tchanturia, K., & Serpell, L. (2016). Development and psychometric validation of the EDE-QS, a 12 item short form of the Eating Disorder Examination Questionnaire (EDE-Q). *PLOS ONE*, *11*(5). https://doi.org/10.1371/journal.pone.0152744

Gillan, C. M., Kosinski, M., Whelan, R., Phelps, E. A., & Daw, N. D. (2016). Characterizing a psychiatric symptom dimension related to deficits in goal-directed control. *eLife*, *5*. https://doi.org/10.7554/eLife.11305.001

Heatherton, T. F., Kozlowski, L. T., Frecker, R. C., & Fagerström, K.-O. (1991). The Fagerström Test for Nicotine Dependence: A revision of the Fagerstrom Tolerance Questionnaire. *British Journal of Addiction*, *86*(9), 1119–1127. https://doi.org/10.1111/j.1360-0443.1991.tb01879.x

Kahveci, S., Bathke, A. C., & Blechert, J. (2024). Reaction-time task reliability is more accurately computed with permutation-based split-half correlations than with Cronbach’s alpha. *Psychonomic Bulletin & Review*. https://doi.org/10.3758/s13423-024-02597-y

Kessler, R. C., Adler, L., Ames, M., Demler, O., Faraone, S., Hiripi, E., Howes, M. J., Jin, R., Secnik, K., Spencer, T., Ustun, T. B., & Walters, E. E. (2005). The World Health Organization adult ADHD self-report scale (ASRS): A short screening scale for use in the general population. *Psychological Medicine*, *35*(2), 245–256. https://doi.org/10.1017/S0033291704002892

Lovibond, S. H., & Lovibond, P. F. (1995). *Manual for the Depression Anxiety Stress Scales* (2nd ed.). Psychology Foundation.

Mahlberg, J., Seabrooke, T., Weidemann, G., Hogarth, L., Mitchell, C. J., & Moustafa, A. A. (2021). Human appetitive Pavlovian-to-instrumental transfer: A goal-directed account. *Psychological Research*, *85*(2), 449–463. https://doi.org/10.1007/s00426-019-01266-3

Mkrtchian, A., Aylward, J., Dayan, P., Roiser, J. P., & Robinson, O. J. (2017). Modeling avoidance in mood and anxiety disorders using reinforcement learning. *Biological Psychiatry*, *82*(7), 532–539. https://doi.org/10.1016/j.biopsych.2017.01.017

Mkrtchian, A., Valton, V., & Roiser, J. P. (2023). Reliability of decision-making and reinforcement learning computational parameters. *Computational Psychiatry*, *7*(1), 30–46. https://doi.org/10.5334/cpsy.86

Montagnese, M., Knolle, F., Haarsma, J., Griffin, J. D., Richards, A., Vertes, P. E., Kiddle, B., Fletcher, P. C., Jones, P. B., Owen, M. J., Fonagy, P., Bullmore, E. T., Dolan, R. J., Moutoussis, M., Goodyer, I. M., & Murray, G. K. (2020). Reinforcement learning as an intermediate phenotype in psychosis? Deficits sensitive to illness stage but not associated with polygenic risk of schizophrenia in the general population. *Schizophrenia Research*, *222*, 389–396. https://doi.org/10.1016/j.schres.2020.04.022

Moutoussis, M., Bullmore, E. T., Goodyer, I. M., Fonagy, P., Jones, P. B., Dolan, R. J., & Dayan, P. (2018). Change, stability, and instability in the Pavlovian guidance of behaviour from adolescence to young adulthood. *PLoS Computational Biology*, *14*(12). https://doi.org/10.1371/journal.pcbi.1006679

Patton, J. H., Stanford, M. S., & Barratt, E. S. (1995). Factor structure of the Barratt Impulsiveness Scale. *Journal of Clinical Psychology*, *51*(6), 768–774. https://doi.org/10.1002/1097-4679(199511)51:6<768::AID-JCLP2270510607>3.0.CO;2-1

Saunders, J. B., Aasland, O. G., Babor, T. F., De la Fuente, J. R., & Grant, M. (1993). Development of the Alcohol Use Disorders Identification Test (AUDIT): WHO collaborative project on early detection of persons with harmful alcohol consumption‐II. *Addiction*, *88*(6), 791–804. https://doi.org/10.1111/j.1360-0443.1993.tb02093.x

Scholz, V., Hook, R. W., Kandroodi, M. R., Algermissen, J., Ioannidis, K., Christmas, D., Valle, S., Robbins, T. W., Grant, J. E., Chamberlain, S. R., & Den Ouden, H. E. M. (2022). Cortical dopamine reduces the impact of motivational biases governing automated behaviour. *Neuropsychopharmacology*, *47*, 1503–1512. https://doi.org/10.1038/s41386-022-01291-8

Steinglass, J. E., Lempert, K. M., Choo, T. H., Kimeldorf, M. B., Wall, M., Walsh, B. T., Fyer, A. J., Schneier, F. R., & Simpson, H. B. (2017). Temporal discounting across three psychiatric disorders: Anorexia nervosa, obsessive compulsive disorder, and social anxiety disorder. *Depression and Anxiety*, *34*(5), 463–470. https://doi.org/10.1002/da.22586

Wilson, R. C., & Collins, A. G. E. (2019). Ten simple rules for the computational modeling of behavioral data. *eLife*, *8*. https://doi.org/10.7554/eLife.49547

Zorowitz, S., Karni, G., Paredes, N., Daw, N., & Niv, Y. (2023). *Improving the reliability of the Pavlovian go/no-go task*. http://dx.doi.org/10.31234/osf.io/eb697

Zorowitz, S., & Niv, Y. (2023). Improving the reliability of cognitive task measures: A narrative review. *Biological Psychiatry: Cognitive Neuroscience and Neuroimaging*, *8*(8), 789–797. https://doi.org/10.1016/j.bpsc.2023.02.004

Zsido, A. N., Teleki, S. A., Csokasi, K., Rozsa, S., & Bandi, S. A. (2020). Development of the short version of the spielberger state—Trait anxiety inventory. *Psychiatry Research*, *291*, 113223. https://doi.org/10.1016/j.psychres.2020.113223

1. Participants were instructed to give the reward that they found most attractive rank 1, and the reward that they found least attractive rank 2, or, if they found both rewards equally attractive, to give both rewards rank 1. As a result, difference scores (delayed versus immediate) could be 1 (immediate valued more), 0 (both valued equally), or -1 (delayed valued more). [↑](#footnote-ref-1)
2. In Burghoorn et al. (2024), we compared two different parameter and model recovery methods: One that assessed recovery across the *full* range of plausible parameter values, and one that assessed recovery across the range of *actually observed* per-participant best-fitting parameter values. Recovery was generally improved with the latter compared to the former method, but the conclusion that recovery was more successful for M3 than for M4 and M5 was consistent across both methods. Since successful recovery in the range of observed parameter values has been argued to be more essential for model validation than successful recovery across the full range of parameter values (Wilson & Collins, 2019), we only examined recovery across the range of observed parameter values in the present study. [↑](#footnote-ref-2)
